# Supplementary figures and images for: Evolutionary persistence in Gunnera and the contribution of southern plant groups to the tropical Andes biodiversity hotspot
Source: PeerJ. 2018 Mar 16;6:e4388. doi: 10.7717/peerj.4388 (PMC5858603; doi:10.7717/peerj.4388)

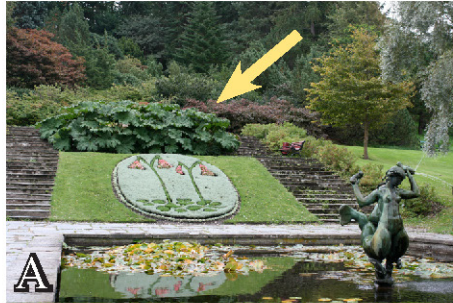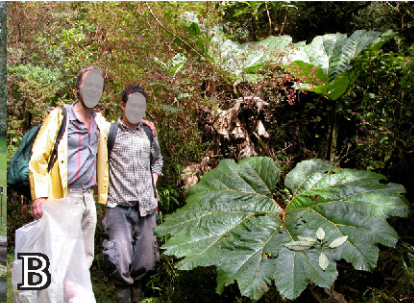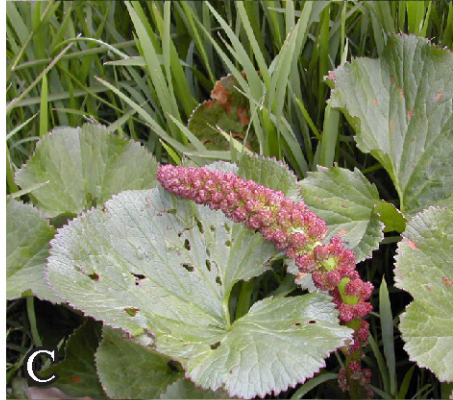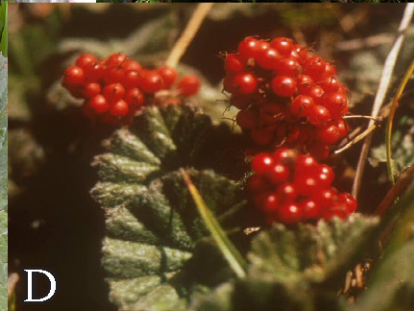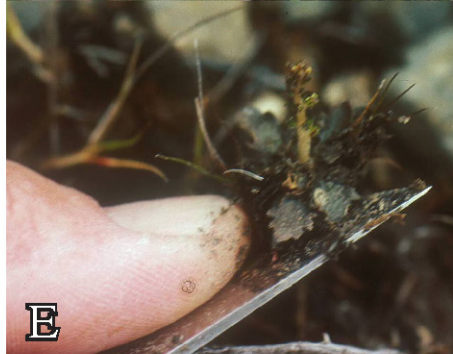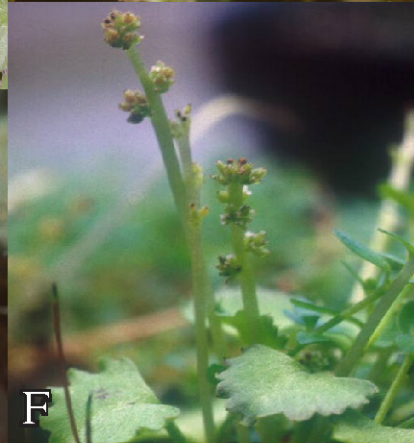

Supplement: Figure S1 — (A) G. manicata (subgenus Panke), (B) G. insignis (subgenus Panke), (C) G. perpensa (subgenus Gunnera), (D) G. magellanica (subgenus Misandra), (E) G. dentata (subgenus Milligania), (F) G. herteri (subgenus Ostenigunnera). Photos: A. Antonelli. [file peerj-06-4388-s001.pdf]

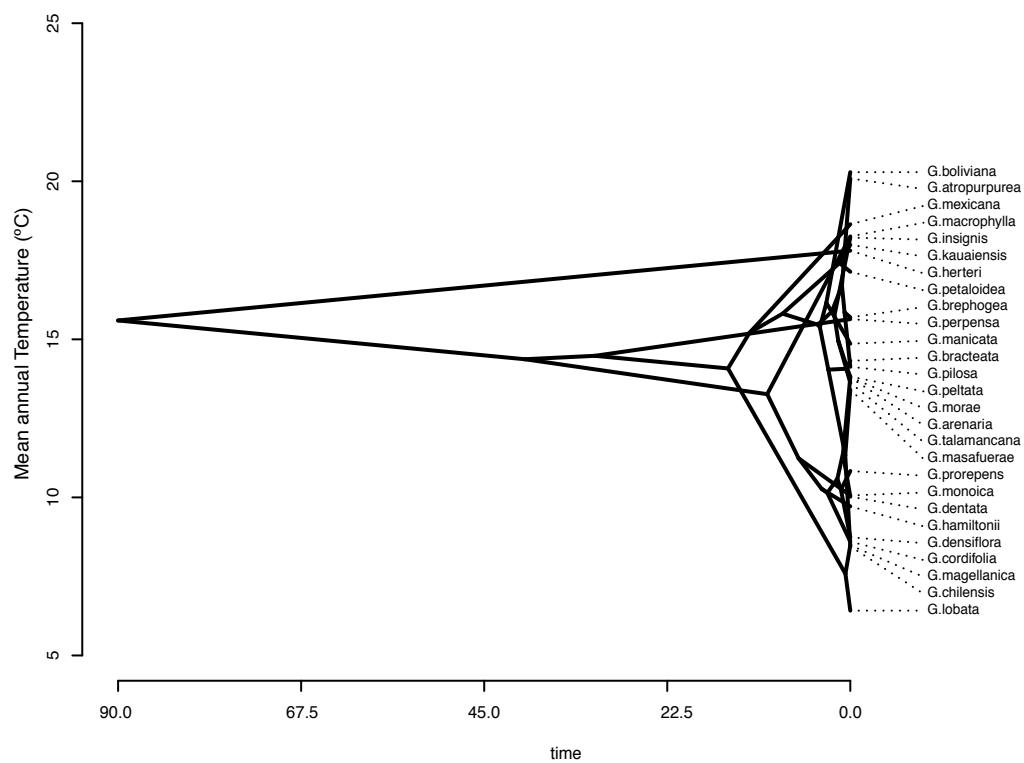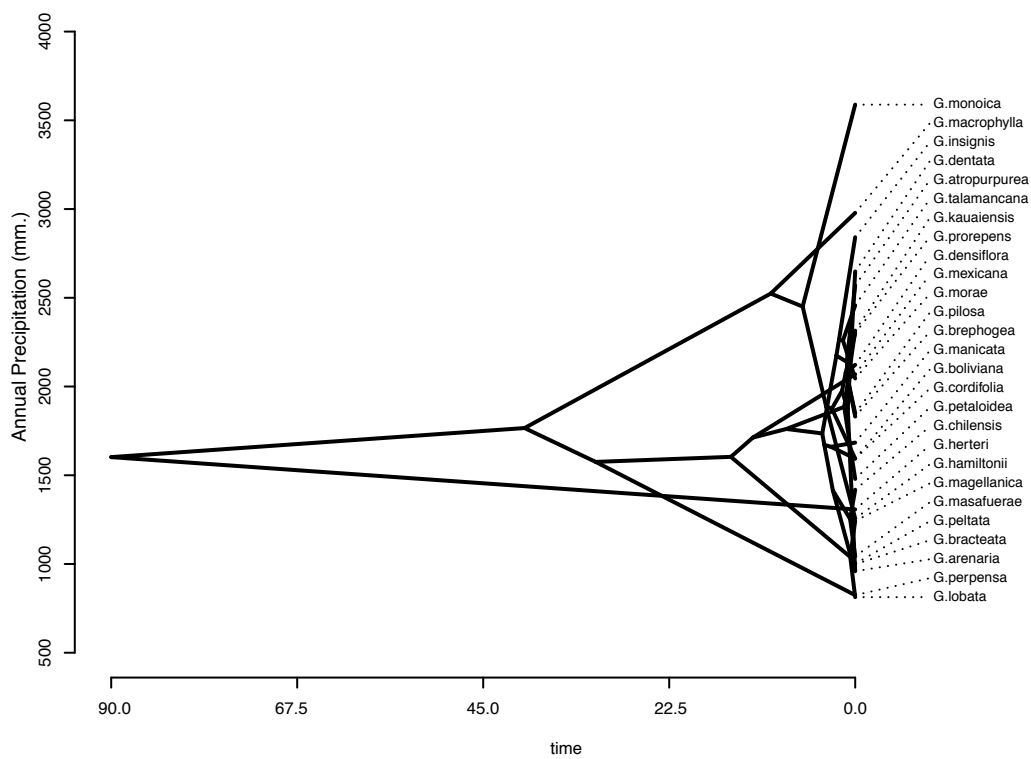

Supplement: Figure S2 — Black lines correspond to a projection of the phylogenetic tree in a space defined by the bioclimatic variable mean annual temperature and annual precipitation. [file peerj-06-4388-s002.pdf]
