# Supplementary material for: Evolutionary persistence in Gunnera and the contribution of southern plant groups to the tropical Andes biodiversity hotspot
Source: PeerJ. 2018 Mar 16;6:e4388. doi: 10.7717/peerj.4388 (PMC5858603; doi:10.7717/peerj.4388)
Supplement: Table S1 [file peerj-06-4388-s003.pdf]

Table S1. Database of extant and extinct *Gunnera* species.

**Data Publisher**	**Scientific name**
GBIF-Sweden	Gunnera morae Wanntorp & Klack.
Royal Botanic Garden Edinburgh	Gunnera perpensa L.
GBIF-Sweden	Gunnera bracteata Steud. ex Benn.
National Museum of Natural History, Smithsonian Institution	Gunnera perpensa
Bernice Pauahi Bishop Museum	Gunnera petaloidea
SysTax	Gunnera manicata Linden
Bernice Pauahi Bishop Museum	Gunnera petaloidea
MNHN - Museum national d'Histoire naturelle	gunnera macrophylla
SysTax	Gunnera magellanica Lam.
Instituto de Investigaci√≥n de Recursos Biol√≥gicos Alexander von Humboldt	Gunnera
South African National Biodiversity Institute	Gunnera perpensa
Missouri Botanical Garden	Gunnera L.
GBIF-Sweden	Gunnera perpensa
Yale University Peabody Museum	Gunnera monoica
National Museum of Natural History, Smithsonian Institution	Gunnera steyermarkii
Yale University Peabody Museum	Gunnera perpensa
Missouri Botanical Garden	Gunnera magellanica Lam.
National Museum of Natural History, Smithsonian Institution	Gunnera pilosa
Royal Botanic Gardens, Kew	Gunnera lobata Hook.f.
National Museum of Natural History, Smithsonian Institution	Gunnera manicata
Yale University Peabody Museum	Gunnera herteri
National Museum of Natural History, Smithsonian Institution	Gunnera scabra
GBIF-Sweden	Gunnera perpensa L.
GBIF New Zealand	Gunnera monoica
National Museum of Natural History, Smithsonian Institution	Gunnera herteri
GBIF New Zealand	Gunnera dentata Kirk
GBIF-Sweden	Gunnera hamiltoni
GBIF-Sweden	Gunnera bracteata Steud. ex Benn.
Administraci√≥n de Parques Nacionales, Argentina	Gunnera magellanica
National Museum of Natural History, Smithsonian Institution	Gunnera petaloidea var. kauaense
South African National Biodiversity Institute	Gunnera perpensa
Yale University Peabody Museum	Gunnera mexicana

Bernice Pauahi Bishop Museum	Gunnera petaloidea
GBIF-Sweden	Gunnera perpensa
MNHN - Museum national d'Histoire naturelle	gunnera magellanica
SysTax	Gunnera manicata Linden
Instituto de Bot√°nica Darwinion - CONICET	Gunnera lobata J.D. Hook.
MNHN - Museum national d'Histoire naturelle	gunnera perpensa
Bernice Pauahi Bishop Museum	Gunnera petaloidea
GBIF-Sweden	Gunnera perpensa L. var. kilimandscharica Schindl.
MNHN - Museum national d'Histoire naturelle	gunnera pilosa
MNHN - Museum national d'Histoire naturelle	gunnera magellanica
Bernice Pauahi Bishop Museum	Gunnera kauaiensis
MNHN - Museum national d'Histoire naturelle	gunnera manicata
MNHN - Museum national d'Histoire naturelle	gunnera lobata
Administraci√≥n de Parques Nacionales, Argentina	Gunnera magellanica
National Museum of Natural History, Smithsonian Institution	Gunnera talamancana
Bernice Pauahi Bishop Museum	Gunnera petaloidea
Missouri Botanical Garden	Gunnera lobata Hook. f.
Yale University Peabody Museum	Gunnera talamancana
South African National Biodiversity Institute	Gunnera perpensa
South African National Biodiversity Institute	Gunnera perpensa
GBIF-Sweden	Gunnera colombiana L. E. Mora
South African National Biodiversity Institute	Gunnera perpensa
National Museum of Natural History, Smithsonian Institution	Gunnera manicata
National Museum of Natural History, Smithsonian Institution	Gunnera insignis
GBIF-Sweden	Gunnera mixta Kirk
The New York Botanical Garden	Gunnera manicata Linden
Berkeley Natural History Museums	Gunnera mixta Kirk
MNHN - Museum national d'Histoire naturelle	gunnera pilosa
SysTax	Gunnera manicata Linden
Royal Botanic Gardens, Kew	Gunnera cordifolia (Hook.f.) Hook.f.
GBIF-Sweden	Gunnera bolivari J. F. Macbr.
GBIF-Spain	Gunnera bracteata Steud. ex J. Benn.
GBIF New Zealand	Gunnera densiflora Hook.f.
Harvard University Herbaria	Gunnera herteri Osten
South African National Biodiversity Institute	Gunnera perpensa

National Museum of Natural History, Smithsonian Institution	Gunnera lobata
National Museum of Natural History, Smithsonian Institution	Gunnera peltata
Bernice Pauahi Bishop Museum	Gunnera petaloidea
Missouri Botanical Garden	Gunnera colombiana L.E. Mora
GBIF-Sweden	Gunnera bracteata Steud. ex Benn.
National Museum of Natural History, Smithsonian Institution	Gunnera annae
GBIF-Sweden	Gunnera macrophylla Blume
GBIF-Sweden	Gunnera petaloidea Gaudich.
GBIF-Spain	Gunnera peltata Phil.
GBIF New Zealand	Gunnera monoica
Yale University Peabody Museum	Gunnera monoica
Royal Botanic Gardens, Kew	Gunnera mixta Kirk
Missouri Botanical Garden	Gunnera magellanica Lam.
Administraci√≥n de Parques Nacionales, Argentina	Gunnera magellanica
Administraci√≥n de Parques Nacionales, Argentina	Gunnera magellanica
National Museum of Natural History, Smithsonian Institution	Gunnera manicata
National Museum of Natural History, Smithsonian Institution	Gunnera insignis
National Museum of Natural History, Smithsonian Institution	Gunnera tajumbina
National Museum of Natural History, Smithsonian Institution	Gunnera perpensa
Bernice Pauahi Bishop Museum	Gunnera petaloidea subsp. petaloidea
Administraci√≥n de Parques Nacionales, Argentina	Gunnera magellanica
National Museum of Natural History, Smithsonian Institution	Gunnera bracteata
Bernice Pauahi Bishop Museum	Gunnera petaloidea
South African National Biodiversity Institute	Gunnera perpensa
South African National Biodiversity Institute	Gunnera perpensa
MNHN - Museum national d'Histoire naturelle	gunnera magellanica
Royal Botanic Gardens, Kew	Gunnera petaloidea Gaud.
MNHN - Museum national d'Histoire naturelle	gunnera petaloidea
MNHN - Museum national d'Histoire naturelle	gunnera rheifolia
GBIF-Sweden	Gunnera brephogea Linden & Andr√©
GBIF New Zealand	Gunnera densiflora Hook.f.
Bernice Pauahi Bishop Museum	Gunnera petaloidea
Berkeley Natural History Museums	Gunnera chilensis Lam.
GBIF-Sweden	Gunnera petaloidea Gaudich.
South African National Biodiversity Institute	Gunnera perpensa

MNHN - Museum national d'Histoire naturelle	gunnera macrophylla
National Museum of Natural History, Smithsonian Institution	Gunnera pilosa
National Museum of Natural History, Smithsonian Institution	Gunnera antioquensis
The New York Botanical Garden	Gunnera steyermarkii L. E. Mora
GBIF-Sweden	Gunnera masafuerae Skottsb.
GBIF-Sweden	Gunnera petaloidea Gaudich.
National Museum of Natural History, Smithsonian Institution	Gunnera pilosa
National Museum of Natural History, Smithsonian Institution	Gunnera magnifica
Netherlands Centre for Biodiversity Naturalis, section National Herbarium of the Netherlands	Gunnera magellanica Lam.
Missouri Botanical Garden	Gunnera pilosa Kunth
SysTax	Gunnera magellanica Lam.
Bernice Pauahi Bishop Museum	Gunnera petaloidea
Missouri Botanical Garden	Gunnera magellanica Lam.
MNHN - Museum national d'Histoire naturelle	gunnera magellanica
Instituto de Investigaci√≥n de Recursos Biol√≥gicos Alexander von Humboldt	Gunnera brephogea
Herbario SANT, Universidade de Santiago de Compostela	Gunnera magellanica Lam.
Bernice Pauahi Bishop Museum	Gunnera petaloidea
Instituto de Investigaci√≥n de Recursos Biol√≥gicos Alexander von Humboldt	Gunnera
Finnish Museum of Natural History	Gunnera manicata
GBIF-Sweden	Gunnera morae Wanntorp & Klack.
Missouri Botanical Garden	Gunnera insignis (Oerst.) A. DC.
Missouri Botanical Garden	Gunnera magnifica H. St. John
South African National Biodiversity Institute	Gunnera perpensa
Harvard University Herbaria	Gunnera bracteata Steudel ex Bennett
South African National Biodiversity Institute	Gunnera perpensa
National Museum of Natural History, Smithsonian Institution	Gunnera macrophylla
Instituto de Investigaci√≥n de Recursos Biol√≥gicos Alexander von Humboldt	Gunnera pilosa
MNHN - Museum national d'Histoire naturelle	gunnera magellanica
Bernice Pauahi Bishop Museum	Gunnera petaloidea
GBIF New Zealand	Gunnera albocarpa
Netherlands Centre for Biodiversity Naturalis, section National Herbarium of the Netherlands	Gunnera chilensis Lam.
Instituto de Investigaci√≥n de Recursos Biol√≥gicos Alexander von Humboldt	Gunnera atropurpurea
MNHN - Museum national d'Histoire naturelle	gunnera pilosa
Harvard University Herbaria	Gunnera atropurpurea var. munchicana L. E. Mora
South African National Biodiversity Institute	Gunnera perpensa

GBIF-Spain	Gunnera chilensis Lam.
Missouri Botanical Garden	Gunnera margaretae Schindler
Yale University Peabody Museum	Gunnera killipania
National Museum of Natural History, Smithsonian Institution	Gunnera brephogea
National Museum of Natural History, Smithsonian Institution	Gunnera chilensis
Bernice Pauahi Bishop Museum	Gunnera petaloidea
Instituto de Investigaci√≥n de Recursos Biol√≥gicos Alexander von Humboldt	Gunnera
Instituto de Investigaci√≥n de Recursos Biol√≥gicos Alexander von Humboldt	Gunnera
GBIF New Zealand	Gunnera prorepens
University of British Columbia	Gunnera prorepens Hook.f.
GBIF-Sweden	Gunnera peltata Phil.
Royal Botanic Gardens, Kew	Gunnera cordifolia (Hook.f.) Hook.f.
Missouri Botanical Garden	Gunnera magellanica Lam.
Missouri Botanical Garden	Gunnera magellanica Lam.
GBIF-Sweden	Gunnera herteri Osten
The New York Botanical Garden	Gunnera boliviana Morong
Royal Botanic Gardens, Kew	Gunnera brephogea Linden & Andr√©
GBIF-Sweden	Gunnera peltata Phil.
MNHN - Museum national d'Histoire naturelle	gunnera perpensa
MNHN - Museum national d'Histoire naturelle	gunnera macrophylla
Missouri Botanical Garden	Gunnera mexicana Brandegee
Instituto de Investigaci√≥n de Recursos Biol√≥gicos Alexander von Humboldt	Gunnera brephogea
Bernice Pauahi Bishop Museum	Gunnera petaloidea
GBIF-Spain	Gunnera pilosa
Missouri Botanical Garden	Gunnera magellanica Lam.
MNHN - Museum national d'Histoire naturelle	gunnera manicata
GBIF New Zealand	Gunnera monoica
Missouri Botanical Garden	Gunnera mexicana Brandegee
Instituto de Investigaci√≥n de Recursos Biol√≥gicos Alexander von Humboldt	Gunnera talamancana
Missouri Botanical Garden	Gunnera annae Schindl.
GBIF-Sweden	Gunnera magellanica Lam.
South African National Biodiversity Institute	Gunnera perpensa
National Museum of Natural History, Smithsonian Institution	Gunnera petaloidea
GBIF-Sweden	Gunnera herteri Osten
GBIF-Sweden	Gunnera magellanica Lam.

The New York Botanical Garden	Gunnera bolivari J. F. Macbr.
National Museum of Natural History, Smithsonian Institution	Gunnera petaloidea
GBIF-Sweden	Gunnera annae Schindl.
GBIF-Sweden	Gunnera scabra
Missouri Botanical Garden	Gunnera magellanica Lam.
SysTax	Gunnera manicata Linden
Yale University Peabody Museum	Gunnera magellanica
MNHN - Museum national d'Histoire naturelle	gunnera perpensa
MNHN - Museum national d'Histoire naturelle	gunnera lobata
South African National Biodiversity Institute	Gunnera perpensa
South African National Biodiversity Institute	Gunnera perpensa
South African National Biodiversity Institute	Gunnera perpensa
MNHN - Museum national d'Histoire naturelle	gunnera petaloidea
California Academy of Sciences	Gunnera mexicana Brandegee
MNHN - Museum national d'Histoire naturelle	gunnera chilensis
MNHN - Museum national d'Histoire naturelle	gunnera pilosa
Royal Botanic Gardens, Kew	Gunnera macrophylla Blume
Bernice Pauahi Bishop Museum	Gunnera macrophylla
Royal Botanic Garden Edinburgh	Gunnera berteroi Phil.
California Academy of Sciences	Gunnera killipiana Lundell
GBIF New Zealand	Gunnera hamiltonii Kirk
GBIF-Sweden	Gunnera insignis (Oerst.) Oerst.
SysTax	Gunnera manicata Linden
The New York Botanical Garden	Gunnera manicata Linden
Instituto de Investigaci√≥n de Recursos Biol√≥gicos Alexander von Humboldt	Gunnera brephogea
Administraci√≥n de Parques Nacionales, Argentina	Gunnera magellanica
Herbaria of the University and ETH Z√ºrich (Z+ZT)	Gunnera perpensa L.
Bernice Pauahi Bishop Museum	Gunnera petaloidea
Bernice Pauahi Bishop Museum	Gunnera petaloidea
Royal Botanic Garden Edinburgh	Gunnera hamiltonii Kirk
GBIF-Sweden	Gunnera herteri Osten
Royal Botanic Gardens, Kew	Gunnera monoica Raoul
National Museum of Natural History, Smithsonian Institution	Gunnera hernandezii
South African National Biodiversity Institute	Gunnera perpensa
MNHN - Museum national d'Histoire naturelle	gunnera brephogea

MNHN - Museum national d'Histoire naturelle	gunnera perpensa
Instituto de Investigaci√≥n de Recursos Biol√≥gicos Alexander von Humboldt	Gunnera magnifica
California Academy of Sciences	Gunnera mexicana Brandegee
Royal Botanic Garden Edinburgh	Gunnera berteroi Phil.
Bernice Pauahi Bishop Museum	Gunnera petaloidea
SysTax	Gunnera manicata Linden
SysTax	Gunnera manicata Linden
Bernice Pauahi Bishop Museum	Gunnera petaloidea
GBIF-Sweden	Gunnera perpensa L.
GBIF New Zealand	Gunnera prorepens
Royal Botanic Gardens, Kew	Gunnera monoica Raoul
GBIF-Sweden	Gunnera perpensa L.
GBIF-Sweden	Gunnera strigosa (Kirk) Colenso
National Museum of Natural History, Smithsonian Institution	Gunnera steyermarkii
Missouri Botanical Garden	Gunnera magellanica Lam.
Botanic Garden and Botanical Museum Berlin-Dahlem	Gunnera scabra Ruiz & Pav.
GBIF-Sweden	Gunnera colombiana L. E. Mora
GBIF-Sweden	Gunnera herteri Osten
The New York Botanical Garden	Gunnera masafuerae Skottsb.
National Museum of Natural History, Smithsonian Institution	Gunnera macrophylla
National Museum of Natural History, Smithsonian Institution	Gunnera magallanica
MNHN - Museum national d'Histoire naturelle	gunnera perpensa
MNHN - Museum national d'Histoire naturelle	gunnera perpensa
Netherlands Centre for Biodiversity Naturalis, section National Herbarium of the Netherlands	Gunnera wendlandii Reinke ex Schindl.
GBIF-Sweden	Gunnera bracteata Steud. ex Benn.
GBIF-Sweden	Gunnera magellanica Lam.
Royal Botanic Garden Edinburgh	Gunnera dentata Kirk
Yale University Peabody Museum	Gunnera lobata
National Museum of Natural History, Smithsonian Institution	Gunnera killipiana
Netherlands Centre for Biodiversity Naturalis, section National Herbarium of the Netherlands	Gunnera sp.
National Museum of Natural History, Smithsonian Institution	Gunnera insignis
National Museum of Natural History, Smithsonian Institution	Gunnera bracteata
Missouri Botanical Garden	Gunnera magellanica Lam.
Missouri Botanical Garden	Gunnera pilosa Kunth
Royal Botanic Garden Edinburgh	Gunnera berteroi Phil.

South African National Biodiversity Institute	Gunnera perpensa
MNHN - Museum national d'Histoire naturelle	gunnera perpensa
MNHN - Museum national d'Histoire naturelle	gunnera brephogea
MNHN - Museum national d'Histoire naturelle	gunnera magellanica
Bernice Pauahi Bishop Museum	Gunnera macrophylla
Bernice Pauahi Bishop Museum	Gunnera petaloidea
South African National Biodiversity Institute	Gunnera perpensa
Instituto de Investigaci√≥n de Recursos Biol√≥gicos Alexander von Humboldt	Gunnera
GBIF-Spain	Gunnera pilosa ?
Missouri Botanical Garden	Gunnera brephogea Linden & Andr√©
Missouri Botanical Garden	Gunnera annae Schindl.
Instituto de Investigaci√≥n de Recursos Biol√≥gicos Alexander von Humboldt	Gunnera magellanica
GBIF New Zealand	Gunnera monoica
South African National Biodiversity Institute	Gunnera perpensa
South African National Biodiversity Institute	Gunnera perpensa
South African National Biodiversity Institute	Gunnera perpensa
GBIF-Spain	Gunnera masafuerae Skottsb.
Missouri Botanical Garden	Gunnera brephogea Linden & Andr√©
Missouri Botanical Garden	Gunnera manicata Linden ex Andr√©
GBIF-Sweden	Gunnera morae Wanntorp & Klack.
GBIF New Zealand	Gunnera densiflora Hook.f.
National Museum of Natural History, Smithsonian Institution	Gunnera atropurpurea
National Museum of Natural History, Smithsonian Institution	Gunnera petaloidea
GBIF-Sweden	Gunnera bracteata Steud. ex Benn.
Bernice Pauahi Bishop Museum	Gunnera petaloidea
Yale University Peabody Museum	Gunnera killipania
GBIF New Zealand	Gunnera albocarpa
SysTax	Gunnera perpensa
Missouri Botanical Garden	Gunnera insignis (Oerst.) A. DC.
GBIF-Sweden	Gunnera herteri Osten
Royal Botanic Gardens, Kew	Gunnera monoica Raoul
GBIF-Sweden	Gunnera perpensa L.
GBIF-Sweden	Gunnera manicata Linden ex Delchev.
Instituto de Bot√°nica Darwinion - CONICET	Gunnera herteri Ost.
MNHN - Museum national d'Histoire naturelle	gunnera magellanica

National Museum of Natural History, Smithsonian Institution	Gunnera monoica
South African National Biodiversity Institute	Gunnera perpensa
MNHN - Museum national d'Histoire naturelle	gunnera magellanica
Missouri Botanical Garden	Gunnera margaretae Schindler
South African National Biodiversity Institute	Gunnera perpensa
GBIF-Sweden	Gunnera colombiana L. E. Mora
GBIF-Sweden	Gunnera petaloidea Gaudich.
Arboretum, University of Copenhagen	Gunnera magellanica
GBIF-Sweden	Gunnera kauaiensis Rock
GBIF-Sweden	Gunnera dentata Kirk
National Museum of Natural History, Smithsonian Institution	Gunnera pilosa
National Museum of Natural History, Smithsonian Institution	Gunnera pilosa
National Museum of Natural History, Smithsonian Institution	Gunnera pilosa
National Museum of Natural History, Smithsonian Institution	Gunnera insignis
Bioversity International	Gunnera magellanica Lam.
SysTax	Gunnera monoica Raoul
Instituto de Investigaci√≥n de Recursos Biol√≥gicos Alexander von Humboldt	Gunnera
GBIF-Sweden	Gunnera colombiana L. E. Mora
GBIF-Sweden	Gunnera prorepens Hook. f.
Bernice Pauahi Bishop Museum	Gunnera
National Museum of Natural History, Smithsonian Institution	Gunnera insignis
National Museum of Natural History, Smithsonian Institution	Gunnera petaloidea
National Museum of Natural History, Smithsonian Institution	Gunnera apiculata
National Museum of Natural History, Smithsonian Institution	Gunnera manicata
Netherlands Centre for Biodiversity Naturalis, section National Herbarium of the Netherlands	Gunnera magellanica Lam.
GBIF New Zealand	Gunnera monoica
SysTax	Gunnera manicata Linden
GBIF-Sweden	Gunnera brephogea Linden & Andr√©
GBIF-Sweden	Gunnera perpensa L.
National Museum of Natural History, Smithsonian Institution	Gunnera pilosa
GBIF New Zealand	Gunnera hamiltonii Kirk
Administraci√≥n de Parques Nacionales, Argentina	Gunnera magellanica
Administraci√≥n de Parques Nacionales, Argentina	Gunnera magellanica
Missouri Botanical Garden	Gunnera margaretae Schindler
GBIF-Sweden	Gunnera magellanica Lam.

GBIF-Sweden	Gunnera arenaria Cheeseman ex Kirk
National Museum of Natural History, Smithsonian Institution	Gunnera pilosa
National Museum of Natural History, Smithsonian Institution	Gunnera masafuerae
National Museum of Natural History, Smithsonian Institution	Gunnera manicata
MNHN - Museum national d'Histoire naturelle	gunnera magellanica
MNHN - Museum national d'Histoire naturelle	gunnera macrophylla
MNHN - Museum national d'Histoire naturelle	gunnera chilensis
The New York Botanical Garden	Gunnera manicata Linden
Administraci√≥n de Parques Nacionales, Argentina	Gunnera magellanica
MNHN - Museum national d'Histoire naturelle	gunnera bracteata
California Academy of Sciences	Gunnera mexicana Brandegee
Instituto de Investigaci√≥n de Recursos Biol√≥gicos Alexander von Humboldt	Gunnera
National Museum of Natural History, Smithsonian Institution	Gunnera magallanica
GBIF-Sweden	Gunnera perpensa L.
Bernice Pauahi Bishop Museum	Gunnera petaloidea
Harvard University Herbaria	Gunnera killipiana Lundell
National Museum of Natural History, Smithsonian Institution	Gunnera petaloidea
National Museum of Natural History, Smithsonian Institution	Gunnera macrophylla
National Museum of Natural History, Smithsonian Institution	Gunnera chilensis var. valdiviensis
Netherlands Centre for Biodiversity Naturalis, section National Herbarium of the Netherlands	Gunnera pilosa Kunth
Herbario SANT, Universidade de Santiago de Compostela	Gunnera magellanica Lam.
Berkeley Natural History Museums	Gunnera
GBIF New Zealand	Gunnera hamiltonii Kirk
Missouri Botanical Garden	Gunnera peltata Phil.
Missouri Botanical Garden	Gunnera magellanica Lam.
GBIF-Sweden	Gunnera magellanica Lam.
The New York Botanical Garden	Gunnera manicata Linden
Administraci√≥n de Parques Nacionales, Argentina	Gunnera magellanica
Yale University Peabody Museum	Gunnera talamancana
GBIF-Sweden	Gunnera brephogea Linden & Andr√©
GBIF New Zealand	Gunnera densiflora Hook.f.
National Museum of Natural History, Smithsonian Institution	Gunnera magallanica
Arboretum, University of Copenhagen	Gunnera chilensis
Missouri Botanical Garden	Gunnera herteri Mattf.
Royal Botanic Gardens, Kew	Gunnera prorepens Hook.f.

South African National Biodiversity Institute	Gunnera perpensa
MNHN - Museum national d'Histoire naturelle	gunnera macrophylla
MNHN - Museum national d'Histoire naturelle	gunnera magellanica
Yale University Peabody Museum	Gunnera magellanica
South African National Biodiversity Institute	Gunnera perpensa
South African National Biodiversity Institute	Gunnera perpensa
MNHN - Museum national d'Histoire naturelle	gunnera petaloidea
South African National Biodiversity Institute	Gunnera perpensa
Missouri Botanical Garden	Gunnera peltata Phil.
Missouri Botanical Garden	Gunnera brephogea Linden & Andr√©
MNHN - Museum national d'Histoire naturelle	gunnera perpensa
Administraci√≥n de Parques Nacionales, Argentina	Gunnera magellanica
Royal Botanic Gardens, Kew	Gunnera magellanica Lam.
Missouri Botanical Garden	Gunnera tayrona L.E. Mora
Yale University Peabody Museum	Gunnera lobata
GBIF-Spain	Gunnera magellanica Lam.
GBIF-Sweden	Gunnera magellanica Lam.
National Museum of Natural History, Smithsonian Institution	Gunnera petaloidea
Royal Botanic Gardens, Kew	Gunnera macrophylla Bl.
National Museum of Natural History, Smithsonian Institution	Gunnera magallanica
Missouri Botanical Garden	Gunnera molokaiensis H. St. John
Instituto de Investigaci√≥n de Recursos Biol√≥gicos Alexander von Humboldt	Gunnera
Bernice Pauahi Bishop Museum	Gunnera petaloidea
Bernice Pauahi Bishop Museum	Gunnera petaloidea
Bernice Pauahi Bishop Museum	Gunnera petaloidea
Instituto de Investigaci√≥n de Recursos Biol√≥gicos Alexander von Humboldt	Gunnera pilosa
GBIF New Zealand	Gunnera monoica Raoul
Yale University Peabody Museum	Gunnera prorepens
GBIF New Zealand	Gunnera hamiltonii Kirk
GBIF-Sweden	Gunnera monoica Raoul
GBIF-Sweden	Gunnera perpensa L.
SysTax	Gunnera manicata Linden
Administraci√≥n de Parques Nacionales, Argentina	Gunnera magellanica
MNHN - Museum national d'Histoire naturelle	gunnera magellanica
MNHN - Museum national d'Histoire naturelle	gunnera magellanica

National Museum of Natural History, Smithsonian Institution	Gunnera magallanica
South African National Biodiversity Institute	Gunnera perpensa
Bernice Pauahi Bishop Museum	Gunnera petaloidea
SysTax	Gunnera isignis (Oerst.) A.DC.
GBIF-Sweden	Gunnera pilosa Kunth
National Museum of Natural History, Smithsonian Institution	Gunnera bracteata
Bernice Pauahi Bishop Museum	Gunnera petaloidea
Bernice Pauahi Bishop Museum	Gunnera petaloidea
GBIF-Sweden	Gunnera prorepens Hook. f.
National Museum of Natural History, Smithsonian Institution	Gunnera chilensis
Instituto de Investigaci√≥n de Recursos Biol√≥gicos Alexander von Humboldt	Gunnera
MNHN - Museum national d'Histoire naturelle	gunnera magellanica
Yale University Peabody Museum	Gunnera hamiltonii
Bernice Pauahi Bishop Museum	Gunnera petaloidea
Instituto de Bot√°nica Darwinion - CONICET	Gunnera tinctoria (Molina) Mirb. var. valdiviensis
Yale University Peabody Museum	Gunnera monoica
Bernice Pauahi Bishop Museum	Gunnera petaloidea
Bernice Pauahi Bishop Museum	Gunnera petaloidea subsp. petaloidea
GBIF-Sweden	Gunnera insignis (Oerst.) Oerst.
GBIF-Sweden	Gunnera magellanica Lam.
National Museum of Natural History, Smithsonian Institution	Gunnera brephogea
National Museum of Nature and Science, Japan	Gunnera
The New York Botanical Garden	Gunnera manicata Linden
Administraci√≥n de Parques Nacionales, Argentina	Gunnera magellanica
Missouri Botanical Garden	Gunnera manicata Linden ex Andr√©
South African National Biodiversity Institute	Gunnera perpensa
National Museum of Natural History, Smithsonian Institution	Gunnera brephogea
Bernice Pauahi Bishop Museum	Gunnera petaloidea subsp. kauaiensis
GBIF New Zealand	Gunnera monoica
Missouri Botanical Garden	Gunnera tamanensis L.E. Mora
GBIF New Zealand	Gunnera hamiltonii Kirk
Netherlands Centre for Biodiversity Naturalis, section National Herbarium of the Netherlands	Gunnera eastwoodae H.St.John
MNHN - Museum national d'Histoire naturelle	gunnera mexicana
Royal Botanic Gardens, Kew	Gunnera prorepens Hook.f.
National Museum of Natural History, Smithsonian Institution	Gunnera magallanica

The New York Botanical Garden	Gunnera venezolana L. E. Mora & L. E. Mora subsp. tachirensis
Royal Botanic Gardens, Kew	Gunnera tinctoria Mirb. var. valdiviensis Mora
National Museum of Natural History, Smithsonian Institution	Gunnera talamancana
National Museum of Natural History, Smithsonian Institution	Gunnera insignis
GBIF-Sweden	Gunnera magellanica Lam.
Yale University Peabody Museum	Gunnera manicata
GBIF New Zealand	Gunnera monoica Raoul
GBIF New Zealand	Gunnera densiflora Hook.f.
Field Museum	Gunnera pilosa Kunth
National Museum of Natural History, Smithsonian Institution	Gunnera magallanica
National Museum of Natural History, Smithsonian Institution	Gunnera magallanica
GBIF New Zealand	Gunnera dentata Kirk
GBIF New Zealand	Gunnera cordifolia
SysTax	Gunnera manicata Linden
Bernice Pauahi Bishop Museum	Gunnera petaloidea
GBIF-Sweden	Gunnera monoica Raoul
Herbaria of the University and ETH Z√ºrich (Z+ZT)	Gunnera boliviana Morong. ex Rusby
National Museum of Natural History, Smithsonian Institution	Gunnera eastwoodae
MNHN - Museum national d'Histoire naturelle	gunnera pilosa
MNHN - Museum national d'Histoire naturelle	gunnera chilensis
GBIF New Zealand	Gunnera monoica
California Academy of Sciences	Gunnera herteri Osten
GBIF New Zealand	Gunnera densiflora Hook.f.
GBIF-Sweden	Gunnera tajumbina L. E. Mora
GBIF New Zealand	Gunnera hamiltonii Kirk
National Museum of Natural History, Smithsonian Institution	Gunnera brephogea
National Museum of Natural History, Smithsonian Institution	Gunnera pilosa
National Museum of Natural History, Smithsonian Institution	Gunnera macrophylla
Bernice Pauahi Bishop Museum	Gunnera macrophylla
Arboretum, University of Copenhagen	Gunnera magellanica
MNHN - Museum national d'Histoire naturelle	gunnera bracteata
SysTax	Gunnera magellanica Lam.
GBIF-Sweden	Gunnera perpensa L.
Berkeley Natural History Museums	Gunnera
GBIF New Zealand	Gunnera prorepens

South African National Biodiversity Institute	Gunnera perpensa
Missouri Botanical Garden	Gunnera peltata Phil.
MNHN - Museum national d'Histoire naturelle	gunnera magellanica
SysTax	Gunnera magellanica Lam.
GBIF-Sweden	Gunnera tajumbina L. E. Mora
National Museum of Natural History, Smithsonian Institution	Gunnera dentata
National Museum of Natural History, Smithsonian Institution	Gunnera magallanica
Instituto de Bot√°nica Darwinion - CONICET	Gunnera herterii Osten
MNHN - Museum national d'Histoire naturelle	gunnera macrophylla
MNHN - Museum national d'Histoire naturelle	gunnera lobata
MNHN - Museum national d'Histoire naturelle	gunnera perpensa
MNHN - Museum national d'Histoire naturelle	gunnera magellanica
MNHN - Museum national d'Histoire naturelle	gunnera macrophylla
Botanic Garden and Botanical Museum Berlin-Dahlem	Gunnera plicata
GBIF-Sweden	Gunnera brephogea Linden & Andr√©
GBIF-Sweden	Gunnera perpensa L.
The New York Botanical Garden	Gunnera amicorum Ewan
Administraci√≥n de Parques Nacionales, Argentina	Gunnera magellanica
Administraci√≥n de Parques Nacionales, Argentina	Gunnera magellanica
National Museum of Natural History, Smithsonian Institution	Gunnera insignis
National Museum of Natural History, Smithsonian Institution	Gunnera masafuerae
National Museum of Natural History, Smithsonian Institution	Gunnera macrophylla
National Museum of Natural History, Smithsonian Institution	Gunnera peltata
Berkeley Natural History Museums	Gunnera mixta Kirk
Netherlands Centre for Biodiversity Naturalis, section National Herbarium of the Netherlands	Gunnera manicata Linden
MNHN - Museum national d'Histoire naturelle	gunnera pilosa
MNHN - Museum national d'Histoire naturelle	gunnera chilensis
GBIF-Spain	Gunnera magellanica Lam.
GBIF-Sweden	Gunnera bracteata Steud. ex Benn.
SysTax	Gunnera manicata Linden
Instituto de Investigaci√≥n de Recursos Biol√≥gicos Alexander von Humboldt	Gunnera talamancana
IHAR	Gunnera chilensis Lam.
National Museum of Natural History, Smithsonian Institution	Gunnera macrophylla
National Museum of Natural History, Smithsonian Institution	Gunnera insignis
Bernice Pauahi Bishop Museum	Gunnera petaloidea

GBIF New Zealand	Gunnera albocarpa
South African National Biodiversity Institute	Gunnera perpensa
Instituto de Bot√°nica Darwinion - CONICET	Gunnera reichei Schindl.
MNHN - Museum national d'Histoire naturelle	gunnera perpensa
GBIF New Zealand	Gunnera monoica
Field Museum	Gunnera L.
SysTax	Gunnera perpensa
South African National Biodiversity Institute	Gunnera perpensa
South African National Biodiversity Institute	Gunnera perpensa
MNHN - Museum national d'Histoire naturelle	gunnera magellanica
MNHN - Museum national d'Histoire naturelle	gunnera chilensis
GBIF-Sweden	Gunnera manicata Linden ex Delchev.
Royal Botanic Garden Edinburgh	Gunnera magellanica Lam.
GBIF-Sweden	Gunnera monoica Raoul
Herbaria of the University and ETH Z√ºrich (Z+ZT)	Gunnera boliviana Morong. ex Rusby
Field Museum	Gunnera L.
National Museum of Natural History, Smithsonian Institution	Gunnera chilensis
National Museum of Natural History, Smithsonian Institution	Gunnera petaloidea
GBIF-Sweden	Gunnera perpensa L.
GBIF-Sweden	Gunnera peltata Phil.
National Museum of Natural History, Smithsonian Institution	Gunnera bolivari
Field Museum	Gunnera herteri Osten
Missouri Botanical Garden	Gunnera magellanica Lam.
Missouri Botanical Garden	Gunnera colombiana L.E. Mora
Missouri Botanical Garden	Gunnera L.
Missouri Botanical Garden	Gunnera magellanica Lam.
Missouri Botanical Garden	Gunnera magellanica Lam.
Staatliche Naturwissenschaftliche Sammlungen Bayerns	Gunnera talamancana H.Weber & L.E.Mora
Instituto de Investigaci√≥n de Recursos Biol√≥gicos Alexander von Humboldt	Gunnera
Missouri Botanical Garden	Gunnera colombiana L.E. Mora
National Museum of Nature and Science, Japan	Gunnera talamancana Weber & L. E. Mora
South African National Biodiversity Institute	Gunnera perpensa
MNHN - Museum national d'Histoire naturelle	gunnera perpensa
MNHN - Museum national d'Histoire naturelle	gunnera petaloidea
Harvard University Herbaria	Gunnera mexicana Brandegee

Royal Botanic Garden Edinburgh	Gunnera magellanica Lam.
SysTax	Gunnera magellanica Lam.
National Museum of Natural History, Smithsonian Institution	Gunnera manicata
National Museum of Natural History, Smithsonian Institution	Gunnera brephogea
Botanic Garden and Botanical Museum Berlin-Dahlem	Gunnera mexicana Brandegee
Berkeley Natural History Museums	Gunnera insignis Oerst.
GBIF-Sweden	Gunnera pilosa Kunth
National Museum of Natural History, Smithsonian Institution	Gunnera lobata
National Museum of Natural History, Smithsonian Institution	Gunnera pilosa
GBIF-Spain	Gunnera
Yale University Peabody Museum	Gunnera dentata
GBIF-Sweden	Gunnera brephogea Linden & Andr√©
GBIF New Zealand	Gunnera dentata
GBIF New Zealand	Gunnera monoica
National Museum of Natural History, Smithsonian Institution	Gunnera petaloidea
National Museum of Natural History, Smithsonian Institution	Gunnera macrophylla
Missouri Botanical Garden	Gunnera pilosa Kunth
Missouri Botanical Garden	Gunnera magellanica Lam.
Royal Botanic Gardens, Kew	Gunnera prorepens Hook.f.
South African National Biodiversity Institute	Gunnera perpensa
MNHN - Museum national d'Histoire naturelle	gunnera magellanica
MNHN - Museum national d'Histoire naturelle	gunnera magellanica
Royal Botanic Gardens, Kew	Gunnera cordifolia (Hook.f.) Hook.f.
Bernice Pauahi Bishop Museum	Gunnera petaloidea
Bernice Pauahi Bishop Museum	Gunnera petaloidea
South African National Biodiversity Institute	Gunnera perpensa
South African National Biodiversity Institute	Gunnera perpensa
MNHN - Museum national d'Histoire naturelle	gunnera perpensa
MNHN - Museum national d'Histoire naturelle	gunnera kaalensis
SysTax	Gunnera manicata Linden
Bernice Pauahi Bishop Museum	Gunnera petaloidea
Arboretum, University of Copenhagen	Gunnera magellanica
GBIF-Spain	Gunnera scabra Ruiz & Pav.
Bernice Pauahi Bishop Museum	Gunnera petaloidea
Instituto de Investigaci√≥n de Recursos Biol√≥gicos Alexander von Humboldt	Gunnera magnifica

South African National Biodiversity Institute	Gunnera perpensa
South African National Biodiversity Institute	Gunnera perpensa
South African National Biodiversity Institute	Gunnera perpensa
SysTax	Gunnera manicata Linden
Bernice Pauahi Bishop Museum	Gunnera macrophylla
Bernice Pauahi Bishop Museum	Gunnera masafuerae
Bernice Pauahi Bishop Museum	Gunnera petaloidea
Royal Botanic Garden Edinburgh	Gunnera arenaria Cheeseman ex Kirk
GBIF-Sweden	Gunnera mixta Kirk
GBIF-Sweden	Gunnera cordifolia (Hook. f.) Hook. f.
Harvard University Herbaria	Gunnera bracteata Steudel ex Bennett
Bioversity International	Gunnera monoica
National Museum of Natural History, Smithsonian Institution	Gunnera pilosa
Bernice Pauahi Bishop Museum	Gunnera petaloidea
GBIF-Sweden	Gunnera perpensa
Instituto de Bot√°nica Darwinion - CONICET	Gunnera apiculata Schindl.
SysTax	Gunnera manicata Linden
SysTax	Gunnera manicata Linden
GBIF-Sweden	Gunnera pilosa Kunth
National Museum of Natural History, Smithsonian Institution	Gunnera prorepens
Missouri Botanical Garden	Gunnera magellanica Lam.
GBIF New Zealand	Gunnera hamiltonii Kirk
GBIF-Sweden	Gunnera petaloidea Gaudich.
Missouri Botanical Garden	Gunnera brephogea Linden & Andr√©
South African National Biodiversity Institute	Gunnera perpensa
MNHN - Museum national d'Histoire naturelle	gunnera magellanica
MNHN - Museum national d'Histoire naturelle	gunnera magellanica
SysTax	Gunnera magellanica Lam.
Instituto de Investigaci√≥n de Recursos Biol√≥gicos Alexander von Humboldt	Gunnera
Missouri Botanical Garden	Gunnera magellanica Lam.
Missouri Botanical Garden	Gunnera aequatoriensis L.E. Mora
MNHN - Museum national d'Histoire naturelle	gunnera perpensa
Instituto de Bot√°nica Darwinion - CONICET	Gunnera glabra Phil.
MNHN - Museum national d'Histoire naturelle	gunnera magellanica
GBIF New Zealand	Gunnera monoica

GBIF-Sweden	Gunnera brephogea Linden & Andr√©
Netherlands Centre for Biodiversity Naturalis, section National Herbarium of the Netherlands	Gunnera macrophylla Blume
Bernice Pauahi Bishop Museum	Gunnera petaloidea
Administraci√≥n de Parques Nacionales, Argentina	Gunnera magellanica
National Museum of Natural History, Smithsonian Institution	Gunnera insignis
The New York Botanical Garden	Gunnera columbiana L. E. Mora
Missouri Botanical Garden	Gunnera magellanica Lam.
Staatliche Naturwissenschaftliche Sammlungen Bayerns	Gunnera magellanica Lam.
Missouri Botanical Garden	Gunnera magellanica Lam.
The New York Botanical Garden	Gunnera magellanica Lam.
Instituto de Bot√°nica Darwinion - CONICET	Gunnera magellanica Lam.
Instituto de Bot√°nica Darwinion - CONICET	Gunnera magellanica Lam.
University of Connecticut	Gunnera magellanica Lam.
Instituto de Bot√°nica Darwinion - CONICET	Gunnera magellanica Lam.
National Museum of Natural History, Smithsonian Institution	Gunnera magallanica
Instituto de Bot√°nica Darwinion - CONICET	Gunnera magellanica Lam.
Instituto de Bot√°nica Darwinion - CONICET	Gunnera magellanica Lam.
Instituto de Bot√°nica Darwinion - CONICET	Gunnera magellanica Lam.
Missouri Botanical Garden	Gunnera magellanica Lam.
Instituto de Bot√°nica Darwinion - CONICET	Gunnera magellanica Lam.
Missouri Botanical Garden	Gunnera magellanica Lam.
Missouri Botanical Garden	Gunnera magellanica Lam.
British Antarctic Survey	Gunnera lobata
Instituto de Bot√°nica Darwinion - CONICET	Gunnera lobata Hook. f.
Instituto de Bot√°nica Darwinion - CONICET	Gunnera magellanica Lam.
Instituto de Bot√°nica Darwinion - CONICET	Gunnera lobata Hook. f.
Instituto de Bot√°nica Darwinion - CONICET	Gunnera magellanica Lam.
British Antarctic Survey	Gunnera magellanica
Missouri Botanical Garden	Gunnera lobata Hook. f.
Missouri Botanical Garden	Gunnera lobata Hook. f.
Missouri Botanical Garden	Gunnera magellanica Lam.
Missouri Botanical Garden	Gunnera magellanica Lam.
GBIF-Spain	Gunnera magellanica Lam.
Royal Botanic Garden Edinburgh	Gunnera magellanica Lam.
GBIF-Spain	Gunnera magellanica Lam.

Instituto de Bot√°nica Darwinion - CONICET	Gunnera magellanica Lam.
Missouri Botanical Garden	Gunnera magellanica Lam.
Instituto de Bot√°nica Darwinion - CONICET	Gunnera magellanica Lam.
University of Connecticut	Gunnera magellanica Lam.
Museo Argentino de Ciencias Naturales	Gunnera magellanica Lam.
British Antarctic Survey	Gunnera magellanica
British Antarctic Survey	Gunnera magellanica
British Antarctic Survey	Gunnera magellanica
Museo Argentino de Ciencias Naturales	Gunnera magellanica Lam.
Museo Argentino de Ciencias Naturales	Gunnera magellanica Lam.
British Antarctic Survey	Gunnera magellanica
Museo Argentino de Ciencias Naturales	Gunnera magellanica Lam.
British Antarctic Survey	Gunnera magellanica
Museo Argentino de Ciencias Naturales	Gunnera
British Antarctic Survey	Gunnera magellanica
British Antarctic Survey	Gunnera magellanica
Museo Argentino de Ciencias Naturales	Gunnera
Museo Argentino de Ciencias Naturales	Gunnera
Museo Argentino de Ciencias Naturales	Gunnera
GBIF-Spain	Gunnera lobata Hook.f.
Missouri Botanical Garden	Gunnera magellanica Lam.
Instituto de Bot√°nica Darwinion - CONICET	Gunnera magellanica Lam.
Instituto de Bot√°nica Darwinion - CONICET	Gunnera magellanica Lam.
Instituto de Bot√°nica Darwinion - CONICET	Gunnera magellanica Lam.
Instituto de Bot√°nica Darwinion - CONICET	Gunnera magellanica Lam.
Administraci√≥n de Parques Nacionales, Argentina	Gunnera magellanica
Administraci√≥n de Parques Nacionales, Argentina	Gunnera magellanica
Administraci√≥n de Parques Nacionales, Argentina	Gunnera magellanica
GBIF-Sweden	Gunnera lobata Hook. f.
GBIF New Zealand	Gunnera L.
GBIF New Zealand	Gunnera dentata Kirk
GBIF New Zealand	Gunnera prorepens Hook.f.
Royal Botanic Garden Edinburgh	Gunnera magellanica Lam.
Royal Botanic Garden Edinburgh	Gunnera magellanica Lam.
Royal Botanic Garden Edinburgh	Gunnera tinctoria Mirb.

Instituto de Bot√°nica Darwinion - CONICET	Gunnera reicheri Schindl.
GBIF New Zealand	Gunnera albocarpa
GBIF New Zealand	Gunnera monoica
GBIF New Zealand	Gunnera albocarpa
GBIF New Zealand	Gunnera albocarpa
GBIF New Zealand	Gunnera albocarpa
GBIF New Zealand	Gunnera monoica
GBIF New Zealand	Gunnera monoica
GBIF New Zealand	Gunnera albocarpa
GBIF New Zealand	Gunnera monoica
GBIF New Zealand	Gunnera monoica
GBIF New Zealand	Gunnera albocarpa
GBIF New Zealand	Gunnera albocarpa
GBIF New Zealand	Gunnera albocarpa
GBIF New Zealand	Gunnera albocarpa
GBIF New Zealand	Gunnera monoica
GBIF New Zealand	Gunnera monoica
GBIF New Zealand	Gunnera albocarpa
GBIF New Zealand	Gunnera albocarpa
GBIF New Zealand	Gunnera prorepens Hook.f.
Staatliche Naturwissenschaftliche Sammlungen Bayerns	Gunnera magellanica Lam.
GBIF New Zealand	Gunnera albocarpa
GBIF New Zealand	Gunnera albocarpa
GBIF New Zealand	Gunnera monoica
GBIF New Zealand	Gunnera albocarpa
GBIF New Zealand	Gunnera albocarpa
GBIF New Zealand	Gunnera albocarpa
GBIF New Zealand	Gunnera albocarpa
GBIF New Zealand	Gunnera albocarpa
GBIF New Zealand	Gunnera albocarpa
GBIF New Zealand	Gunnera albocarpa
GBIF New Zealand	Gunnera albocarpa
GBIF New Zealand	Gunnera albocarpa
GBIF New Zealand	Gunnera albocarpa
GBIF New Zealand	Gunnera albocarpa

GBIF New Zealand	Gunnera monoica
University of British Columbia	Gunnera monica raoul
GBIF New Zealand	Gunnera albocarpa
GBIF New Zealand	Gunnera albocarpa
GBIF New Zealand	Gunnera albocarpa
GBIF New Zealand	Gunnera albocarpa
GBIF New Zealand	Gunnera albocarpa
GBIF New Zealand	Gunnera monoica
GBIF New Zealand	Gunnera albocarpa
GBIF New Zealand	Gunnera albocarpa
GBIF New Zealand	Gunnera monoica
GBIF New Zealand	Gunnera monoica
GBIF New Zealand	Gunnera monoica
GBIF New Zealand	Gunnera monoica
GBIF New Zealand	Gunnera monoica
GBIF New Zealand	Gunnera monoica
GBIF New Zealand	Gunnera monoica
GBIF New Zealand	Gunnera monoica
GBIF New Zealand	Gunnera monoica
GBIF New Zealand	Gunnera monoica
GBIF New Zealand	Gunnera monoica
GBIF New Zealand	Gunnera monoica
GBIF New Zealand	Gunnera monoica
GBIF New Zealand	Gunnera monoica
GBIF New Zealand	Gunnera monoica
GBIF New Zealand	Gunnera monoica
GBIF New Zealand	Gunnera √ómixta Kirk
GBIF New Zealand	Gunnera monoica
GBIF New Zealand	Gunnera monoica
GBIF New Zealand	Gunnera monoica
GBIF New Zealand	Gunnera monoica
GBIF New Zealand	Gunnera monoica Raoul
GBIF New Zealand	Gunnera monoica
GBIF New Zealand	Gunnera monoica
GBIF New Zealand	Gunnera monoica

GBIF New Zealand	Gunnera dentata
GBIF New Zealand	Gunnera monoica
GBIF New Zealand	Gunnera monoica
GBIF New Zealand	Gunnera monoica
GBIF New Zealand	Gunnera monoica
GBIF New Zealand	Gunnera prorepens Hook.f.
GBIF New Zealand	Gunnera dentata
GBIF New Zealand	Gunnera monoica
GBIF New Zealand	Gunnera dentata
GBIF New Zealand	Gunnera arenaria
GBIF New Zealand	Gunnera monoica
GBIF New Zealand	Gunnera monoica
GBIF New Zealand	Gunnera monoica
GBIF New Zealand	Gunnera monoica
GBIF New Zealand	Gunnera monoica
GBIF New Zealand	Gunnera monoica
Australian National Herbarium (CANB)	Gunnera prorepens
GBIF New Zealand	Gunnera monoica
GBIF New Zealand	Gunnera monoica
GBIF New Zealand	Gunnera monoica
GBIF New Zealand	Gunnera monoica
GBIF New Zealand	Gunnera monoica
GBIF New Zealand	Gunnera monoica
GBIF New Zealand	Gunnera monoica
GBIF New Zealand	Gunnera monoica
GBIF New Zealand	Gunnera monoica
GBIF New Zealand	Gunnera monoica
GBIF New Zealand	Gunnera monoica
GBIF New Zealand	Gunnera monoica
GBIF New Zealand	Gunnera monoica
GBIF New Zealand	Gunnera monoica
GBIF New Zealand	Gunnera dentata
GBIF New Zealand	Gunnera monoica
GBIF New Zealand	Gunnera monoica
GBIF New Zealand	Gunnera monoica

GBIF New Zealand	Gunnera monoica
GBIF New Zealand	Gunnera monoica
GBIF New Zealand	Gunnera monoica
GBIF New Zealand	Gunnera monoica
GBIF New Zealand	Gunnera dentata
GBIF New Zealand	Gunnera monoica
GBIF New Zealand	Gunnera monoica
GBIF New Zealand	Gunnera monoica
GBIF New Zealand	Gunnera monoica
GBIF New Zealand	Gunnera albocarpa
GBIF New Zealand	Gunnera monoica
GBIF New Zealand	Gunnera monoica
GBIF New Zealand	Gunnera monoica Raoul
GBIF New Zealand	Gunnera monoica
GBIF New Zealand	Gunnera monoica
GBIF New Zealand	Gunnera monoica
GBIF New Zealand	Gunnera monoica
GBIF New Zealand	Gunnera monoica
GBIF New Zealand	Gunnera monoica
GBIF New Zealand	Gunnera monoica
GBIF New Zealand	Gunnera monoica
GBIF New Zealand	Gunnera monoica
GBIF New Zealand	Gunnera monoica Raoul
GBIF New Zealand	Gunnera monoica Raoul
GBIF New Zealand	Gunnera dentata
GBIF New Zealand	Gunnera monoica
GBIF New Zealand	Gunnera monoica
GBIF New Zealand	Gunnera monoica
GBIF New Zealand	Gunnera monoica
Royal Botanic Garden Edinburgh	Gunnera monoica Raoul
GBIF New Zealand	Gunnera monoica
GBIF New Zealand	Gunnera monoica
GBIF New Zealand	Gunnera monoica
GBIF New Zealand	Gunnera dentata
GBIF New Zealand	Gunnera monoica

GBIF New Zealand	Gunnera monoica
Royal Botanic Garden Edinburgh	Gunnera
GBIF New Zealand	Gunnera monoica
GBIF New Zealand	Gunnera monoica
GBIF New Zealand	Gunnera monoica
GBIF New Zealand	Gunnera dentata
GBIF New Zealand	Gunnera monoica
GBIF New Zealand	Gunnera monoica
GBIF New Zealand	Gunnera monoica
GBIF New Zealand	Gunnera monoica
GBIF New Zealand	Gunnera monoica
GBIF New Zealand	Gunnera monoica
GBIF New Zealand	Gunnera prorepens
GBIF New Zealand	Gunnera prorepens
GBIF New Zealand	Gunnera monoica
GBIF New Zealand	Gunnera albocarpa
GBIF New Zealand	Gunnera prorepens
GBIF New Zealand	Gunnera dentata
GBIF New Zealand	Gunnera prorepens
GBIF New Zealand	Gunnera albocarpa
GBIF New Zealand	Gunnera prorepens
GBIF New Zealand	Gunnera monoica Raoul
GBIF New Zealand	Gunnera monoica Raoul
GBIF New Zealand	Gunnera monoica Raoul
GBIF New Zealand	Gunnera monoica Raoul
GBIF New Zealand	Gunnera monoica Raoul
GBIF New Zealand	Gunnera monoica Raoul
GBIF New Zealand	Gunnera monoica Raoul
GBIF New Zealand	Gunnera monoica Raoul
Royal Botanic Garden Edinburgh	Gunnera monoica Raoul
GBIF New Zealand	Gunnera monoica Raoul
Royal Botanic Garden Edinburgh	Gunnera tinctoria Mirb.
Royal Botanic Garden Edinburgh	Gunnera magellanica Lam.
Royal Botanic Garden Edinburgh	Gunnera magellanica Lam.
Royal Botanic Garden Edinburgh	Gunnera magellanica Lam.

Royal Botanic Garden Edinburgh	Gunnera magellanica Lam.
Royal Botanic Garden Edinburgh	Gunnera manicata Lindl.
Royal Botanic Garden Edinburgh	Gunnera
Royal Botanic Garden Edinburgh	Gunnera
Royal Botanic Garden Edinburgh	Gunnera densiflora Hook.f.
Royal Botanic Garden Edinburgh	Gunnera densiflora Hook.f.
Royal Botanic Garden Edinburgh	Gunnera prorepens Hook.f.
GBIF New Zealand	Gunnera L.
Royal Botanic Garden Edinburgh	Gunnera monoica Raoul
GBIF New Zealand	Gunnera albocarpa (Kirk) Cockayne
GBIF New Zealand	Gunnera monoica
GBIF New Zealand	Gunnera √ómixta Kirk
GBIF New Zealand	Gunnera monoica Raoul
GBIF New Zealand	Gunnera prorepens Hook.f.
GBIF New Zealand	Gunnera monoica
GBIF New Zealand	Gunnera monoica
GBIF New Zealand	Gunnera prorepens Hook.f.
GBIF New Zealand	Gunnera monoica
GBIF New Zealand	Gunnera monoica
GBIF New Zealand	Gunnera monoica
GBIF New Zealand	Gunnera monoica
GBIF New Zealand	Gunnera monoica
GBIF New Zealand	Gunnera strigosa
GBIF New Zealand	Gunnera monoica
GBIF New Zealand	Gunnera dentata
GBIF New Zealand	Gunnera monoica
GBIF New Zealand	Gunnera monoica
GBIF New Zealand	Gunnera monoica
GBIF New Zealand	Gunnera monoica
GBIF New Zealand	Gunnera monoica
GBIF New Zealand	Gunnera monoica
GBIF New Zealand	Gunnera monoica
GBIF New Zealand	Gunnera monoica
GBIF New Zealand	Gunnera monoica Raoul
GBIF New Zealand	Gunnera monoica Raoul

GBIF New Zealand	Gunnera monoica
GBIF New Zealand	Gunnera monoica
GBIF New Zealand	Gunnera monoica
GBIF New Zealand	Gunnera monoica Raoul
GBIF New Zealand	Gunnera monoica Raoul
GBIF New Zealand	Gunnera monoica
GBIF New Zealand	Gunnera monoica
GBIF New Zealand	Gunnera monoica
GBIF New Zealand	Gunnera monoica
GBIF New Zealand	Gunnera monoica
GBIF New Zealand	Gunnera monoica
GBIF New Zealand	Gunnera monoica
GBIF New Zealand	Gunnera dentata Kirk
GBIF New Zealand	Gunnera prorepens Hook.f.
GBIF New Zealand	Gunnera monoica Raoul
GBIF New Zealand	Gunnera monoica
GBIF New Zealand	Gunnera prorepens Hook.f.
GBIF New Zealand	Gunnera √ómixta Kirk
GBIF New Zealand	Gunnera monoica Raoul
GBIF New Zealand	Gunnera monoica Raoul
GBIF New Zealand	Gunnera monoica Raoul
GBIF New Zealand	Gunnera dentata Kirk
British Antarctic Survey	Gunnera chilensis
Royal Botanic Garden Edinburgh	Gunnera magellanica Lam.
Royal Botanic Garden Edinburgh	Gunnera magellanica Lam.
Royal Botanic Garden Edinburgh	Gunnera magellanica Lam.
British Antarctic Survey	Gunnera magellanica
Royal Botanic Garden Edinburgh	Gunnera
Instituto de Bot√°nica Darwinion - CONICET	Gunnera tinctoria (Molina) Mirb. var. meyerii
Instituto de Bot√°nica Darwinion - CONICET	Gunnera tinctoria (Molina) Mirb. var. meyerii
Staatliche Naturwissenschaftliche Sammlungen Bayerns	Gunnera magellanica Lam.
Instituto de Bot√°nica Darwinion - CONICET	Gunnera tinctoria (Molina) Mirbel
Royal Botanic Garden Edinburgh	Gunnera cordifolia (Hook.f.) Hook.f.
Australian National Herbarium (CANB)	Gunnera cordifolia
GBIF-Sweden	Gunnera cordifolia (Hook. f.) Hook. f.

Australian National Herbarium (CANB)	Gunnera cordifolia
Australian National Herbarium (CANB)	Gunnera cordifolia
GBIF New Zealand	Gunnera monoica Raoul
GBIF New Zealand	Gunnera monoica Raoul
GBIF New Zealand	Gunnera monoica Raoul
GBIF New Zealand	Gunnera strigosa
Netherlands Centre for Biodiversity Naturalis, section National Herbarium of the Netherlands	Gunnera albocarpa (Kirk) Cockayne
GBIF New Zealand	Gunnera √ómixta Kirk
GBIF New Zealand	Gunnera monoica Raoul
GBIF New Zealand	Gunnera √ómixta Kirk
GBIF New Zealand	Gunnera dentata Kirk
GBIF New Zealand	Gunnera prorepens Hook.f.
Royal Botanic Garden Edinburgh	Gunnera tinctoria Mirb.
Instituto de Bot√°nica Darwinion - CONICET	Gunnera tinctoria (Mol.) Mirbel
Instituto de Bot√°nica Darwinion - CONICET	Gunnera tincytoria (Molina) Mirbel
Instituto de Bot√°nica Darwinion - CONICET	Gunnera tinctoria (Mol.) Mirbel
Instituto de Bot√°nica Darwinion - CONICET	Gunnera tinctoria (Mol.) Mirbel
Instituto de Bot√°nica Darwinion - CONICET	Gunnera magellanica Lam.
Instituto de Bot√°nica Darwinion - CONICET	Gunnera tinctoria (Mol.) Mirbel
University of Arizona Herbarium	Gunnera tinctoria
Missouri Botanical Garden	Gunnera tinctoria Mirb.
Instituto de Bot√°nica Darwinion - CONICET	Gunnera tincytoria (Molina) Mirbel
GBIF-Sweden	Gunnera cordifolia (Hook. f.) Hook. f.
GBIF-Sweden	Gunnera cordifolia (Hook. f.) Hook. f.
GBIF-Sweden	Gunnera cordifolia (Hook. f.) Hook. f.
GBIF-Sweden	Gunnera cordifolia (Hook. f.) Hook. f.
GBIF New Zealand	Gunnera dentata Kirk
GBIF New Zealand	Gunnera arenaria Cheeseman
GBIF New Zealand	Gunnera monoica
GBIF New Zealand	Gunnera monoica
GBIF New Zealand	Gunnera monoica
Royal Botanic Garden Edinburgh	Gunnera magellanica Lam.
GBIF New Zealand	Gunnera monoica
GBIF New Zealand	Gunnera dentata Kirk
GBIF New Zealand	Gunnera dentata Kirk

GBIF New Zealand	Gunnera manicata Linden ex Andr√©
Royal Botanic Garden Edinburgh	Gunnera tinctoria Mirb.
Instituto de Bot√°nica Darwinion - CONICET	Gunnera tinctoria (Molina) Mirb.
Australian National Herbarium (CANB)	Gunnera
Royal Botanic Garden Edinburgh	Gunnera magellanica Lam.
Royal Botanic Garden Edinburgh	Gunnera tinctoria Mirb.
Royal Botanic Garden Edinburgh	Gunnera flavida Colenso
Royal Botanic Garden Edinburgh	Gunnera prorepens Hook.f.
GBIF New Zealand	Gunnera √óstrigosa Colenso
GBIF-Spain	Gunnera tinctoria (Molina) Mirbel
GBIF-Spain	Gunnera tinctoria (Molina) Mirbel
Royal Botanic Garden Edinburgh	Gunnera magellanica Lam.
Missouri Botanical Garden	Gunnera magellanica Lam.
Missouri Botanical Garden	Gunnera tinctoria Mirb.
Instituto de Bot√°nica Darwinion - CONICET	Gunnera tinctoria (Molina) Mirb.
Royal Botanic Garden Edinburgh	Gunnera berteroi Phil.
Royal Botanic Garden Edinburgh	Gunnera
Royal Botanic Garden Edinburgh	Gunnera berteroi Phil.
Royal Botanic Garden Edinburgh	Gunnera berteroi Phil.
Royal Botanic Garden Edinburgh	Gunnera berteroi Phil.
Royal Botanic Garden Edinburgh	Gunnera berteroi Phil.
Royal Botanic Garden Edinburgh	Gunnera berteroi Phil.
Royal Botanic Garden Edinburgh	Gunnera berteroi Phil.
Royal Botanic Garden Edinburgh	Gunnera berteroi Phil.
Instituto de Bot√°nica Darwinion - CONICET	Gunnera herterii Osten
South African National Biodiversity Institute	Gunnera perpensa
Royal Botanic Garden Edinburgh	Gunnera bracteata Steud. ex J.Benn.
Royal Botanic Garden Edinburgh	Gunnera peltata Phil.
Royal Botanic Garden Edinburgh	Gunnera bracteata Steud. ex J.Benn.
Royal Botanic Garden Edinburgh	Gunnera peltata Phil.
Royal Botanic Garden Edinburgh	Gunnera peltata Phil.
GBIF-Sweden	Gunnera herteri Osten
South African National Biodiversity Institute	Gunnera perpensa
South African National Biodiversity Institute	Gunnera perpensa
South African National Biodiversity Institute	Gunnera perpensa

South African National Biodiversity Institute	Gunnera perpensa
South African National Biodiversity Institute	Gunnera perpensa
South African National Biodiversity Institute	Gunnera perpensa
South African National Biodiversity Institute	Gunnera perpensa
South African National Biodiversity Institute	Gunnera perpensa
South African National Biodiversity Institute	Gunnera perpensa
South African National Biodiversity Institute	Gunnera perpensa
South African National Biodiversity Institute	Gunnera perpensa
South African National Biodiversity Institute	Gunnera perpensa
South African National Biodiversity Institute	Gunnera perpensa
South African National Biodiversity Institute	Gunnera perpensa
South African National Biodiversity Institute	Gunnera perpensa
South African National Biodiversity Institute	Gunnera perpensa
South African National Biodiversity Institute	Gunnera perpensa
South African National Biodiversity Institute	Gunnera perpensa
South African National Biodiversity Institute	Gunnera perpensa
South African National Biodiversity Institute	Gunnera perpensa
South African National Biodiversity Institute	Gunnera perpensa
South African National Biodiversity Institute	Gunnera perpensa
Royal Botanic Garden Edinburgh	Gunnera
South African National Biodiversity Institute	Gunnera perpensa
South African National Biodiversity Institute	Gunnera perpensa
South African National Biodiversity Institute	Gunnera perpensa
South African National Biodiversity Institute	Gunnera perpensa
South African National Biodiversity Institute	Gunnera perpensa
South African National Biodiversity Institute	Gunnera perpensa
South African National Biodiversity Institute	Gunnera perpensa
South African National Biodiversity Institute	Gunnera perpensa
South African National Biodiversity Institute	Gunnera perpensa
South African National Biodiversity Institute	Gunnera perpensa
South African National Biodiversity Institute	Gunnera perpensa
South African National Biodiversity Institute	Gunnera perpensa
South African National Biodiversity Institute	Gunnera perpensa
South African National Biodiversity Institute	Gunnera perpensa
South African National Biodiversity Institute	Gunnera perpensa

South African National Biodiversity Institute	Gunnera perpensa
South African National Biodiversity Institute	Gunnera perpensa
South African National Biodiversity Institute	Gunnera perpensa
South African National Biodiversity Institute	Gunnera perpensa
South African National Biodiversity Institute	Gunnera perpensa
South African National Biodiversity Institute	Gunnera perpensa
South African National Biodiversity Institute	Gunnera perpensa
South African National Biodiversity Institute	Gunnera perpensa
South African National Biodiversity Institute	Gunnera perpensa
South African National Biodiversity Institute	Gunnera perpensa
Missouri Botanical Garden	Gunnera perpensa L.
South African National Biodiversity Institute	Gunnera perpensa
South African National Biodiversity Institute	Gunnera perpensa
South African National Biodiversity Institute	Gunnera perpensa
South African National Biodiversity Institute	Gunnera perpensa
South African National Biodiversity Institute	Gunnera perpensa
South African National Biodiversity Institute	Gunnera perpensa
South African National Biodiversity Institute	Gunnera perpensa
South African National Biodiversity Institute	Gunnera perpensa
South African National Biodiversity Institute	Gunnera perpensa
South African National Biodiversity Institute	Gunnera perpensa
South African National Biodiversity Institute	Gunnera perpensa
South African National Biodiversity Institute	Gunnera perpensa
South African National Biodiversity Institute	Gunnera perpensa
South African National Biodiversity Institute	Gunnera perpensa
South African National Biodiversity Institute	Gunnera perpensa
South African National Biodiversity Institute	Gunnera perpensa
Royal Botanic Garden Edinburgh	Gunnera perpensa L.
South African National Biodiversity Institute	Gunnera perpensa
South African National Biodiversity Institute	Gunnera perpensa
South African National Biodiversity Institute	Gunnera perpensa
South African National Biodiversity Institute	Gunnera perpensa
South African National Biodiversity Institute	Gunnera perpensa
South African National Biodiversity Institute	Gunnera perpensa
South African National Biodiversity Institute	Gunnera perpensa

South African National Biodiversity Institute	Gunnera perpensa
South African National Biodiversity Institute	Gunnera perpensa
National Museum of Natural History, Smithsonian Institution	Gunnera manicata
National Museum of Natural History, Smithsonian Institution	Gunnera manicata
National Museum of Natural History, Smithsonian Institution	Gunnera manicata
National Museum of Natural History, Smithsonian Institution	Gunnera manicata
National Herbarium of New South Wales	Gunnera perpense L.
South African National Biodiversity Institute	Gunnera perpensa
South African National Biodiversity Institute	Gunnera perpensa
South African National Biodiversity Institute	Gunnera perpensa
South African National Biodiversity Institute	Gunnera perpensa
South African National Biodiversity Institute	Gunnera perpensa
South African National Biodiversity Institute	Gunnera perpensa
South African National Biodiversity Institute	Gunnera perpensa
South African National Biodiversity Institute	Gunnera perpensa
South African National Biodiversity Institute	Gunnera perpensa
South African National Biodiversity Institute	Gunnera perpensa
South African National Biodiversity Institute	Gunnera perpensa
South African National Biodiversity Institute	Gunnera perpensa
South African National Biodiversity Institute	Gunnera perpensa
South African National Biodiversity Institute	Gunnera perpensa
South African National Biodiversity Institute	Gunnera perpensa
South African National Biodiversity Institute	Gunnera perpensa
South African National Biodiversity Institute	Gunnera perpensa
South African National Biodiversity Institute	Gunnera perpensa
South African National Biodiversity Institute	Gunnera perpensa
Field Museum	Gunnera magellanica Lam.
South African National Biodiversity Institute	Gunnera perpensa
South African National Biodiversity Institute	Gunnera perpensa
South African National Biodiversity Institute	Gunnera perpensa
South African National Biodiversity Institute	Gunnera perpensa
South African National Biodiversity Institute	Gunnera perpensa
South African National Biodiversity Institute	Gunnera perpensa
South African National Biodiversity Institute	Gunnera perpensa
South African National Biodiversity Institute	Gunnera perpensa

South African National Biodiversity Institute	Gunnera perpensa
South African National Biodiversity Institute	Gunnera perpensa
South African National Biodiversity Institute	Gunnera perpensa
South African National Biodiversity Institute	Gunnera perpensa
National Museum of Natural History, Smithsonian Institution	Gunnera perpensa
South African National Biodiversity Institute	Gunnera perpensa
South African National Biodiversity Institute	Gunnera perpensa
South African National Biodiversity Institute	Gunnera perpensa
South African National Biodiversity Institute	Gunnera perpensa
South African National Biodiversity Institute	Gunnera perpensa
South African National Biodiversity Institute	Gunnera perpensa
South African National Biodiversity Institute	Gunnera perpensa
South African National Biodiversity Institute	Gunnera perpensa
South African National Biodiversity Institute	Gunnera perpensa
South African National Biodiversity Institute	Gunnera perpensa
South African National Biodiversity Institute	Gunnera perpensa
South African National Biodiversity Institute	Gunnera perpensa
South African National Biodiversity Institute	Gunnera perpensa
South African National Biodiversity Institute	Gunnera perpensa
South African National Biodiversity Institute	Gunnera perpensa
South African National Biodiversity Institute	Gunnera perpensa
South African National Biodiversity Institute	Gunnera perpensa
South African National Biodiversity Institute	Gunnera perpensa
South African National Biodiversity Institute	Gunnera perpensa
South African National Biodiversity Institute	Gunnera perpensa
South African National Biodiversity Institute	Gunnera perpensa
South African National Biodiversity Institute	Gunnera perpensa
Instituto de Bot√°nica Darwinion - CONICET	Gunnera apiculata Schindler
Instituto de Bot√°nica Darwinion - CONICET	Gunnera apiculata Schindl.
Instituto de Bot√°nica Darwinion - CONICET	Gunnera
Instituto de Bot√°nica Darwinion - CONICET	Gunnera berteroi Phil.
South African National Biodiversity Institute	Gunnera perpensa
South African National Biodiversity Institute	Gunnera perpensa
South African National Biodiversity Institute	Gunnera perpensa
South African National Biodiversity Institute	Gunnera perpensa

South African National Biodiversity Institute	Gunnera perpensa
South African National Biodiversity Institute	Gunnera perpensa
South African National Biodiversity Institute	Gunnera perpensa
South African National Biodiversity Institute	Gunnera perpensa
South African National Biodiversity Institute	Gunnera perpensa
Instituto de Bot√°nica Darwinion - CONICET	Gunnera apiculata Schindl.
South African National Biodiversity Institute	Gunnera perpensa
Missouri Botanical Garden	Gunnera L.
Missouri Botanical Garden	Gunnera margaretae Schindler
Conservation International	Gunnera apiculata Schindl.
Missouri Botanical Garden	Gunnera margaretae Schindler
Missouri Botanical Garden	Gunnera schindleri L.E. Mora
GBIF-Sweden	Gunnera schindleri L.E.Mora
Missouri Botanical Garden	Gunnera apiculata Schindl.
Missouri Botanical Garden	Gunnera L.
Missouri Botanical Garden	Gunnera annae Schindl.
Missouri Botanical Garden	Gunnera annae Schindl.
Missouri Botanical Garden	Gunnera annae Schindl.
GBIF-Spain	Gunnera
Missouri Botanical Garden	Gunnera L.
Missouri Botanical Garden	Gunnera L.
Missouri Botanical Garden	Gunnera L.
Missouri Botanical Garden	Gunnera L.
Missouri Botanical Garden	Gunnera L.
Missouri Botanical Garden	Gunnera magellanica Lam.
Netherlands Centre for Biodiversity Naturalis, section National Herbarium of the Netherlands	Gunnera magellanica Lam.
Missouri Botanical Garden	Gunnera annae Schindl.
Missouri Botanical Garden	Gunnera apiculata Schindl.
Missouri Botanical Garden	Gunnera L.
Missouri Botanical Garden	Gunnera L.
Missouri Botanical Garden	Gunnera margaretae Schindler
Missouri Botanical Garden	Gunnera peruviana J.F. Macbr.
Netherlands Centre for Biodiversity Naturalis, section National Herbarium of the Netherlands	Gunnera magellanica Lam.
Missouri Botanical Garden	Gunnera magellanica Lam.
Missouri Botanical Garden	Gunnera berteroi Phil.

Missouri Botanical Garden	Gunnera scabra Ruiz & Pav.
Missouri Botanical Garden	Gunnera magellanica Lam.
Missouri Botanical Garden	Gunnera peruviana J.F. Macbr.
GBIF-Sweden	Gunnera boliviana Morong. ex Rusby
GBIF-Sweden	Gunnera boliviana Morong. ex Rusby
Missouri Botanical Garden	Gunnera L.
Missouri Botanical Garden	Gunnera magellanica Lam.
Missouri Botanical Garden	Gunnera L.
Missouri Botanical Garden	Gunnera magellanica Lam.
Missouri Botanical Garden	Gunnera magellanica Lam.
Missouri Botanical Garden	Gunnera magellanica Lam.
Missouri Botanical Garden	Gunnera L.
Missouri Botanical Garden	Gunnera berteroi Phil.
Missouri Botanical Garden	Gunnera peruviana J.F. Macbr.
Missouri Botanical Garden	Gunnera L.
Missouri Botanical Garden	Gunnera L.
Missouri Botanical Garden	Gunnera L.
Missouri Botanical Garden	Gunnera L.
Missouri Botanical Garden	Gunnera L.
Missouri Botanical Garden	Gunnera bolivari J.F. Macbr.
Missouri Botanical Garden	Gunnera L.
Field Museum	Gunnera L.
Missouri Botanical Garden	Gunnera L.
Missouri Botanical Garden	Gunnera L.
Missouri Botanical Garden	Gunnera L.
Missouri Botanical Garden	Gunnera L.
Missouri Botanical Garden	Gunnera L.
Missouri Botanical Garden	Gunnera brephogea subsp. magna L.E. Mora
Missouri Botanical Garden	Gunnera L.
Missouri Botanical Garden	Gunnera L.
Missouri Botanical Garden	Gunnera L.
Missouri Botanical Garden	Gunnera L.
Missouri Botanical Garden	Gunnera L.
South African National Biodiversity Institute	Gunnera perpensa
Missouri Botanical Garden	Gunnera annae Schindl.

Field Museum	Gunnera annae Schindl.
Field Museum	Gunnera scabra Ru√≠z & Pav.
Field Museum	Gunnera annae Schindl.
Field Museum	Gunnera bolivari J. F. Macbr.
Field Museum	Gunnera peruviana J. F. Macbr.
Field Museum	Gunnera peruviana J. F. Macbr.
Missouri Botanical Garden	Gunnera perpensa L.
Missouri Botanical Garden	Gunnera perpensa L.
National Museum of Natural History, Smithsonian Institution	Gunnera perpensa
National Museum of Natural History, Smithsonian Institution	Gunnera perpensa
National Herbarium of New South Wales	Gunnera macrophylla
National Museum of Natural History, Smithsonian Institution	Gunnera macrophylla
National Herbarium of New South Wales	Gunnera macrophylla
Australian National Herbarium (CANB)	Gunnera macrophylla
National Herbarium of New South Wales	Gunnera macrophylla
National Herbarium of New South Wales	Gunnera macrophylla
Australian National Herbarium (CANB)	Gunnera macrophylla
National Herbarium of New South Wales	Gunnera macrophylla
National Herbarium of New South Wales	Gunnera macrophylla
National Herbarium of New South Wales	Gunnera macrophylla
National Herbarium of New South Wales	Gunnera
National Herbarium of New South Wales	Gunnera macrophylla
Australian National Herbarium (CANB)	Gunnera macrophylla
National Herbarium of New South Wales	Gunnera macrophylla
National Herbarium of New South Wales	Gunnera
National Herbarium of New South Wales	Gunnera macrophylla
Missouri Botanical Garden	Gunnera L.
Missouri Botanical Garden	Gunnera L.
Missouri Botanical Garden	Gunnera L.
National Herbarium of New South Wales	Gunnera
National Herbarium of New South Wales	Gunnera macrophylla Blume
National Herbarium of New South Wales	Gunnera macrophylla
National Herbarium of New South Wales	Gunnera macrophylla
National Herbarium of New South Wales	Gunnera macrophylla
National Herbarium of New South Wales	Gunnera macrophylla Blume

National Museum of Natural History, Smithsonian Institution	Gunnera macrophylla
National Herbarium of New South Wales	Gunnera
National Herbarium of New South Wales	Gunnera
National Herbarium of New South Wales	Gunnera
National Herbarium of New South Wales	Gunnera
National Herbarium of New South Wales	Gunnera macrophylla
National Herbarium of New South Wales	Gunnera macrophylla
National Herbarium of New South Wales	Gunnera macrophylla
National Herbarium of New South Wales	Gunnera macrophylla
Missouri Botanical Garden	Gunnera annae Schindl.
Missouri Botanical Garden	Gunnera brephogea subsp. magna L.E. Mora
Missouri Botanical Garden	Gunnera bolivari J.F. Macbr.
Missouri Botanical Garden	Gunnera L.
Missouri Botanical Garden	Gunnera L.
National Herbarium of New South Wales	Gunnera macrophylla
National Herbarium of New South Wales	Gunnera
National Herbarium of New South Wales	Gunnera
National Herbarium of New South Wales	Gunnera
National Herbarium of New South Wales	Gunnera macrophylla
National Herbarium of New South Wales	Gunnera
National Museum of Natural History, Smithsonian Institution	Gunnera sp.
National Herbarium of New South Wales	Gunnera macrophylla
National Herbarium of New South Wales	Gunnera macrophylla Blume
Missouri Botanical Garden	Gunnera L.
National Herbarium of New South Wales	Gunnera macrophylla
Missouri Botanical Garden	Gunnera L.
National Herbarium of New South Wales	Gunnera macrophylla Blume
National Herbarium of New South Wales	Gunnera macrophylla
National Herbarium of New South Wales	Gunnera
National Herbarium of New South Wales	Gunnera
Herbarium of the University of Aarhus	Gunnera
Missouri Botanical Garden	Gunnera L.
Missouri Botanical Garden	Gunnera brephogea Linden & Andr√©
Missouri Botanical Garden	Gunnera L.
Missouri Botanical Garden	Gunnera pilosa Kunth

Herbarium of the University of Aarhus	Gunnera
Missouri Botanical Garden	Gunnera L.
Missouri Botanical Garden	Gunnera L.
Missouri Botanical Garden	Gunnera L.
National Museum of Natural History, Smithsonian Institution	Gunnera sp.
National Herbarium of New South Wales	Gunnera macrophylla
National Museum of Natural History, Smithsonian Institution	Gunnera macrophylla
National Museum of Natural History, Smithsonian Institution	Gunnera macrophylla
National Museum of Natural History, Smithsonian Institution	Gunnera macrophylla
Australian National Herbarium (CANB)	Gunnera macrophylla
National Herbarium of New South Wales	Gunnera macrophylla
National Museum of Natural History, Smithsonian Institution	Gunnera macrophylla
National Museum of Natural History, Smithsonian Institution	Gunnera macrophylla
Conservation International	Gunnera macrophylla Bl.
Missouri Botanical Garden	Gunnera L.
Missouri Botanical Garden	Gunnera L.
Missouri Botanical Garden	Gunnera L.
University of Connecticut	Gunnera annae Schindl.
Herbarium of the University of Aarhus	Gunnera
Herbarium of the University of Aarhus	Gunnera
Missouri Botanical Garden	Gunnera atropurpurea L.E. Mora
Herbarium of the University of Aarhus	Gunnera magellanica Lam.
Herbarium of the University of Aarhus	Gunnera
GBIF-Sweden	Gunnera brephogea Linden & Andr√©
Missouri Botanical Garden	Gunnera magellanica Lam.
Missouri Botanical Garden	Gunnera L.
Missouri Botanical Garden	Gunnera magellanica Lam.
Missouri Botanical Garden	Gunnera L.
Missouri Botanical Garden	Gunnera L.
Herbarium of the University of Aarhus	Gunnera
Missouri Botanical Garden	Gunnera magellanica Lam.
Missouri Botanical Garden	Gunnera L.
Missouri Botanical Garden	Gunnera L.
Herbarium of the University of Aarhus	Gunnera magellanica Lam.
Missouri Botanical Garden	Gunnera L.

Missouri Botanical Garden	Gunnera pilosa Kunth
Missouri Botanical Garden	Gunnera atropurpurea var. atropurpurea
Missouri Botanical Garden	Gunnera L.
Missouri Botanical Garden	Gunnera magellanica Lam.
Herbarium of the University of Aarhus	Gunnera magellanica Lam.
Herbarium of the University of Aarhus	Gunnera
Missouri Botanical Garden	Gunnera magellanica Lam.
Missouri Botanical Garden	Gunnera magellanica Lam.
Herbarium of the University of Aarhus	Gunnera
Herbarium of the University of Aarhus	Gunnera magellanica Lam.
Herbarium of the University of Aarhus	Gunnera
Missouri Botanical Garden	Gunnera L.
Herbarium of the University of Aarhus	Gunnera
Missouri Botanical Garden	Gunnera perpensa L.
National Herbarium of New South Wales	Gunnera macrophylla
Missouri Botanical Garden	Gunnera L.
Missouri Botanical Garden	Gunnera L.
GBIF-Sweden	Gunnera brephogea Linden & Andr√©
Herbarium of the University of Aarhus	Gunnera
Missouri Botanical Garden	Gunnera L.
GBIF-Sweden	Gunnera brephogea Linden & Andr√©
National Museum of Natural History, Smithsonian Institution	Gunnera magallanica
GBIF-Sweden	Gunnera pilosa Kunth
GBIF-Sweden	Gunnera atropurpurea L. E. Mora
GBIF-Sweden	Gunnera pilosa Kunth
Herbarium of the University of Aarhus	Gunnera
Missouri Botanical Garden	Gunnera L.
Herbarium of the University of Aarhus	Gunnera
Missouri Botanical Garden	Gunnera L.
Missouri Botanical Garden	Gunnera L.
Missouri Botanical Garden	Gunnera L.
Missouri Botanical Garden	Gunnera L.
Missouri Botanical Garden	Gunnera L.
Missouri Botanical Garden	Gunnera magellanica Lam.
Missouri Botanical Garden	Gunnera magellanica Lam.

National Museum of Natural History, Smithsonian Institution	Gunnera magallanica
Missouri Botanical Garden	Gunnera L.
Netherlands Centre for Biodiversity Naturalis, section National Herbarium of the Netherlands	Gunnera magellanica Lam.
Missouri Botanical Garden	Gunnera L.
Herbarium of the University of Aarhus	Gunnera magellanica Lam.
Missouri Botanical Garden	Gunnera magellanica Lam.
Missouri Botanical Garden	Gunnera L.
Missouri Botanical Garden	Gunnera magellanica Lam.
Missouri Botanical Garden	Gunnera L.
Herbarium of the University of Aarhus	Gunnera
Herbarium of the University of Aarhus	Gunnera magellanica Lam.
Herbarium of the University of Aarhus	Gunnera
Herbarium of the University of Aarhus	Gunnera magellanica Lam.
Missouri Botanical Garden	Gunnera magellanica Lam.
Missouri Botanical Garden	Gunnera magellanica Lam.
Herbarium of the University of Aarhus	Gunnera
Herbarium of the University of Aarhus	Gunnera magellanica Lam.
Missouri Botanical Garden	Gunnera magellanica Lam.
Missouri Botanical Garden	Gunnera brephogea Linden & Andr√©
Missouri Botanical Garden	Gunnera magellanica Lam.
Herbarium of the University of Aarhus	Gunnera
Herbarium of the University of Aarhus	Gunnera
Herbarium of the University of Aarhus	Gunnera pilosa Kunth
Missouri Botanical Garden	Gunnera L.
Missouri Botanical Garden	Gunnera L.
Herbarium of the University of Aarhus	Gunnera magellanica Lam.
Herbarium of the University of Aarhus	Gunnera magellanica Lam.
Herbarium of the University of Aarhus	Gunnera magellanica Lam.
Missouri Botanical Garden	Gunnera magellanica Lam.
Missouri Botanical Garden	Gunnera magellanica Lam.
Missouri Botanical Garden	Gunnera magellanica Lam.
Herbarium of the University of Aarhus	Gunnera magellanica Lam.
Missouri Botanical Garden	Gunnera magellanica Lam.
Herbarium of the University of Aarhus	Gunnera magellanica Lam.
Herbarium of the University of Aarhus	Gunnera

Missouri Botanical Garden	Gunnera magellanica Lam.
Missouri Botanical Garden	Gunnera L.
Missouri Botanical Garden	Gunnera magellanica Lam.
Missouri Botanical Garden	Gunnera brephogea Linden & Andr√©
Missouri Botanical Garden	Gunnera magellanica Lam.
Missouri Botanical Garden	Gunnera L.
Missouri Botanical Garden	Gunnera magellanica Lam.
Missouri Botanical Garden	Gunnera L.
Missouri Botanical Garden	Gunnera magellanica Lam.
Missouri Botanical Garden	Gunnera magellanica Lam.
Missouri Botanical Garden	Gunnera pilosa Kunth
Herbarium of the University of Aarhus	Gunnera
Herbarium of the University of Aarhus	Gunnera magellanica Lam.
Missouri Botanical Garden	Gunnera magellanica Lam.
Missouri Botanical Garden	Gunnera magellanica Lam.
Missouri Botanical Garden	Gunnera brephogea Linden & Andr√©
Missouri Botanical Garden	Gunnera magellanica Lam.
Field Museum	Gunnera brephogea Linden & Andr√©
National Herbarium of New South Wales	Gunnera
Australian National Herbarium (CANB)	Gunnera macrophylla
Australian National Herbarium (CANB)	Gunnera
Australian National Herbarium (CANB)	Gunnera
National Herbarium of New South Wales	Gunnera macrophylla
National Herbarium of New South Wales	Gunnera
Missouri Botanical Garden	Gunnera brephogea subsp. magna L.E. Mora
Missouri Botanical Garden	Gunnera L.
Herbarium of the University of Aarhus	Gunnera magellanica Lam.
Missouri Botanical Garden	Gunnera L.
Missouri Botanical Garden	Gunnera L.
Missouri Botanical Garden	Gunnera magellanica Lam.
Missouri Botanical Garden	Gunnera magellanica Lam.
Missouri Botanical Garden	Gunnera brephogea Linden & Andr√©
Missouri Botanical Garden	Gunnera L.
Missouri Botanical Garden	Gunnera magellanica Lam.
Herbarium of the University of Aarhus	Gunnera magellanica Lam.

Missouri Botanical Garden	Gunnera brephogea Linden & Andr√©
Missouri Botanical Garden	Gunnera magellanica Lam.
Missouri Botanical Garden	Gunnera magellanica Lam.
Herbarium of the University of Aarhus	Gunnera magellanica Lam.
Herbarium of the University of Aarhus	Gunnera magellanica Lam.
Missouri Botanical Garden	Gunnera brephogea Linden & Andr√©
Missouri Botanical Garden	Gunnera magellanica Lam.
Missouri Botanical Garden	Gunnera magellanica Lam.
Missouri Botanical Garden	Gunnera magellanica Lam.
Missouri Botanical Garden	Gunnera magellanica Lam.
Herbarium of the University of Aarhus	Gunnera magellanica Lam.
Missouri Botanical Garden	Gunnera magellanica Lam.
Missouri Botanical Garden	Gunnera magellanica Lam.
Herbarium of the University of Aarhus	Gunnera magellanica Lam.
Missouri Botanical Garden	Gunnera magellanica Lam.
Missouri Botanical Garden	Gunnera magellanica Lam.
Missouri Botanical Garden	Gunnera magellanica Lam.
Herbarium of the University of Aarhus	Gunnera
Herbarium of the University of Aarhus	Gunnera magellanica Lam.
Herbarium of the University of Aarhus	Gunnera magellanica Lam.
Herbarium of the University of Aarhus	Gunnera
Herbarium of the University of Aarhus	Gunnera
Herbarium of the University of Aarhus	Gunnera magellanica Lam.
Missouri Botanical Garden	Gunnera magellanica Lam.
Missouri Botanical Garden	Gunnera magellanica Lam.
Herbarium of the University of Aarhus	Gunnera magellanica Lam.
Herbarium of the University of Aarhus	Gunnera magellanica Lam.
Missouri Botanical Garden	Gunnera L.
Herbarium of the University of Aarhus	Gunnera magellanica Lam.
Herbarium of the University of Aarhus	Gunnera magellanica Lam.
Missouri Botanical Garden	Gunnera magellanica Lam.
Herbarium of the University of Aarhus	Gunnera
National Museum of Natural History, Smithsonian Institution	Gunnera magallanica
Missouri Botanical Garden	Gunnera L.
Missouri Botanical Garden	Gunnera magellanica Lam.

Herbarium of the University of Aarhus	Gunnera magellanica Lam.
Missouri Botanical Garden	Gunnera L.
Missouri Botanical Garden	Gunnera magellanica Lam.
Herbarium of the University of Aarhus	Gunnera magellanica Lam.
Missouri Botanical Garden	Gunnera magellanica Lam.
Missouri Botanical Garden	Gunnera L.
Missouri Botanical Garden	Gunnera magellanica Lam.
Missouri Botanical Garden	Gunnera L.
Missouri Botanical Garden	Gunnera magellanica Lam.
Herbarium of the University of Aarhus	Gunnera magellanica Lam.
Herbarium of the University of Aarhus	Gunnera magellanica Lam.
Missouri Botanical Garden	Gunnera L.
Missouri Botanical Garden	Gunnera L.
Missouri Botanical Garden	Gunnera brephogea Linden & Andr√©
Missouri Botanical Garden	Gunnera L.
Missouri Botanical Garden	Gunnera L.
Missouri Botanical Garden	Gunnera L.
GBIF-Sweden	Gunnera brephogea Linden & Andr√©
Missouri Botanical Garden	Gunnera L.
Herbarium of the University of Aarhus	Gunnera
Herbarium of the University of Aarhus	Gunnera
Herbarium of the University of Aarhus	Gunnera
GBIF-Sweden	Gunnera pilosa Kunth
Missouri Botanical Garden	Gunnera L.
University of Connecticut	Gunnera
Herbarium of the University of Aarhus	Gunnera
Missouri Botanical Garden	Gunnera L.
Missouri Botanical Garden	Gunnera brephogea Linden & Andr√©
Missouri Botanical Garden	Gunnera brephogea Linden & Andr√©
Missouri Botanical Garden	Gunnera L.
Herbarium of the University of Aarhus	Gunnera
Missouri Botanical Garden	Gunnera L.
Missouri Botanical Garden	Gunnera L.
Missouri Botanical Garden	Gunnera L.
Natural History Museum, Vienna - Herbarium W	Gunnera ecuadoriana Gilli

Missouri Botanical Garden	Gunnera magellanica Lam.
Missouri Botanical Garden	Gunnera L.
Missouri Botanical Garden	Gunnera brephogea Linden & Andr√©
Herbarium of the University of Aarhus	Gunnera
Missouri Botanical Garden	Gunnera L.
Missouri Botanical Garden	Gunnera L.
Herbarium of the University of Aarhus	Gunnera
Missouri Botanical Garden	Gunnera brephogea Linden & Andr√©
Missouri Botanical Garden	Gunnera magellanica Lam.
Missouri Botanical Garden	Gunnera L.
Missouri Botanical Garden	Gunnera magellanica Lam.
Missouri Botanical Garden	Gunnera L.
Missouri Botanical Garden	Gunnera L.
Missouri Botanical Garden	Gunnera L.
Herbarium of the University of Aarhus	Gunnera magellanica Lam.
Missouri Botanical Garden	Gunnera brephogea Linden & Andr√©
Missouri Botanical Garden	Gunnera L.
Missouri Botanical Garden	Gunnera magellanica Lam.
GBIF-Sweden	Gunnera magellanica Lam.
Missouri Botanical Garden	Gunnera L.
Missouri Botanical Garden	Gunnera magellanica Lam.
GBIF-Sweden	Gunnera magellanica Lam.
Missouri Botanical Garden	Gunnera magellanica Lam.
Missouri Botanical Garden	Gunnera magellanica Lam.
Missouri Botanical Garden	Gunnera magellanica Lam.
Missouri Botanical Garden	Gunnera magellanica Lam.
The New York Botanical Garden	Gunnera quitoensis L. E. Mora
Netherlands Centre for Biodiversity Naturalis, section National Herbarium of the Netherlands	Gunnera magellanica Lam.
Herbarium of the University of Aarhus	Gunnera magellanica Lam.
GBIF-Sweden	Gunnera magellanica Lam.
The New York Botanical Garden	Gunnera brephogea Linden & Andr√©
Missouri Botanical Garden	Gunnera L.
The New York Botanical Garden	Gunnera magellanica Lam.
Herbarium of the University of Aarhus	Gunnera magellanica Lam.
The New York Botanical Garden	Gunnera magellanica Lam.

Missouri Botanical Garden	Gunnera magellanica Lam.
Herbarium of the University of Aarhus	Gunnera magellanica Lam.
GBIF-Sweden	Gunnera magellanica Lam.
Missouri Botanical Garden	Gunnera L.
National Museum of Natural History, Smithsonian Institution	Gunnera pilosa
National Museum of Natural History, Smithsonian Institution	Gunnera pilosa
Missouri Botanical Garden	Gunnera magellanica Lam.
Netherlands Centre for Biodiversity Naturalis, section National Herbarium of the Netherlands	Gunnera magellanica Lam.
Missouri Botanical Garden	Gunnera magellanica Lam.
Missouri Botanical Garden	Gunnera magellanica Lam.
Instituto de Investigaci√≥n de Recursos Biol√≥gicos Alexander von Humboldt	Gunnera colombiana
Missouri Botanical Garden	Gunnera L.
Instituto de Investigaci√≥n de Recursos Biol√≥gicos Alexander von Humboldt	Gunnera
Instituto de Investigaci√≥n de Recursos Biol√≥gicos Alexander von Humboldt	Gunnera
Instituto de Investigaci√≥n de Recursos Biol√≥gicos Alexander von Humboldt	Gunnera
Missouri Botanical Garden	Gunnera L.
Instituto de Ciencias Naturales	Gunnera pilosa
Instituto de Investigaci√≥n de Recursos Biol√≥gicos Alexander von Humboldt	Gunnera pilosa
Instituto de Ciencias Naturales	Gunnera atropurpurea L.E. Mora var. munchicana L.E. Mora
Instituto de Investigaci√≥n de Recursos Biol√≥gicos Alexander von Humboldt	Gunnera pilosa
Instituto de Ciencias Naturales	Gunnera magellanica
Instituto de Ciencias Naturales	Gunnera atropurpurea var. munchicana
Instituto de Investigaci√≥n de Recursos Biol√≥gicos Alexander von Humboldt	Gunnera
Field Museum	Gunnera magnifica H. St. John
Field Museum	Gunnera magnifica H. St. John
Field Museum	Gunnera magnifica H. St. John
Field Museum	Gunnera magnifica H. St. John
Field Museum	Gunnera magnifica H. St. John
Field Museum	Gunnera magnifica H. St. John
Field Museum	Gunnera magnifica H. St. John
National Museum of Natural History, Smithsonian Institution	Gunnera magnifica
Instituto de Ciencias Naturales	Gunnera brephogea
Instituto de Ciencias Naturales	Gunnera brephogea Linden & Andr√©
Instituto de Ciencias Naturales	Gunnera sp.
Instituto de Ciencias Naturales	Gunnera brephogea Linden & Andr√©

National Museum of Natural History, Smithsonian Institution	Gunnera magnifica
Instituto de Ciencias Naturales	Gunnera magellanica
Instituto de Ciencias Naturales	Gunnera sp.
National Museum of Natural History, Smithsonian Institution	Gunnera magnifica
Instituto de Ciencias Naturales	Gunnera brephogea Linden & Andr√©
National Museum of Natural History, Smithsonian Institution	Gunnera magnifica
Instituto de Ciencias Naturales	Gunnera brephogea Linden & Andr√©
Instituto de Ciencias Naturales	Gunnera brephogea
National Museum of Natural History, Smithsonian Institution	Gunnera magnifica
National Museum of Natural History, Smithsonian Institution	Gunnera magnifica
Instituto de Ciencias Naturales	Gunnera brephogea Linden & Andr√©
Instituto de Ciencias Naturales	Gunnera brephogea
National Museum of Natural History, Smithsonian Institution	Gunnera magnifica
National Museum of Natural History, Smithsonian Institution	Gunnera magnifica
National Museum of Natural History, Smithsonian Institution	Gunnera magnifica
National Museum of Natural History, Smithsonian Institution	Gunnera magnifica
Instituto de Ciencias Naturales	Gunnera brephogea
National Museum of Natural History, Smithsonian Institution	Gunnera magnifica
National Museum of Natural History, Smithsonian Institution	Gunnera magnifica
National Museum of Natural History, Smithsonian Institution	Gunnera magnifica
Instituto de Ciencias Naturales	Gunnera sp.
Instituto de Ciencias Naturales	Gunnera brephogea Linden & Andr√©
National Museum of Natural History, Smithsonian Institution	Gunnera magnifica
Instituto de Ciencias Naturales	Gunnera sp.
National Museum of Natural History, Smithsonian Institution	Gunnera magnifica
Instituto de Ciencias Naturales	Gunnera brephogea
National Museum of Natural History, Smithsonian Institution	Gunnera magnifica
Instituto de Ciencias Naturales	Gunnera pilosa
Instituto de Ciencias Naturales	Gunnera brephogea
National Museum of Natural History, Smithsonian Institution	Gunnera magnifica
Instituto de Ciencias Naturales	Gunnera pilosa Kunth
Instituto de Ciencias Naturales	Gunnera sp.
National Museum of Natural History, Smithsonian Institution	Gunnera pilosa
National Museum of Natural History, Smithsonian Institution	Gunnera pilosa
Instituto de Ciencias Naturales	Gunnera bogotana

Instituto de Ciencias Naturales	Gunnera bogotana L.E. Mora
Instituto de Ciencias Naturales	Gunnera bogotana L.E. Mora
Instituto de Ciencias Naturales	Gunnera bogotana L.E. Mora
Instituto de Ciencias Naturales	Gunnera bogotana L.E. Mora
Instituto de Ciencias Naturales	Gunnera bogotana
Instituto de Ciencias Naturales	Gunnera bogotana
Instituto de Ciencias Naturales	Gunnera bogotana
Instituto de Ciencias Naturales	Gunnera bogotana
Instituto de Ciencias Naturales	Gunnera bogotana
Instituto de Ciencias Naturales	Gunnera bogotana
Instituto de Ciencias Naturales	Gunnera bogotana
Instituto de Ciencias Naturales	Gunnera bogotana
Instituto de Ciencias Naturales	Gunnera bogotana
Instituto de Ciencias Naturales	Gunnera bogotana L.E. Mora
Instituto de Ciencias Naturales	Gunnera bogotana
Instituto de Ciencias Naturales	Gunnera bogotana
Instituto de Ciencias Naturales	Gunnera bogotana L.E. Mora
National Museum of Natural History, Smithsonian Institution	Gunnera pilosa
Instituto de Ciencias Naturales	Gunnera bogotana
Instituto de Ciencias Naturales	Gunnera bogotana
Instituto de Ciencias Naturales	Gunnera bogotana
Instituto de Ciencias Naturales	Gunnera bogotana
Instituto de Ciencias Naturales	Gunnera bogotana L.E. Mora
Instituto de Ciencias Naturales	Gunnera bogotana
Instituto de Ciencias Naturales	Gunnera schultesii
National Museum of Natural History, Smithsonian Institution	Gunnera pilosa
Instituto de Ciencias Naturales	Gunnera bogotana L.E. Mora
Instituto de Ciencias Naturales	Gunnera bogotana L.E. Mora
Instituto de Ciencias Naturales	Gunnera bogotana
Instituto de Ciencias Naturales	Gunnera bogotana L.E. Mora
Instituto de Ciencias Naturales	Gunnera bogotana
Instituto de Ciencias Naturales	Gunnera bogotana
Instituto de Ciencias Naturales	Gunnera bogotana
Instituto de Ciencias Naturales	Gunnera bogotana L.E. Mora
National Museum of Natural History, Smithsonian Institution	Gunnera pilosa

Instituto de Ciencias Naturales	Gunnera bogotana
Instituto de Ciencias Naturales	Gunnera bogotana
Instituto de Ciencias Naturales	Gunnera bogotana L.E. Mora
Instituto de Ciencias Naturales	Gunnera bogotana
Instituto de Ciencias Naturales	Gunnera schultesii
Instituto de Ciencias Naturales	Gunnera bogotana L.E. Mora
Instituto de Ciencias Naturales	Gunnera bogotana
Instituto de Ciencias Naturales	Gunnera bogotana
Instituto de Ciencias Naturales	Gunnera bogotana L.E. Mora
Instituto de Ciencias Naturales	Gunnera bogotana
Instituto de Ciencias Naturales	Gunnera bogotana
Instituto de Ciencias Naturales	Gunnera bogotana L.E. Mora
Instituto de Ciencias Naturales	Gunnera bogotana L.E. Mora
Instituto de Ciencias Naturales	Gunnera bogotana L.E. Mora
Instituto de Ciencias Naturales	Gunnera bogotana
Instituto de Ciencias Naturales	Gunnera bogotana
Instituto de Ciencias Naturales	Gunnera bogotana L.E. Mora
Instituto de Ciencias Naturales	Gunnera bogotana L.E. Mora
Instituto de Ciencias Naturales	Gunnera schultesii
Instituto de Ciencias Naturales	Gunnera bogotana
Instituto de Ciencias Naturales	Gunnera bogotana
Instituto de Ciencias Naturales	Gunnera bogotana
Instituto de Ciencias Naturales	Gunnera bogotana L.E. Mora
Instituto de Ciencias Naturales	Gunnera bogotana
Instituto de Ciencias Naturales	Gunnera bogotana L.E. Mora
Instituto de Ciencias Naturales	Gunnera bogotana
Instituto de Ciencias Naturales	Gunnera bogotana
Instituto de Ciencias Naturales	Gunnera bogotana
Instituto de Ciencias Naturales	Gunnera bogotana
Instituto de Ciencias Naturales	Gunnera bogotana L.E. Mora
Instituto de Ciencias Naturales	Gunnera bogotana L.E. Mora
Instituto de Ciencias Naturales	Gunnera bogotana
Instituto de Ciencias Naturales	Gunnera bogotana L.E. Mora
Instituto de Ciencias Naturales	Gunnera bogotana
Instituto de Ciencias Naturales	Gunnera bogotana L.E. Mora

Instituto de Ciencias Naturales	Gunnera bogotana L.E. Mora
Instituto de Ciencias Naturales	Gunnera bogotana
Instituto de Ciencias Naturales	Gunnera bogotana L.E. Mora
Instituto de Ciencias Naturales	Gunnera bogotana L.E. Mora
Instituto de Ciencias Naturales	Gunnera bogotana
Instituto de Ciencias Naturales	Gunnera bogotana
Instituto de Ciencias Naturales	Gunnera bogotana
Instituto de Ciencias Naturales	Gunnera bogotana L.E. Mora
Instituto de Ciencias Naturales	Gunnera bogotana L.E. Mora
Instituto de Ciencias Naturales	Gunnera bogotana
Instituto de Ciencias Naturales	Gunnera bogotana
Instituto de Ciencias Naturales	Gunnera bogotana
Instituto de Ciencias Naturales	Gunnera bogotana
Instituto de Ciencias Naturales	Gunnera bogotana L.E. Mora
Instituto de Ciencias Naturales	Gunnera bogotana L.E. Mora
Instituto de Ciencias Naturales	Gunnera bogotana
Instituto de Ciencias Naturales	Gunnera bogotana L.E. Mora
Instituto de Ciencias Naturales	Gunnera bogotana
Instituto de Ciencias Naturales	Gunnera bogotana
Instituto de Ciencias Naturales	Gunnera bogotana
Instituto de Ciencias Naturales	Gunnera bogotana
Instituto de Ciencias Naturales	Gunnera bogotana
Instituto de Ciencias Naturales	Gunnera schultesii
Instituto de Ciencias Naturales	Gunnera bogotana
Instituto de Ciencias Naturales	Gunnera bogotana
Instituto de Ciencias Naturales	Gunnera bogotana L.E. Mora
Instituto de Ciencias Naturales	Gunnera bogotana
Instituto de Ciencias Naturales	Gunnera bogotana
Instituto de Ciencias Naturales	Gunnera bogotana L.E. Mora
Instituto de Ciencias Naturales	Gunnera bogotana L.E. Mora
Instituto de Ciencias Naturales	Gunnera bogotana
Instituto de Ciencias Naturales	Gunnera bogotana L.E. Mora
Instituto de Ciencias Naturales	Gunnera bogotana L.E. Mora
Instituto de Ciencias Naturales	Gunnera bogotana
Instituto de Ciencias Naturales	Gunnera bogotana L.E. Mora

Instituto de Ciencias Naturales	Gunnera bogotana L.E. Mora
National Museum of Natural History, Smithsonian Institution	Gunnera pilosa
Instituto de Ciencias Naturales	Gunnera bogotana
Instituto de Ciencias Naturales	Gunnera bogotana L.E. Mora
Instituto de Ciencias Naturales	Gunnera magellanica
Instituto de Ciencias Naturales	Gunnera antioquensis
Instituto de Ciencias Naturales	Gunnera antioquensis
Missouri Botanical Garden	Gunnera antioquensis L.E. Mora
Missouri Botanical Garden	Gunnera L.
Instituto de Ciencias Naturales	Gunnera brephogea Linden & Andr√©
Instituto de Ciencias Naturales	Gunnera brephogea
Instituto de Ciencias Naturales	Gunnera sp.
Instituto de Ciencias Naturales	Gunnera sp.
Instituto de Ciencias Naturales	Gunnera pilosa
Instituto de Ciencias Naturales	Gunnera sp.
Instituto de Ciencias Naturales	Gunnera schultesii
Instituto de Ciencias Naturales	Gunnera sp.
Instituto de Ciencias Naturales	Gunnera brephogea Linden & Andr√©
Instituto de Ciencias Naturales	Gunnera pilosa Kunth
Instituto de Ciencias Naturales	Gunnera pilosa
Missouri Botanical Garden	Gunnera L.
Missouri Botanical Garden	Gunnera L.
Missouri Botanical Garden	Gunnera brephogea Linden & Andr√©
Missouri Botanical Garden	Gunnera pilosa Kunth
Missouri Botanical Garden	Gunnera L.
Instituto de Ciencias Naturales	Gunnera pilosa Kunth
Missouri Botanical Garden	Gunnera L.
Instituto de Ciencias Naturales	Gunnera brephogea
Instituto de Ciencias Naturales	Gunnera pilosa
Missouri Botanical Garden	Gunnera L.
Missouri Botanical Garden	Gunnera L.
Missouri Botanical Garden	Gunnera L.
Instituto de Ciencias Naturales	Gunnera lozanii
Instituto de Ciencias Naturales	Gunnera lozanii
Instituto de Ciencias Naturales	Gunnera lozanii

Instituto de Ciencias Naturales	Gunnera lozanii
Instituto de Ciencias Naturales	Gunnera lozanii
Instituto de Ciencias Naturales	Gunnera lozanii
Instituto de Ciencias Naturales	Gunnera pilosa Kunth
Instituto de Ciencias Naturales	Gunnera pilosa
Missouri Botanical Garden	Gunnera insignis (Oerst.) A. DC.
Missouri Botanical Garden	Gunnera L.
Field Museum	Gunnera pittierana V. M. Badillo & Steyerm.
Missouri Botanical Garden	Gunnera talamancana H. Weber & L.E. Mora
Missouri Botanical Garden	Gunnera insignis (Oerst.) A. DC.
Missouri Botanical Garden	Gunnera talamancana H. Weber & L.E. Mora
Missouri Botanical Garden	Gunnera insignis (Oerst.) A. DC.
Missouri Botanical Garden	Gunnera insignis (Oerst.) A. DC.
Missouri Botanical Garden	Gunnera insignis (Oerst.) A. DC.
Missouri Botanical Garden	Gunnera insignis (Oerst.) A. DC.
Field Museum	Gunnera insignis Oerst.
Missouri Botanical Garden	Gunnera insignis (Oerst.) A. DC.
Missouri Botanical Garden	Gunnera insignis (Oerst.) A. DC.
Missouri Botanical Garden	Gunnera insignis (Oerst.) A. DC.
Missouri Botanical Garden	Gunnera talamancana H. Weber & L.E. Mora
Missouri Botanical Garden	Gunnera insignis (Oerst.) A. DC.
Missouri Botanical Garden	Gunnera insignis (Oerst.) A. DC.
Missouri Botanical Garden	Gunnera insignis (Oerst.) A. DC.
Field Museum	Gunnera L.
Field Museum	Gunnera insignis Oerst.
Missouri Botanical Garden	Gunnera insignis (Oerst.) A. DC.
Missouri Botanical Garden	Gunnera insignis (Oerst.) A. DC.
Missouri Botanical Garden	Gunnera insignis (Oerst.) A. DC.
Missouri Botanical Garden	Gunnera insignis (Oerst.) A. DC.
Missouri Botanical Garden	Gunnera L.
Missouri Botanical Garden	Gunnera brephogea Linden & Andr√©
Missouri Botanical Garden	Gunnera L.
GBIF-Sweden	Gunnera venezolana L. E. Mora
Missouri Botanical Garden	Gunnera insignis (Oerst.) A. DC.
Instituto Nacional de Biodiversidad (INBio), Costa Rica	Gunnera talamancana

Instituto Nacional de Biodiversidad (INBio), Costa Rica	Gunnera insignis
Museo Nacional de Costa Rica	Gunnera insignis
Instituto Nacional de Biodiversidad (INBio), Costa Rica	Gunnera insignis
Instituto Nacional de Biodiversidad (INBio), Costa Rica	Gunnera insignis
Missouri Botanical Garden	Gunnera L.
Museo Nacional de Costa Rica	Gunnera talamancana
Instituto Nacional de Biodiversidad (INBio), Costa Rica	Gunnera insignis
Missouri Botanical Garden	Gunnera talamancana H. Weber & L.E. Mora
Instituto Nacional de Biodiversidad (INBio), Costa Rica	Gunnera insignis
Museo Nacional de Costa Rica	Gunnera insignis
Netherlands Centre for Biodiversity Naturalis, section National Herbarium of the Netherlands	Gunnera talamancana H.Weber & L.E.Mora
Instituto Nacional de Biodiversidad (INBio), Costa Rica	Gunnera insignis
Missouri Botanical Garden	Gunnera insignis (Oerst.) A. DC.
Missouri Botanical Garden	Gunnera L.
Instituto Nacional de Biodiversidad (INBio), Costa Rica	Gunnera talamancana
Museo Nacional de Costa Rica	Gunnera insignis
Missouri Botanical Garden	Gunnera insignis (Oerst.) A. DC.
Instituto Nacional de Biodiversidad (INBio), Costa Rica	Gunnera insignis
Instituto Nacional de Biodiversidad (INBio), Costa Rica	Gunnera insignis
Instituto Nacional de Biodiversidad (INBio), Costa Rica	Gunnera talamancana
Instituto Nacional de Biodiversidad (INBio), Costa Rica	Gunnera talamancana
Instituto Nacional de Biodiversidad (INBio), Costa Rica	Gunnera talamancana
Missouri Botanical Garden	Gunnera talamancana H. Weber & L.E. Mora
Museo Nacional de Costa Rica	Gunnera talamancana
Instituto Nacional de Biodiversidad (INBio), Costa Rica	Gunnera talamancana
Museo Nacional de Costa Rica	Gunnera talamancana
Instituto Nacional de Biodiversidad (INBio), Costa Rica	Gunnera insignis
Missouri Botanical Garden	Gunnera talamancana H. Weber & L.E. Mora
Instituto Nacional de Biodiversidad (INBio), Costa Rica	Gunnera insignis
Museo Nacional de Costa Rica	Gunnera talamancana
Instituto Nacional de Biodiversidad (INBio), Costa Rica	Gunnera talamancana
Instituto Nacional de Biodiversidad (INBio), Costa Rica	Gunnera talamancana
University of Connecticut	Gunnera talamancana H. Weber & L. E. Mora
Instituto Nacional de Biodiversidad (INBio), Costa Rica	Gunnera talamancana
Missouri Botanical Garden	Gunnera talamancana H. Weber & L.E. Mora

Missouri Botanical Garden	Gunnera insignis (Oerst.) A. DC.
Missouri Botanical Garden	Gunnera talamancana H. Weber & L.E. Mora
Instituto Nacional de Biodiversidad (INBio), Costa Rica	Gunnera insignis
Instituto Nacional de Biodiversidad (INBio), Costa Rica	Gunnera insignis
Instituto Nacional de Biodiversidad (INBio), Costa Rica	Gunnera talamancana
Missouri Botanical Garden	Gunnera insignis (Oerst.) A. DC.
Missouri Botanical Garden	Gunnera talamancana H. Weber & L.E. Mora
Instituto Nacional de Biodiversidad (INBio), Costa Rica	Gunnera talamancana
Missouri Botanical Garden	Gunnera insignis (Oerst.) A. DC.
Missouri Botanical Garden	Gunnera talamancana H. Weber & L.E. Mora
Missouri Botanical Garden	Gunnera insignis (Oerst.) A. DC.
Instituto Nacional de Biodiversidad (INBio), Costa Rica	Gunnera talamancana
Instituto Nacional de Biodiversidad (INBio), Costa Rica	Gunnera talamancana
Instituto Nacional de Biodiversidad (INBio), Costa Rica	Gunnera talamancana
Museo Nacional de Costa Rica	Gunnera insignis
Netherlands Centre for Biodiversity Naturalis, section National Herbarium of the Netherlands	Gunnera talamancana H.Weber & L.E.Mora
Instituto Nacional de Biodiversidad (INBio), Costa Rica	Gunnera talamancana
Field Museum	Gunnera talamancana H. Weber & L. E. Mora
Missouri Botanical Garden	Gunnera insignis (Oerst.) A. DC.
Missouri Botanical Garden	Gunnera talamancana H. Weber & L.E. Mora
Missouri Botanical Garden	Gunnera insignis (Oerst.) A. DC.
Missouri Botanical Garden	Gunnera insignis (Oerst.) A. DC.
Instituto Nacional de Biodiversidad (INBio), Costa Rica	Gunnera talamancana
Museo Nacional de Costa Rica	Gunnera talamancana
Missouri Botanical Garden	Gunnera insignis (Oerst.) A. DC.
Missouri Botanical Garden	Gunnera talamancana H. Weber & L.E. Mora
Instituto Nacional de Biodiversidad (INBio), Costa Rica	Gunnera insignis
Instituto Nacional de Biodiversidad (INBio), Costa Rica	Gunnera talamancana
Instituto Nacional de Biodiversidad (INBio), Costa Rica	Gunnera insignis
Instituto Nacional de Biodiversidad (INBio), Costa Rica	Gunnera insignis
Instituto Nacional de Biodiversidad (INBio), Costa Rica	Gunnera insignis
Instituto Nacional de Biodiversidad (INBio), Costa Rica	Gunnera talamancana
Museo Nacional de Costa Rica	Gunnera insignis
Instituto Nacional de Biodiversidad (INBio), Costa Rica	Gunnera insignis
Museo Nacional de Costa Rica	Gunnera talamancana

Instituto Nacional de Biodiversidad (INBio), Costa Rica	Gunnera
Museo Nacional de Costa Rica	Gunnera insignis
Museo Nacional de Costa Rica	Gunnera talamancana
Museo Nacional de Costa Rica	Gunnera insignis
Instituto Nacional de Biodiversidad (INBio), Costa Rica	Gunnera talamancana
Missouri Botanical Garden	Gunnera talamancana H. Weber & L.E. Mora
Missouri Botanical Garden	Gunnera talamancana H. Weber & L.E. Mora
Instituto Nacional de Biodiversidad (INBio), Costa Rica	Gunnera insignis
Instituto Nacional de Biodiversidad (INBio), Costa Rica	Gunnera insignis
Missouri Botanical Garden	Gunnera x katherine-wilsoniae L.D. G√≥mez
Missouri Botanical Garden	Gunnera insignis (Oerst.) A. DC.
Missouri Botanical Garden	Gunnera talamancana H. Weber & L.E. Mora
Missouri Botanical Garden	Gunnera insignis (Oerst.) A. DC.
Missouri Botanical Garden	Gunnera insignis (Oerst.) A. DC.
Missouri Botanical Garden	Gunnera talamancana H. Weber & L.E. Mora
Instituto Nacional de Biodiversidad (INBio), Costa Rica	Gunnera insignis
Instituto Nacional de Biodiversidad (INBio), Costa Rica	Gunnera insignis
Missouri Botanical Garden	Gunnera insignis (Oerst.) A. DC.
Instituto Nacional de Biodiversidad (INBio), Costa Rica	Gunnera insignis
Instituto Nacional de Biodiversidad (INBio), Costa Rica	Gunnera insignis
Missouri Botanical Garden	Gunnera insignis (Oerst.) A. DC.
Missouri Botanical Garden	Gunnera talamancana H. Weber & L.E. Mora
Instituto Nacional de Biodiversidad (INBio), Costa Rica	Gunnera talamancana
Missouri Botanical Garden	Gunnera L.
Missouri Botanical Garden	Gunnera talamancana H. Weber & L.E. Mora
Museo Nacional de Costa Rica	Gunnera talamancana
Museo Nacional de Costa Rica	Gunnera talamancana
Instituto Nacional de Biodiversidad (INBio), Costa Rica	Gunnera talamancana
Missouri Botanical Garden	Gunnera insignis (Oerst.) A. DC.
Museo Nacional de Costa Rica	Gunnera insignis
Missouri Botanical Garden	Gunnera talamancana H. Weber & L.E. Mora
Instituto Nacional de Biodiversidad (INBio), Costa Rica	Gunnera talamancana
Instituto Nacional de Biodiversidad (INBio), Costa Rica	Gunnera talamancana
Missouri Botanical Garden	Gunnera talamancana H. Weber & L.E. Mora
Missouri Botanical Garden	Gunnera talamancana H. Weber & L.E. Mora

Missouri Botanical Garden	Gunnera talamancana H. Weber & L.E. Mora
Instituto Nacional de Biodiversidad (INBio), Costa Rica	Gunnera insignis
National Museum of Natural History, Smithsonian Institution	Gunnera venezolana subsp. venezolana
National Museum of Natural History, Smithsonian Institution	Gunnera venezolana subsp. venezolana
Missouri Botanical Garden	Gunnera L.
GBIF-Sweden	Gunnera venezolana L. E. Mora
GBIF-Sweden	Gunnera venezolana L. E. Mora
National Museum of Natural History, Smithsonian Institution	Gunnera perpensa
Netherlands Centre for Biodiversity Naturalis, section National Herbarium of the Netherlands	Gunnera perpensa L.
Netherlands Centre for Biodiversity Naturalis, section National Herbarium of the Netherlands	Gunnera perpensa L.
Netherlands Centre for Biodiversity Naturalis, section National Herbarium of the Netherlands	Gunnera perpensa L.
Netherlands Centre for Biodiversity Naturalis, section National Herbarium of the Netherlands	Gunnera perpensa L.
Netherlands Centre for Biodiversity Naturalis, section National Herbarium of the Netherlands	Gunnera perpensa L.
Netherlands Centre for Biodiversity Naturalis, section National Herbarium of the Netherlands	Gunnera perpensa L.
Netherlands Centre for Biodiversity Naturalis, section National Herbarium of the Netherlands	Gunnera perpensa L.
Netherlands Centre for Biodiversity Naturalis, section National Herbarium of the Netherlands	Gunnera perpensa L.
Netherlands Centre for Biodiversity Naturalis, section National Herbarium of the Netherlands	Gunnera perpensa L.
Netherlands Centre for Biodiversity Naturalis, section National Herbarium of the Netherlands	Gunnera perpensa L.
Instituto Nacional de Biodiversidad (INBio), Costa Rica	Gunnera insignis
Missouri Botanical Garden	Gunnera insignis (Oerst.) A. DC.
Instituto Nacional de Biodiversidad (INBio), Costa Rica	Gunnera insignis
Instituto Nacional de Biodiversidad (INBio), Costa Rica	Gunnera insignis
Instituto Nacional de Biodiversidad (INBio), Costa Rica	Gunnera insignis
Missouri Botanical Garden	Gunnera insignis (Oerst.) A. DC.
Instituto Nacional de Biodiversidad (INBio), Costa Rica	Gunnera insignis
Instituto Nacional de Biodiversidad (INBio), Costa Rica	Gunnera insignis
Missouri Botanical Garden	Gunnera insignis (Oerst.) A. DC.
Missouri Botanical Garden	Gunnera insignis (Oerst.) A. DC.
Museo Nacional de Costa Rica	Gunnera insignis
Instituto Nacional de Biodiversidad (INBio), Costa Rica	Gunnera insignis
Museo Nacional de Costa Rica	Gunnera insignis
Instituto Nacional de Biodiversidad (INBio), Costa Rica	Gunnera insignis
Instituto Nacional de Biodiversidad (INBio), Costa Rica	Gunnera insignis
Instituto Nacional de Biodiversidad (INBio), Costa Rica	Gunnera insignis
Instituto Nacional de Biodiversidad (INBio), Costa Rica	Gunnera insignis

Missouri Botanical Garden	Gunnera L.
Instituto Nacional de Biodiversidad (INBio), Costa Rica	Gunnera
Instituto Nacional de Biodiversidad (INBio), Costa Rica	Gunnera insignis
Missouri Botanical Garden	Gunnera insignis (Oerst.) A. DC.
Missouri Botanical Garden	Gunnera insignis (Oerst.) A. DC.
Missouri Botanical Garden	Gunnera insignis (Oerst.) A. DC.
Missouri Botanical Garden	Gunnera insignis (Oerst.) A. DC.
Instituto Nacional de Biodiversidad (INBio), Costa Rica	Gunnera insignis
Museo Nacional de Costa Rica	Gunnera talamancana
Instituto Nacional de Biodiversidad (INBio), Costa Rica	Gunnera
Missouri Botanical Garden	Gunnera insignis (Oerst.) A. DC.
Instituto Nacional de Biodiversidad (INBio), Costa Rica	Gunnera insignis
Missouri Botanical Garden	Gunnera insignis (Oerst.) A. DC.
Missouri Botanical Garden	Gunnera insignis (Oerst.) A. DC.
Instituto Nacional de Biodiversidad (INBio), Costa Rica	Gunnera insignis
National Museum of Natural History, Smithsonian Institution	Gunnera insignis
National Museum of Natural History, Smithsonian Institution	Gunnera insignis
Missouri Botanical Garden	Gunnera insignis (Oerst.) A. DC.
Field Museum	Gunnera insignis f. albovariegata L. D. G√≥mez
Museo Nacional de Costa Rica	Gunnera insignis
Missouri Botanical Garden	Gunnera L.
National Museum of Natural History, Smithsonian Institution	Gunnera sp.
Missouri Botanical Garden	Gunnera tayrona L.E. Mora
Missouri Botanical Garden	Gunnera insignis (Oerst.) A. DC.
Missouri Botanical Garden	Gunnera insignis (Oerst.) A. DC.
Missouri Botanical Garden	Gunnera insignis (Oerst.) A. DC.
Missouri Botanical Garden	Gunnera insignis (Oerst.) A. DC.
Instituto de Ciencias Naturales	Gunnera tayrona
Instituto de Ciencias Naturales	Gunnera tayrona
Instituto de Ciencias Naturales	Gunnera tayrona
Instituto de Ciencias Naturales	Gunnera tayrona
Instituto de Ciencias Naturales	Gunnera tayrona
Instituto de Ciencias Naturales	Gunnera tayrona
Instituto de Ciencias Naturales	Gunnera tayrona
Instituto de Ciencias Naturales	Gunnera tayrona

Instituto de Ciencias Naturales	Gunnera tayrona
Instituto de Ciencias Naturales	Gunnera tayrona
Instituto de Ciencias Naturales	Gunnera tayrona
Instituto de Ciencias Naturales	Gunnera tayrona
Instituto de Ciencias Naturales	Gunnera tayrona
Instituto de Ciencias Naturales	Gunnera tayrona
Instituto de Ciencias Naturales	Gunnera tayrona
Instituto de Ciencias Naturales	Gunnera tayrona
Instituto de Ciencias Naturales	Gunnera tayrona
Instituto de Ciencias Naturales	Gunnera tayrona
Instituto de Ciencias Naturales	Gunnera tayrona
Instituto de Ciencias Naturales	Gunnera tayrona
Instituto de Ciencias Naturales	Gunnera tayrona
Instituto de Ciencias Naturales	Gunnera tayrona
Instituto de Ciencias Naturales	Gunnera tayrona
Instituto de Ciencias Naturales	Gunnera tayrona
Instituto de Ciencias Naturales	Gunnera tayrona
Instituto de Ciencias Naturales	Gunnera tayrona
Instituto de Ciencias Naturales	Gunnera tayrona
Instituto de Ciencias Naturales	Gunnera tayrona
Instituto de Ciencias Naturales	Gunnera tayrona
Instituto de Ciencias Naturales	Gunnera tayrona
Instituto de Ciencias Naturales	Gunnera tayrona
Instituto de Ciencias Naturales	Gunnera tayrona
Missouri Botanical Garden	Gunnera killipiana Lundell
Field Museum	Gunnera killipiana Lundell
Missouri Botanical Garden	Gunnera killipiana Lundell
Field Museum	Gunnera killipiana Lundell
Missouri Botanical Garden	Gunnera killipiana Lundell
Missouri Botanical Garden	Gunnera mexicana Brandegee
Comisi√≥n nacional para el conocimiento y uso de la biodiversidad	Gunnera mexicana Brandegee
Missouri Botanical Garden	Gunnera mexicana Brandegee
Missouri Botanical Garden	Gunnera L.
Missouri Botanical Garden	Gunnera mexicana Brandegee
Missouri Botanical Garden	Gunnera mexicana Brandegee

Field Museum	Gunnera killipiana Lundell
Missouri Botanical Garden	Gunnera mexicana Brandegee
Australian National Herbarium (CANB)	Gunnera macrophylla
Missouri Botanical Garden	Gunnera mexicana Brandegee
University of Connecticut	Gunnera killipiana Lundell
Missouri Botanical Garden	Gunnera mexicana Brandegee
Comisi√≥n nacional para el conocimiento y uso de la biodiversidad	Gunnera insignis (Oerst.) A. DC.
Missouri Botanical Garden	Gunnera mexicana Brandegee
Comisi√≥n nacional para el conocimiento y uso de la biodiversidad	Gunnera killipiana Lundell
Comisi√≥n nacional para el conocimiento y uso de la biodiversidad	Gunnera mexicana Brandegee
USDA PLANTS	Gunnera petaloidea
Comisi√≥n nacional para el conocimiento y uso de la biodiversidad	Gunnera mexicana Brandegee
Comisi√≥n nacional para el conocimiento y uso de la biodiversidad	Gunnera mexicana Brandegee
Comisi√≥n nacional para el conocimiento y uso de la biodiversidad	Gunnera mexicana Brandegee
Missouri Botanical Garden	Gunnera mexicana Brandegee
Comisi√≥n nacional para el conocimiento y uso de la biodiversidad	Gunnera mexicana Brandegee
Comisi√≥n nacional para el conocimiento y uso de la biodiversidad	Gunnera mexicana Brandegee
Comisi√≥n nacional para el conocimiento y uso de la biodiversidad	Gunnera mexicana Brandegee
Comisi√≥n nacional para el conocimiento y uso de la biodiversidad	Gunnera mexicana Brandegee
Comisi√≥n nacional para el conocimiento y uso de la biodiversidad	Gunnera sp. ND
Comisi√≥n nacional para el conocimiento y uso de la biodiversidad	Gunnera mexicana Brandegee
Comisi√≥n nacional para el conocimiento y uso de la biodiversidad	Gunnera mexicana Brandegee
Comisi√≥n nacional para el conocimiento y uso de la biodiversidad	Gunnera mexicana Brandegee
Comisi√≥n nacional para el conocimiento y uso de la biodiversidad	Gunnera mexicana Brandegee
Missouri Botanical Garden	Gunnera mexicana Brandegee
Comisi√≥n nacional para el conocimiento y uso de la biodiversidad	Gunnera mexicana Brandegee
Comisi√≥n nacional para el conocimiento y uso de la biodiversidad	Gunnera L.
Comisi√≥n nacional para el conocimiento y uso de la biodiversidad	Gunnera mexicana Brandegee
Comisi√≥n nacional para el conocimiento y uso de la biodiversidad	Gunnera mexicana Brandegee
Bernice Pauahi Bishop Museum	Gunnera petaloidea
Bernice Pauahi Bishop Museum	Gunnera petaloidea
Bernice Pauahi Bishop Museum	Gunnera petaloidea
USDA PLANTS	Gunnera petaloidea
Bernice Pauahi Bishop Museum	Gunnera petaloidea
Bernice Pauahi Bishop Museum	Gunnera petaloidea

Bernice Pauahi Bishop Museum	Gunnera petaloidea
Bernice Pauahi Bishop Museum	Gunnera petaloidea
Bernice Pauahi Bishop Museum	Gunnera petaloidea
Bernice Pauahi Bishop Museum	Gunnera petaloidea
Bernice Pauahi Bishop Museum	Gunnera petaloidea
Bernice Pauahi Bishop Museum	Gunnera petaloidea
Bernice Pauahi Bishop Museum	Gunnera petaloidea
Bernice Pauahi Bishop Museum	Gunnera petaloidea
Bernice Pauahi Bishop Museum	Gunnera petaloidea
Bernice Pauahi Bishop Museum	Gunnera petaloidea
University of British Columbia	Gunnera petaloides Gaud.
University of British Columbia	Gunnera petaloidea Gaud.
USDA PLANTS	Gunnera kauaiensis
GBIF-Sweden	Gunnera perpensa L.
USDA PLANTS	Gunnera petaloidea
Field Museum	Gunnera killipiana Lundell
The New York Botanical Garden	Gunnera manicata Linden
UK National Biodiversity Network	Gunnera
UK National Biodiversity Network	Gunnera manicata
UK National Biodiversity Network	Gunnera manicata
UK National Biodiversity Network	Gunnera manicata
UK National Biodiversity Network	Gunnera
UK National Biodiversity Network	Gunnera
UK National Biodiversity Network	Gunnera manicata
UK National Biodiversity Network	Gunnera
UK National Biodiversity Network	Gunnera
UK National Biodiversity Network	Gunnera manicata
UK National Biodiversity Network	Gunnera
UK National Biodiversity Network	Gunnera
UK National Biodiversity Network	Gunnera
UK National Biodiversity Network	Gunnera manicata
UK National Biodiversity Network	Gunnera manicata
UK National Biodiversity Network	Gunnera
UK National Biodiversity Network	Gunnera manicata
UK National Biodiversity Network	Gunnera manicata

UK National Biodiversity Network	Gunnera manicata
UK National Biodiversity Network	Gunnera manicata
UK National Biodiversity Network	Gunnera manicata
UK National Biodiversity Network	Gunnera manicata
UK National Biodiversity Network	Gunnera manicata
UK National Biodiversity Network	Gunnera manicata
UK National Biodiversity Network	Gunnera manicata
UK National Biodiversity Network	Gunnera manicata
UK National Biodiversity Network	Gunnera manicata
UK National Biodiversity Network	Gunnera manicata
UK National Biodiversity Network	Gunnera manicata
UK National Biodiversity Network	Gunnera manicata
UK National Biodiversity Network	Gunnera
UK National Biodiversity Network	Gunnera manicata
UK National Biodiversity Network	Gunnera manicata
UK National Biodiversity Network	Gunnera manicata
UK National Biodiversity Network	Gunnera manicata
UK National Biodiversity Network	Gunnera
UK National Biodiversity Network	Gunnera
UK National Biodiversity Network	Gunnera manicata
UK National Biodiversity Network	Gunnera manicata
UK National Biodiversity Network	Gunnera
UK National Biodiversity Network	Gunnera manicata
UK National Biodiversity Network	Gunnera manicata
UK National Biodiversity Network	Gunnera manicata
UK National Biodiversity Network	Gunnera manicata
UK National Biodiversity Network	Gunnera manicata
UK National Biodiversity Network	Gunnera
UK National Biodiversity Network	Gunnera manicata
UK National Biodiversity Network	Gunnera manicata
UK National Biodiversity Network	Gunnera
UK National Biodiversity Network	Gunnera
UK National Biodiversity Network	Gunnera manicata
UK National Biodiversity Network	Gunnera
UK National Biodiversity Network	Gunnera manicata

UK National Biodiversity Network	Gunnera manicata
UK National Biodiversity Network	Gunnera manicata
UK National Biodiversity Network	Gunnera
UK National Biodiversity Network	Gunnera manicata
UK National Biodiversity Network	Gunnera
UK National Biodiversity Network	Gunnera manicata
UK National Biodiversity Network	Gunnera manicata
UK National Biodiversity Network	Gunnera manicata
UK National Biodiversity Network	Gunnera manicata
UK National Biodiversity Network	Gunnera manicata
National Biodiversity Data Centre	Gunnera manicata
National Biodiversity Data Centre	Gunnera manicata
National Biodiversity Data Centre	Gunnera manicata
National Biodiversity Data Centre	Gunnera manicata
National Biodiversity Data Centre	Gunnera manicata
National Biodiversity Data Centre	Gunnera manicata
UK National Biodiversity Network	Gunnera manicata
UK National Biodiversity Network	Gunnera manicata
UK National Biodiversity Network	Gunnera manicata
UK National Biodiversity Network	Gunnera
UK National Biodiversity Network	Gunnera manicata
UK National Biodiversity Network	Gunnera manicata
UK National Biodiversity Network	Gunnera
UK National Biodiversity Network	Gunnera manicata
UK National Biodiversity Network	Gunnera manicata
UK National Biodiversity Network	Gunnera manicata
UK National Biodiversity Network	Gunnera manicata
UK National Biodiversity Network	Gunnera
UK National Biodiversity Network	Gunnera manicata
UK National Biodiversity Network	Gunnera manicata
National Biodiversity Data Centre	Gunnera manicata
UK National Biodiversity Network	Gunnera manicata
UK National Biodiversity Network	Gunnera manicata
UK National Biodiversity Network	Gunnera
UK National Biodiversity Network	Gunnera

UK National Biodiversity Network	Gunnera manicata
UK National Biodiversity Network	Gunnera manicata
UK National Biodiversity Network	Gunnera manicata
UK National Biodiversity Network	Gunnera manicata
UK National Biodiversity Network	Gunnera manicata
UK National Biodiversity Network	Gunnera manicata
ArtDatabanken	Gunnera magellanica
GBIF-Sweden	Gunnera magellanica
ArtDatabanken	Gunnera magellanica
ArtDatabanken	Gunnera magellanica
GBIF-Sweden	Gunnera magellanica
ArtDatabanken	Gunnera magellanica
Finnish Museum of Natural History	Gunnera manicata
Herbarium of the University of Aarhus	Gunnera magellanica Lam.
CONC	
CONC	
CONC	
CONC	
CONC	
CONC	
CONC	
CONC	
CONC	
CONC	
CONC	
CONC	
CONC	
CONC	
CONC	

Scientific name (interpreted)
Gunnera morae Wanntorp & Klack.
Gunnera perpensa L.
Gunnera bracteata Steud. ex Benn.
Gunnera perpensa
Gunnera petaloidea
Gunnera manicata Linden
Gunnera petaloidea
Gunnera macrophylla
Gunnera magellanica Lam.
Gunnera
Gunnera perpensa
Gunnera L.
Gunnera perpensa
Gunnera monoica
Gunnera steyermarkii
Gunnera perpensa
Gunnera magellanica Lam.
Gunnera pilosa
Gunnera lobata Hook.f.
Gunnera manicata
Gunnera herteri
Gunnera scabra
Gunnera perpensa L.
Gunnera monoica
Gunnera herteri
Gunnera dentata Kirk
Gunnera hamiltoni
Gunnera bracteata Steud. ex Benn.
Gunnera magellanica
Gunnera petaloidea var. kauaense
Gunnera perpensa
Gunnera mexicana

Gunnera petaloidea
Gunnera perpensa
Gunnera magellanica
Gunnera manicata Linden
Gunnera lobata J.D. Hook.
Gunnera perpensa
Gunnera petaloidea
Gunnera perpensa var. kilimandscharica Schindl.
Gunnera pilosa
Gunnera magellanica
Gunnera kauaiensis
Gunnera manicata
Gunnera lobata
Gunnera magellanica
Gunnera talamancana
Gunnera petaloidea
Gunnera lobata Hook.
Gunnera talamancana
Gunnera perpensa
Gunnera perpensa
Gunnera colombiana L. E. Mora
Gunnera perpensa
Gunnera manicata
Gunnera insignis
Gunnera mixta Kirk
Gunnera manicata Linden
Gunnera mixta Kirk
Gunnera pilosa
Gunnera manicata Linden
Gunnera cordifolia (Hook.f.) Hook.f.
Gunnera bolivari J. F. Macbr.
Gunnera bracteata Steud. ex J. Benn.
Gunnera densiflora Hook.f.
Gunnera herteri Osten
Gunnera perpensa

Gunnera lobata
Gunnera peltata
Gunnera petaloidea
Gunnera colombiana L.E. Mora
Gunnera bracteata Steud. ex Benn.
Gunnera annae
Gunnera macrophylla Blume
Gunnera petaloidea Gaudich.
Gunnera peltata Phil.
Gunnera monoica
Gunnera monoica
Gunnera mixta Kirk
Gunnera magellanica Lam.
Gunnera magellanica
Gunnera magellanica
Gunnera manicata
Gunnera insignis
Gunnera tajumbina
Gunnera perpensa
Gunnera petaloidea subsp. petaloidea
Gunnera magellanica
Gunnera bracteata
Gunnera petaloidea
Gunnera perpensa
Gunnera perpensa
Gunnera magellanica
Gunnera petaloidea Gaud.
Gunnera petaloidea
Gunnera rheifolia
Gunnera brephogea Linden & Andr√©
Gunnera densiflora Hook.f.
Gunnera petaloidea
Gunnera chilensis Lam.
Gunnera petaloidea Gaudich.
Gunnera perpensa

Gunnera macrophylla
Gunnera pilosa
Gunnera antioquensis
Gunnera steyermarkii L. E. Mora
Gunnera masafuerae Skottsb.
Gunnera petaloidea Gaudich.
Gunnera pilosa
Gunnera magnifica
Gunnera magellanica Lam.
Gunnera pilosa Kunth
Gunnera magellanica Lam.
Gunnera petaloidea
Gunnera magellanica Lam.
Gunnera magellanica
Gunnera brephogea
Gunnera magellanica Lam.
Gunnera petaloidea
Gunnera
Gunnera manicata
Gunnera morae Wanntorp & Klack.
Gunnera insignis (Oerst.) A. DC.
Gunnera magnifica H. St. John
Gunnera perpensa
Gunnera bracteata Steudel ex Bennett
Gunnera perpensa
Gunnera macrophylla
Gunnera pilosa
Gunnera magellanica
Gunnera petaloidea
Gunnera albocarpa
Gunnera chilensis Lam.
Gunnera atropurpurea
Gunnera pilosa
Gunnera atropurpurea var. munchicana L. E. Mora
Gunnera perpensa

Gunnera chilensis Lam.
Gunnera margaretae Schindler
Gunnera killipania
Gunnera brephogea
Gunnera chilensis
Gunnera petaloidea
Gunnera
Gunnera
Gunnera prorepens
Gunnera prorepens Hook.f.
Gunnera peltata Phil.
Gunnera cordifolia (Hook.f.) Hook.f.
Gunnera magellanica Lam.
Gunnera magellanica Lam.
Gunnera herteri Osten
Gunnera boliviana Morong
Gunnera brephogea Linden & Andr√©
Gunnera peltata Phil.
Gunnera perpensa
Gunnera macrophylla
Gunnera mexicana Brandegee
Gunnera brephogea
Gunnera petaloidea
Gunnera pilosa
Gunnera magellanica Lam.
Gunnera manicata
Gunnera monoica
Gunnera mexicana Brandegee
Gunnera talamancana
Gunnera annae Schindl.
Gunnera magellanica Lam.
Gunnera perpensa
Gunnera petaloidea
Gunnera herteri Osten
Gunnera magellanica Lam.

Gunnera bolivari J. F. Macbr.
Gunnera petaloidea
Gunnera annae Schindl.
Gunnera scabra
Gunnera magellanica Lam.
Gunnera manicata Linden
Gunnera magellanica
Gunnera perpensa
Gunnera lobata
Gunnera perpensa
Gunnera perpensa
Gunnera perpensa
Gunnera petaloidea
Gunnera mexicana Brandegee
Gunnera chilensis
Gunnera pilosa
Gunnera macrophylla Blume
Gunnera macrophylla
Gunnera berteroi Phil.
Gunnera killipiana Lundell
Gunnera hamiltonii Kirk
Gunnera insignis (Oerst.) Oerst.
Gunnera manicata Linden
Gunnera manicata Linden
Gunnera brephogea
Gunnera magellanica
Gunnera perpensa L.
Gunnera petaloidea
Gunnera petaloidea
Gunnera hamiltonii Kirk
Gunnera herteri Osten
Gunnera monoica Raoul
Gunnera hernandezii
Gunnera perpensa
Gunnera brephogea

Gunnera perpensa
Gunnera magnifica
Gunnera mexicana Brandegee
Gunnera berteroi Phil.
Gunnera petaloidea
Gunnera manicata Linden
Gunnera manicata Linden
Gunnera petaloidea
Gunnera perpensa L.
Gunnera prorepens
Gunnera monoica Raoul
Gunnera perpensa L.
Gunnera strigosa (Kirk) Colenso
Gunnera steyermarkii
Gunnera magellanica Lam.
Gunnera scabra Ruiz & Pav.
Gunnera colombiana L. E. Mora
Gunnera herteri Osten
Gunnera masafuerae Skottsb.
Gunnera macrophylla
Gunnera magallanica
Gunnera perpensa
Gunnera perpensa
Gunnera wendlandii Reinke ex Schindl.
Gunnera bracteata Steud. ex Benn.
Gunnera magellanica Lam.
Gunnera dentata Kirk
Gunnera lobata
Gunnera killipiana
Gunnera
Gunnera insignis
Gunnera bracteata
Gunnera magellanica Lam.
Gunnera pilosa Kunth
Gunnera berteroi Phil.

Gunnera perpensa
Gunnera perpensa
Gunnera brephogea
Gunnera magellanica
Gunnera macrophylla
Gunnera petaloidea
Gunnera perpensa
Gunnera
Gunnera pilosa
Gunnera brephogea Linden & Andr√©
Gunnera annae Schindl.
Gunnera magellanica
Gunnera monoica
Gunnera perpensa
Gunnera perpensa
Gunnera perpensa
Gunnera masafuerae Skottsb.
Gunnera brephogea Linden & Andr√©
Gunnera manicata Linden ex Andr√©
Gunnera morae Wanntorp & Klack.
Gunnera densiflora Hook.f.
Gunnera atropurpurea
Gunnera petaloidea
Gunnera bracteata Steud. ex Benn.
Gunnera petaloidea
Gunnera killipania
Gunnera albocarpa
Gunnera perpensa
Gunnera insignis (Oerst.) A. DC.
Gunnera herteri Osten
Gunnera monoica Raoul
Gunnera perpensa L.
Gunnera manicata Linden ex Delchev.
Gunnera herteri Ost.
Gunnera magellanica

Gunnera monoica
Gunnera perpensa
Gunnera magellanica
Gunnera margaretae Schindler
Gunnera perpensa
Gunnera colombiana L. E. Mora
Gunnera petaloidea Gaudich.
Gunnera magellanica
Gunnera kauaiensis Rock
Gunnera dentata Kirk
Gunnera pilosa
Gunnera pilosa
Gunnera pilosa
Gunnera insignis
Gunnera magellanica Lam.
Gunnera monoica Raoul
Gunnera
Gunnera colombiana L. E. Mora
Gunnera prorepens Hook.
Gunnera
Gunnera insignis
Gunnera petaloidea
Gunnera apiculata
Gunnera manicata
Gunnera magellanica Lam.
Gunnera monoica
Gunnera manicata Linden
Gunnera brephogea Linden & Andr√©
Gunnera perpensa L.
Gunnera pilosa
Gunnera hamiltonii Kirk
Gunnera magellanica
Gunnera magellanica
Gunnera margaretae Schindler
Gunnera magellanica Lam.

Gunnera arenaria Cheeseman ex Kirk
Gunnera pilosa
Gunnera masafuerae
Gunnera manicata
Gunnera magellanica
Gunnera macrophylla
Gunnera chilensis
Gunnera manicata Linden
Gunnera magellanica
Gunnera bracteata
Gunnera mexicana Brandegee
Gunnera
Gunnera magallanica
Gunnera perpensa L.
Gunnera petaloidea
Gunnera killipiana Lundell
Gunnera petaloidea
Gunnera macrophylla
Gunnera chilensis var. valdiviensis
Gunnera pilosa Kunth
Gunnera magellanica Lam.
Gunnera
Gunnera hamiltonii Kirk
Gunnera peltata Phil.
Gunnera magellanica Lam.
Gunnera magellanica Lam.
Gunnera manicata Linden
Gunnera magellanica
Gunnera talamancana
Gunnera brephogea Linden & Andr√©
Gunnera densiflora Hook.f.
Gunnera magallanica
Gunnera chilensis
Gunnera herteri Mattf.
Gunnera prorepens Hook.f.

Gunnera perpensa
Gunnera macrophylla
Gunnera magellanica
Gunnera magellanica
Gunnera perpensa
Gunnera perpensa
Gunnera petaloidea
Gunnera perpensa
Gunnera peltata Phil.
Gunnera brephogea Linden & Andr√©
Gunnera perpensa
Gunnera magellanica
Gunnera magellanica Lam.
Gunnera tayrona L.E. Mora
Gunnera lobata
Gunnera magellanica Lam.
Gunnera magellanica Lam.
Gunnera petaloidea
Gunnera macrophylla Bl.
Gunnera magallanica
Gunnera molokaiensis H. St. John
Gunnera
Gunnera petaloidea
Gunnera petaloidea
Gunnera petaloidea
Gunnera pilosa
Gunnera monoica Raoul
Gunnera prorepens
Gunnera hamiltonii Kirk
Gunnera monoica Raoul
Gunnera perpensa L.
Gunnera manicata Linden
Gunnera magellanica
Gunnera magellanica
Gunnera magellanica

Gunnera magallanica
Gunnera perpensa
Gunnera petaloidea
Gunnera isignis (Oerst.) A.DC.
Gunnera pilosa Kunth
Gunnera bracteata
Gunnera petaloidea
Gunnera petaloidea
Gunnera prorepens Hook.
Gunnera chilensis
Gunnera
Gunnera magellanica
Gunnera hamiltonii
Gunnera petaloidea
Gunnera tinctoria var. valdiviensis
Gunnera monoica
Gunnera petaloidea
Gunnera petaloidea subsp. petaloidea
Gunnera insignis (Oerst.) Oerst.
Gunnera magellanica Lam.
Gunnera brephogea
Gunnera
Gunnera manicata Linden
Gunnera magellanica
Gunnera manicata Linden ex Andr√©
Gunnera perpensa
Gunnera brephogea
Gunnera petaloidea subsp. kauaiensis
Gunnera monoica
Gunnera tamanensis L.E. Mora
Gunnera hamiltonii Kirk
Gunnera eastwoodae H.St.John
Gunnera mexicana
Gunnera prorepens Hook.f.
Gunnera magallanica

Gunnera venezolana subsp. tachirensis
Gunnera tinctoria var. valdiviensis Mora
Gunnera talamancana
Gunnera insignis
Gunnera magellanica Lam.
Gunnera manicata
Gunnera monoica Raoul
Gunnera densiflora Hook.f.
Gunnera pilosa Kunth
Gunnera magallanica
Gunnera magallanica
Gunnera dentata Kirk
Gunnera cordifolia
Gunnera manicata Linden
Gunnera petaloidea
Gunnera monoica Raoul
Gunnera boliviana Morong. ex Rusby
Gunnera eastwoodae
Gunnera pilosa
Gunnera chilensis
Gunnera monoica
Gunnera herteri Osten
Gunnera densiflora Hook.f.
Gunnera tajumbina L. E. Mora
Gunnera hamiltonii Kirk
Gunnera brephogea
Gunnera pilosa
Gunnera macrophylla
Gunnera macrophylla
Gunnera magellanica
Gunnera bracteata
Gunnera magellanica Lam.
Gunnera perpensa L.
Gunnera
Gunnera prorepens

Gunnera perpensa
Gunnera peltata Phil.
Gunnera magellanica
Gunnera magellanica Lam.
Gunnera tajumbina L. E. Mora
Gunnera dentata
Gunnera magallanica
Gunnera herterii Osten
Gunnera macrophylla
Gunnera lobata
Gunnera perpensa
Gunnera magellanica
Gunnera macrophylla
Gunnera plicata
Gunnera brephogea Linden & Andr√©
Gunnera perpensa L.
Gunnera amicorum Ewan
Gunnera magellanica
Gunnera magellanica
Gunnera insignis
Gunnera masafuerae
Gunnera macrophylla
Gunnera peltata
Gunnera mixta Kirk
Gunnera manicata Linden
Gunnera pilosa
Gunnera chilensis
Gunnera magellanica Lam.
Gunnera bracteata Steud. ex Benn.
Gunnera manicata Linden
Gunnera talamancana
Gunnera chilensis Lam.
Gunnera macrophylla
Gunnera insignis
Gunnera petaloidea

Gunnera albocarpa
Gunnera perpensa
Gunnera reichei Schindl.
Gunnera perpensa
Gunnera monoica
Gunnera L.
Gunnera perpensa
Gunnera perpensa
Gunnera perpensa
Gunnera magellanica
Gunnera chilensis
Gunnera manicata Linden ex Delchev.
Gunnera magellanica Lam.
Gunnera monoica Raoul
Gunnera boliviana Morong. ex Rusby
Gunnera L.
Gunnera chilensis
Gunnera petaloidea
Gunnera perpensa L.
Gunnera peltata Phil.
Gunnera bolivari
Gunnera herteri Osten
Gunnera magellanica Lam.
Gunnera colombiana L.E. Mora
Gunnera L.
Gunnera magellanica Lam.
Gunnera magellanica Lam.
Gunnera talamancana H.Weber & L.E.Mora
Gunnera
Gunnera colombiana L.E. Mora
Gunnera talamancana Weber & L. E. Mora
Gunnera perpensa
Gunnera perpensa
Gunnera petaloidea
Gunnera mexicana Brandegee

Gunnera magellanica Lam.
Gunnera magellanica Lam.
Gunnera manicata
Gunnera brephogea
Gunnera mexicana Brandegee
Gunnera insignis Oerst.
Gunnera pilosa Kunth
Gunnera lobata
Gunnera pilosa
Gunnera
Gunnera dentata
Gunnera brephogea Linden & Andr√©
Gunnera dentata
Gunnera monoica
Gunnera petaloidea
Gunnera macrophylla
Gunnera pilosa Kunth
Gunnera magellanica Lam.
Gunnera prorepens Hook.f.
Gunnera perpensa
Gunnera magellanica
Gunnera magellanica
Gunnera cordifolia (Hook.f.) Hook.f.
Gunnera petaloidea
Gunnera petaloidea
Gunnera perpensa
Gunnera perpensa
Gunnera perpensa
Gunnera kaalensis
Gunnera manicata Linden
Gunnera petaloidea
Gunnera magellanica
Gunnera scabra Ruiz & Pav.
Gunnera petaloidea
Gunnera magnifica

Gunnera perpensa
Gunnera perpensa
Gunnera perpensa
Gunnera manicata Linden
Gunnera macrophylla
Gunnera masafuerae
Gunnera petaloidea
Gunnera arenaria Cheeseman ex Kirk
Gunnera mixta Kirk
Gunnera cordifolia (Hook. f.) Hook.
Gunnera bracteata Steudel ex Bennett
Gunnera monoica
Gunnera pilosa
Gunnera petaloidea
Gunnera perpensa
Gunnera apiculata Schindl.
Gunnera manicata Linden
Gunnera manicata Linden
Gunnera pilosa Kunth
Gunnera prorepens
Gunnera magellanica Lam.
Gunnera hamiltonii Kirk
Gunnera petaloidea Gaudich.
Gunnera brephogea Linden & Andr√©
Gunnera perpensa
Gunnera magellanica
Gunnera magellanica
Gunnera magellanica Lam.
Gunnera
Gunnera magellanica Lam.
Gunnera aequatoriensis L.E. Mora
Gunnera perpensa
Gunnera glabra Phil.
Gunnera magellanica
Gunnera monoica

Gunnera brephogea Linden & Andr√©
Gunnera macrophylla Blume
Gunnera petaloidea
Gunnera magellanica
Gunnera insignis
Gunnera columbiana L. E. Mora
Gunnera magellanica Lam.
Gunnera magellanica Lam.
Gunnera magellanica Lam.
Gunnera magellanica Lam.
Gunnera magellanica Lam.
Gunnera magellanica Lam.
Gunnera magellanica Lam.
Gunnera magellanica Lam.
Gunnera magallanica
Gunnera magellanica Lam.
Gunnera magellanica Lam.
Gunnera magellanica Lam.
Gunnera magellanica Lam.
Gunnera magellanica Lam.
Gunnera magellanica Lam.
Gunnera magellanica Lam.
Gunnera lobata
Gunnera lobata Hook.
Gunnera magellanica Lam.
Gunnera lobata Hook.
Gunnera magellanica Lam.
Gunnera magellanica
Gunnera lobata Hook.
Gunnera lobata Hook.
Gunnera magellanica Lam.
Gunnera magellanica Lam.
Gunnera magellanica Lam.
Gunnera magellanica Lam.
Gunnera magellanica Lam.

Gunnera magellanica Lam.
Gunnera magellanica Lam.
Gunnera magellanica Lam.
Gunnera magellanica Lam.
Gunnera magellanica Lam.
Gunnera magellanica
Gunnera magellanica
Gunnera magellanica
Gunnera magellanica Lam.
Gunnera magellanica Lam.
Gunnera magellanica
Gunnera magellanica Lam.
Gunnera magellanica
Gunnera
Gunnera magellanica
Gunnera magellanica
Gunnera
Gunnera
Gunnera
Gunnera lobata Hook.f.
Gunnera magellanica Lam.
Gunnera magellanica Lam.
Gunnera magellanica Lam.
Gunnera magellanica Lam.
Gunnera magellanica Lam.
Gunnera magellanica
Gunnera magellanica
Gunnera magellanica
Gunnera lobata Hook.
Gunnera L.
Gunnera dentata Kirk
Gunnera prorepens Hook.f.
Gunnera magellanica Lam.
Gunnera magellanica Lam.
Gunnera tinctoria Mirb.

Gunnera reicheri Schindl.
Gunnera albocarpa
Gunnera monoica
Gunnera albocarpa
Gunnera albocarpa
Gunnera albocarpa
Gunnera monoica
Gunnera monoica
Gunnera albocarpa
Gunnera monoica
Gunnera monoica
Gunnera albocarpa
Gunnera albocarpa
Gunnera albocarpa
Gunnera albocarpa
Gunnera monoica
Gunnera monoica
Gunnera albocarpa
Gunnera albocarpa
Gunnera prorepens Hook.f.
Gunnera magellanica Lam.
Gunnera albocarpa
Gunnera albocarpa
Gunnera monoica
Gunnera albocarpa
Gunnera albocarpa
Gunnera albocarpa
Gunnera albocarpa
Gunnera albocarpa
Gunnera albocarpa
Gunnera albocarpa
Gunnera albocarpa
Gunnera albocarpa
Gunnera albocarpa
Gunnera albocarpa

Gunnera monoica
Gunnera monica raoul
Gunnera albocarpa
Gunnera albocarpa
Gunnera albocarpa
Gunnera albocarpa
Gunnera albocarpa
Gunnera monoica
Gunnera albocarpa
Gunnera albocarpa
Gunnera monoica
Gunnera monoica
Gunnera monoica
Gunnera monoica
Gunnera monoica
Gunnera monoica
Gunnera monoica
Gunnera monoica
Gunnera monoica
Gunnera monoica
Gunnera monoica
Gunnera monoica
Gunnera monoica
Gunnera monoica
Gunnera monoica
Gunnera monoica
Gunnera √ómixta Kirk
Gunnera monoica
Gunnera monoica
Gunnera monoica
Gunnera monoica
Gunnera monoica Raoul
Gunnera monoica
Gunnera monoica
Gunnera monoica

Gunnera dentata
Gunnera monoica
Gunnera monoica
Gunnera monoica
Gunnera monoica
Gunnera prorepens Hook.f.
Gunnera dentata
Gunnera monoica
Gunnera dentata
Gunnera arenaria
Gunnera monoica
Gunnera monoica
Gunnera monoica
Gunnera monoica
Gunnera monoica
Gunnera monoica
Gunnera prorepens
Gunnera monoica
Gunnera monoica
Gunnera monoica
Gunnera monoica
Gunnera monoica
Gunnera monoica
Gunnera monoica
Gunnera monoica
Gunnera monoica
Gunnera monoica
Gunnera monoica
Gunnera monoica
Gunnera monoica
Gunnera monoica
Gunnera dentata
Gunnera monoica
Gunnera monoica
Gunnera monoica

Gunnera monoica
Gunnera monoica
Gunnera monoica
Gunnera monoica
Gunnera dentata
Gunnera monoica
Gunnera monoica
Gunnera monoica
Gunnera monoica
Gunnera albocarpa
Gunnera monoica
Gunnera monoica
Gunnera monoica Raoul
Gunnera monoica
Gunnera monoica
Gunnera monoica
Gunnera monoica
Gunnera monoica
Gunnera monoica
Gunnera monoica
Gunnera monoica
Gunnera monoica
Gunnera monoica Raoul
Gunnera monoica Raoul
Gunnera dentata
Gunnera monoica
Gunnera monoica
Gunnera monoica
Gunnera monoica
Gunnera monoica Raoul
Gunnera monoica
Gunnera monoica
Gunnera monoica
Gunnera dentata
Gunnera monoica

Gunnera monoica
Gunnera
Gunnera monoica
Gunnera monoica
Gunnera monoica
Gunnera dentata
Gunnera monoica
Gunnera monoica
Gunnera monoica
Gunnera monoica
Gunnera monoica
Gunnera monoica
Gunnera prorepens
Gunnera prorepens
Gunnera monoica
Gunnera albocarpa
Gunnera prorepens
Gunnera dentata
Gunnera prorepens
Gunnera albocarpa
Gunnera prorepens
Gunnera monoica Raoul
Gunnera monoica Raoul
Gunnera monoica Raoul
Gunnera monoica Raoul
Gunnera monoica Raoul
Gunnera monoica Raoul
Gunnera monoica Raoul
Gunnera monoica Raoul
Gunnera monoica Raoul
Gunnera monoica Raoul
Gunnera tinctoria Mirb.
Gunnera magellanica Lam.
Gunnera magellanica Lam.
Gunnera magellanica Lam.

Gunnera magellanica Lam.
Gunnera manicata Lindl.
Gunnera
Gunnera
Gunnera densiflora Hook.f.
Gunnera densiflora Hook.f.
Gunnera prorepens Hook.f.
Gunnera L.
Gunnera monoica Raoul
Gunnera albocarpa (Kirk) Cockayne
Gunnera monoica
Gunnera √ómixta Kirk
Gunnera monoica Raoul
Gunnera prorepens Hook.f.
Gunnera monoica
Gunnera monoica
Gunnera prorepens Hook.f.
Gunnera monoica
Gunnera monoica
Gunnera monoica
Gunnera monoica
Gunnera monoica
Gunnera strigosa
Gunnera monoica
Gunnera dentata
Gunnera monoica
Gunnera monoica
Gunnera monoica
Gunnera monoica
Gunnera monoica
Gunnera monoica
Gunnera monoica
Gunnera monoica
Gunnera monoica Raoul
Gunnera monoica Raoul

Gunnera monoica
Gunnera monoica
Gunnera monoica
Gunnera monoica Raoul
Gunnera monoica Raoul
Gunnera monoica
Gunnera monoica
Gunnera monoica
Gunnera monoica
Gunnera monoica
Gunnera monoica
Gunnera monoica
Gunnera dentata Kirk
Gunnera prorepens Hook.f.
Gunnera monoica Raoul
Gunnera monoica
Gunnera prorepens Hook.f.
Gunnera √ómixta Kirk
Gunnera monoica Raoul
Gunnera monoica Raoul
Gunnera monoica Raoul
Gunnera dentata Kirk
Gunnera chilensis
Gunnera magellanica Lam.
Gunnera magellanica Lam.
Gunnera magellanica Lam.
Gunnera magellanica
Gunnera
Gunnera tinctoria var. meyerii
Gunnera tinctoria var. meyerii
Gunnera magellanica Lam.
Gunnera tinctoria (Molina) Mirbel
Gunnera cordifolia (Hook.f.) Hook.f.
Gunnera cordifolia
Gunnera cordifolia (Hook. f.) Hook.

Gunnera cordifolia
Gunnera cordifolia
Gunnera monoica Raoul
Gunnera monoica Raoul
Gunnera monoica Raoul
Gunnera strigosa
Gunnera albocarpa (Kirk) Cockayne
Gunnera √ómixta Kirk
Gunnera monoica Raoul
Gunnera √ómixta Kirk
Gunnera dentata Kirk
Gunnera prorepens Hook.f.
Gunnera tinctoria Mirb.
Gunnera tinctoria (Mol.) Mirbel
Gunnera tincytoria (Molina) Mirbel
Gunnera tinctoria (Mol.) Mirbel
Gunnera tinctoria (Mol.) Mirbel
Gunnera magellanica Lam.
Gunnera tinctoria (Mol.) Mirbel
Gunnera tinctoria
Gunnera tinctoria Mirb.
Gunnera tincytoria (Molina) Mirbel
Gunnera cordifolia (Hook. f.) Hook.
Gunnera cordifolia (Hook. f.) Hook.
Gunnera cordifolia (Hook. f.) Hook.
Gunnera cordifolia (Hook. f.) Hook.
Gunnera dentata Kirk
Gunnera arenaria Cheeseman
Gunnera monoica
Gunnera monoica
Gunnera monoica
Gunnera magellanica Lam.
Gunnera monoica
Gunnera dentata Kirk
Gunnera dentata Kirk

Gunnera manicata Linden ex Andr√©
Gunnera tinctoria Mirb.
Gunnera tinctoria (Molina) Mirb.
Gunnera
Gunnera magellanica Lam.
Gunnera tinctoria Mirb.
Gunnera flavida Colenso
Gunnera prorepens Hook.f.
Gunnera √óstrigosa Colenso
Gunnera tinctoria (Molina) Mirbel
Gunnera tinctoria (Molina) Mirbel
Gunnera magellanica Lam.
Gunnera magellanica Lam.
Gunnera tinctoria Mirb.
Gunnera tinctoria (Molina) Mirb.
Gunnera berteroi Phil.
Gunnera
Gunnera berteroi Phil.
Gunnera berteroi Phil.
Gunnera berteroi Phil.
Gunnera berteroi Phil.
Gunnera berteroi Phil.
Gunnera berteroi Phil.
Gunnera berteroi Phil.
Gunnera herterii Osten
Gunnera perpensa
Gunnera bracteata Steud. ex J.Benn.
Gunnera peltata Phil.
Gunnera bracteata Steud. ex J.Benn.
Gunnera peltata Phil.
Gunnera peltata Phil.
Gunnera herteri Osten
Gunnera perpensa
Gunnera perpensa
Gunnera perpensa

Gunnera perpensa
Gunnera perpensa
Gunnera perpensa
Gunnera perpensa
Gunnera perpensa
Gunnera perpensa
Gunnera perpensa
Gunnera perpensa
Gunnera perpensa
Gunnera perpensa
Gunnera perpensa
Gunnera perpensa
Gunnera perpensa
Gunnera perpensa
Gunnera perpensa
Gunnera perpensa
Gunnera perpensa
Gunnera perpensa
Gunnera perpensa
Gunnera
Gunnera perpensa
Gunnera perpensa
Gunnera perpensa
Gunnera perpensa
Gunnera perpensa
Gunnera perpensa
Gunnera perpensa
Gunnera perpensa
Gunnera perpensa
Gunnera perpensa
Gunnera perpensa
Gunnera perpensa
Gunnera perpensa
Gunnera perpensa
Gunnera perpensa

Gunnera perpensa
Gunnera perpensa
Gunnera perpensa
Gunnera perpensa
Gunnera perpensa
Gunnera perpensa
Gunnera perpensa
Gunnera perpensa
Gunnera perpensa
Gunnera perpensa
Gunnera perpensa L.
Gunnera perpensa
Gunnera perpensa
Gunnera perpensa
Gunnera perpensa
Gunnera perpensa
Gunnera perpensa
Gunnera perpensa
Gunnera perpensa
Gunnera perpensa
Gunnera perpensa
Gunnera perpensa
Gunnera perpensa
Gunnera perpensa
Gunnera perpensa
Gunnera perpensa
Gunnera perpensa
Gunnera perpensa L.
Gunnera perpensa
Gunnera perpensa
Gunnera perpensa
Gunnera perpensa
Gunnera perpensa
Gunnera perpensa
Gunnera perpensa

Gunnera perpensa
Gunnera perpensa
Gunnera manicata
Gunnera manicata
Gunnera manicata
Gunnera manicata
Gunnera perpense L.
Gunnera perpensa
Gunnera perpensa
Gunnera perpensa
Gunnera perpensa
Gunnera perpensa
Gunnera perpensa
Gunnera perpensa
Gunnera perpensa
Gunnera perpensa
Gunnera perpensa
Gunnera perpensa
Gunnera perpensa
Gunnera perpensa
Gunnera perpensa
Gunnera perpensa
Gunnera perpensa
Gunnera perpensa
Gunnera perpensa
Gunnera perpensa
Gunnera magellanica Lam.
Gunnera perpensa
Gunnera perpensa
Gunnera perpensa
Gunnera perpensa
Gunnera perpensa
Gunnera perpensa
Gunnera perpensa
Gunnera perpensa

Gunnera perpensa
Gunnera perpensa
Gunnera perpensa
Gunnera perpensa
Gunnera perpensa
Gunnera perpensa
Gunnera perpensa
Gunnera perpensa
Gunnera perpensa
Gunnera perpensa
Gunnera perpensa
Gunnera perpensa
Gunnera perpensa
Gunnera perpensa
Gunnera perpensa
Gunnera perpensa
Gunnera perpensa
Gunnera perpensa
Gunnera perpensa
Gunnera perpensa
Gunnera perpensa
Gunnera perpensa
Gunnera perpensa
Gunnera perpensa
Gunnera perpensa
Gunnera perpensa
Gunnera perpensa
Gunnera apiculata Schindler
Gunnera apiculata Schindl.
Gunnera
Gunnera berteroi Phil.
Gunnera perpensa
Gunnera perpensa
Gunnera perpensa
Gunnera perpensa

Gunnera perpensa
Gunnera perpensa
Gunnera perpensa
Gunnera perpensa
Gunnera perpensa
Gunnera apiculata Schindl.
Gunnera perpensa
Gunnera L.
Gunnera margaretae Schindler
Gunnera apiculata Schindl.
Gunnera margaretae Schindler
Gunnera schindleri L.E. Mora
Gunnera schindleri L.E.Mora
Gunnera apiculata Schindl.
Gunnera L.
Gunnera annae Schindl.
Gunnera annae Schindl.
Gunnera annae Schindl.
Gunnera
Gunnera L.
Gunnera L.
Gunnera L.
Gunnera L.
Gunnera L.
Gunnera magellanica Lam.
Gunnera magellanica Lam.
Gunnera annae Schindl.
Gunnera apiculata Schindl.
Gunnera L.
Gunnera L.
Gunnera margaretae Schindler
Gunnera peruviana J.F. Macbr.
Gunnera magellanica Lam.
Gunnera magellanica Lam.
Gunnera berteroi Phil.

Gunnera scabra Ruiz & Pav.
Gunnera magellanica Lam.
Gunnera peruviana J.F. Macbr.
Gunnera boliviana Morong. ex Rusby
Gunnera boliviana Morong. ex Rusby
Gunnera L.
Gunnera magellanica Lam.
Gunnera L.
Gunnera magellanica Lam.
Gunnera magellanica Lam.
Gunnera magellanica Lam.
Gunnera L.
Gunnera berteroi Phil.
Gunnera peruviana J.F. Macbr.
Gunnera L.
Gunnera L.
Gunnera L.
Gunnera L.
Gunnera L.
Gunnera bolivari J.F. Macbr.
Gunnera L.
Gunnera L.
Gunnera L.
Gunnera L.
Gunnera L.
Gunnera L.
Gunnera L.
Gunnera brephogea subsp. magna L.E. Mora
Gunnera L.
Gunnera L.
Gunnera L.
Gunnera L.
Gunnera L.
Gunnera perpensa
Gunnera annae Schindl.

Gunnera annae Schindl.
Gunnera scabra Ru√≠z & Pav.
Gunnera annae Schindl.
Gunnera bolivari J. F. Macbr.
Gunnera peruviana J. F. Macbr.
Gunnera peruviana J. F. Macbr.
Gunnera perpensa L.
Gunnera perpensa L.
Gunnera perpensa
Gunnera perpensa
Gunnera macrophylla
Gunnera macrophylla
Gunnera macrophylla
Gunnera macrophylla
Gunnera macrophylla
Gunnera macrophylla
Gunnera macrophylla
Gunnera macrophylla
Gunnera macrophylla
Gunnera macrophylla
Gunnera
Gunnera macrophylla
Gunnera macrophylla
Gunnera macrophylla
Gunnera
Gunnera macrophylla
Gunnera L.
Gunnera L.
Gunnera L.
Gunnera
Gunnera macrophylla Blume
Gunnera macrophylla
Gunnera macrophylla
Gunnera macrophylla
Gunnera macrophylla Blume

Gunnera macrophylla
Gunnera
Gunnera
Gunnera
Gunnera
Gunnera macrophylla
Gunnera macrophylla
Gunnera macrophylla
Gunnera macrophylla
Gunnera annae Schindl.
Gunnera brephogea subsp. magna L.E. Mora
Gunnera bolivari J.F. Macbr.
Gunnera L.
Gunnera L.
Gunnera macrophylla
Gunnera
Gunnera
Gunnera
Gunnera macrophylla
Gunnera
Gunnera
Gunnera macrophylla
Gunnera macrophylla Blume
Gunnera L.
Gunnera macrophylla
Gunnera L.
Gunnera macrophylla Blume
Gunnera macrophylla
Gunnera
Gunnera
Gunnera
Gunnera L.
Gunnera brephogea Linden & Andr√©
Gunnera L.
Gunnera pilosa Kunth

Gunnera
Gunnera L.
Gunnera L.
Gunnera L.
Gunnera
Gunnera macrophylla
Gunnera macrophylla
Gunnera macrophylla
Gunnera macrophylla
Gunnera macrophylla
Gunnera macrophylla
Gunnera macrophylla
Gunnera macrophylla
Gunnera macrophylla Bl.
Gunnera L.
Gunnera L.
Gunnera L.
Gunnera annae Schindl.
Gunnera
Gunnera
Gunnera atropurpurea L.E. Mora
Gunnera magellanica Lam.
Gunnera
Gunnera brephogea Linden & Andr√©
Gunnera magellanica Lam.
Gunnera L.
Gunnera magellanica Lam.
Gunnera L.
Gunnera L.
Gunnera
Gunnera magellanica Lam.
Gunnera L.
Gunnera L.
Gunnera magellanica Lam.
Gunnera L.

Gunnera pilosa Kunth
Gunnera atropurpurea var. atropurpurea
Gunnera L.
Gunnera magellanica Lam.
Gunnera magellanica Lam.
Gunnera
Gunnera magellanica Lam.
Gunnera magellanica Lam.
Gunnera
Gunnera magellanica Lam.
Gunnera
Gunnera L.
Gunnera
Gunnera perpensa L.
Gunnera macrophylla
Gunnera L.
Gunnera L.
Gunnera brephogea Linden & Andr√©
Gunnera
Gunnera L.
Gunnera brephogea Linden & Andr√©
Gunnera magallanica
Gunnera pilosa Kunth
Gunnera atropurpurea L. E. Mora
Gunnera pilosa Kunth
Gunnera
Gunnera L.
Gunnera
Gunnera L.
Gunnera L.
Gunnera L.
Gunnera L.
Gunnera L.
Gunnera magellanica Lam.
Gunnera magellanica Lam.

Gunnera magallanica
Gunnera L.
Gunnera magellanica Lam.
Gunnera L.
Gunnera magellanica Lam.
Gunnera magellanica Lam.
Gunnera L.
Gunnera magellanica Lam.
Gunnera L.
Gunnera
Gunnera magellanica Lam.
Gunnera
Gunnera magellanica Lam.
Gunnera magellanica Lam.
Gunnera magellanica Lam.
Gunnera
Gunnera magellanica Lam.
Gunnera magellanica Lam.
Gunnera brephogea Linden & Andr√©
Gunnera magellanica Lam.
Gunnera
Gunnera
Gunnera pilosa Kunth
Gunnera L.
Gunnera L.
Gunnera magellanica Lam.
Gunnera magellanica Lam.
Gunnera magellanica Lam.
Gunnera magellanica Lam.
Gunnera magellanica Lam.
Gunnera magellanica Lam.
Gunnera magellanica Lam.
Gunnera magellanica Lam.
Gunnera magellanica Lam.
Gunnera

Gunnera magellanica Lam.
Gunnera L.
Gunnera magellanica Lam.
Gunnera brephogea Linden & Andr√©
Gunnera magellanica Lam.
Gunnera L.
Gunnera magellanica Lam.
Gunnera L.
Gunnera magellanica Lam.
Gunnera magellanica Lam.
Gunnera pilosa Kunth
Gunnera
Gunnera magellanica Lam.
Gunnera magellanica Lam.
Gunnera magellanica Lam.
Gunnera brephogea Linden & Andr√©
Gunnera magellanica Lam.
Gunnera brephogea Linden & Andr√©
Gunnera
Gunnera macrophylla
Gunnera
Gunnera
Gunnera macrophylla
Gunnera
Gunnera brephogea subsp. magna L.E. Mora
Gunnera L.
Gunnera magellanica Lam.
Gunnera L.
Gunnera L.
Gunnera magellanica Lam.
Gunnera magellanica Lam.
Gunnera brephogea Linden & Andr√©
Gunnera L.
Gunnera magellanica Lam.
Gunnera magellanica Lam.

Gunnera brephogea Linden & Andr√©
Gunnera magellanica Lam.
Gunnera magellanica Lam.
Gunnera magellanica Lam.
Gunnera magellanica Lam.
Gunnera brephogea Linden & Andr√©
Gunnera magellanica Lam.
Gunnera magellanica Lam.
Gunnera magellanica Lam.
Gunnera magellanica Lam.
Gunnera magellanica Lam.
Gunnera magellanica Lam.
Gunnera magellanica Lam.
Gunnera magellanica Lam.
Gunnera magellanica Lam.
Gunnera magellanica Lam.
Gunnera magellanica Lam.
Gunnera
Gunnera magellanica Lam.
Gunnera magellanica Lam.
Gunnera
Gunnera
Gunnera magellanica Lam.
Gunnera magellanica Lam.
Gunnera magellanica Lam.
Gunnera magellanica Lam.
Gunnera magellanica Lam.
Gunnera L.
Gunnera magellanica Lam.
Gunnera magellanica Lam.
Gunnera magellanica Lam.
Gunnera
Gunnera magallanica
Gunnera L.
Gunnera magellanica Lam.

Gunnera magellanica Lam.
Gunnera L.
Gunnera magellanica Lam.
Gunnera magellanica Lam.
Gunnera magellanica Lam.
Gunnera L.
Gunnera magellanica Lam.
Gunnera L.
Gunnera magellanica Lam.
Gunnera magellanica Lam.
Gunnera magellanica Lam.
Gunnera L.
Gunnera L.
Gunnera brephogea Linden & Andr√©
Gunnera L.
Gunnera L.
Gunnera L.
Gunnera brephogea Linden & Andr√©
Gunnera L.
Gunnera
Gunnera
Gunnera
Gunnera pilosa Kunth
Gunnera L.
Gunnera
Gunnera
Gunnera L.
Gunnera brephogea Linden & Andr√©
Gunnera brephogea Linden & Andr√©
Gunnera L.
Gunnera
Gunnera L.
Gunnera L.
Gunnera L.
Gunnera ecuadoriana Gilli

Gunnera magellanica Lam.
Gunnera L.
Gunnera brephogea Linden & Andr√©
Gunnera
Gunnera L.
Gunnera L.
Gunnera
Gunnera brephogea Linden & Andr√©
Gunnera magellanica Lam.
Gunnera L.
Gunnera magellanica Lam.
Gunnera L.
Gunnera L.
Gunnera L.
Gunnera magellanica Lam.
Gunnera brephogea Linden & Andr√©
Gunnera L.
Gunnera magellanica Lam.
Gunnera magellanica Lam.
Gunnera L.
Gunnera magellanica Lam.
Gunnera magellanica Lam.
Gunnera magellanica Lam.
Gunnera magellanica Lam.
Gunnera magellanica Lam.
Gunnera magellanica Lam.
Gunnera quitoensis L. E. Mora
Gunnera magellanica Lam.
Gunnera magellanica Lam.
Gunnera magellanica Lam.
Gunnera brephogea Linden & Andr√©
Gunnera L.
Gunnera magellanica Lam.
Gunnera magellanica Lam.
Gunnera magellanica Lam.

Gunnera magellanica Lam.
Gunnera magellanica Lam.
Gunnera magellanica Lam.
Gunnera L.
Gunnera pilosa
Gunnera pilosa
Gunnera magellanica Lam.
Gunnera magellanica Lam.
Gunnera magellanica Lam.
Gunnera magellanica Lam.
Gunnera colombiana
Gunnera L.
Gunnera
Gunnera
Gunnera
Gunnera L.
Gunnera pilosa
Gunnera pilosa
Gunnera atropurpurea var. munchicana L.E. Mora
Gunnera pilosa
Gunnera magellanica
Gunnera atropurpurea var. munchicana
Gunnera
Gunnera magnifica H. St. John
Gunnera magnifica H. St. John
Gunnera magnifica H. St. John
Gunnera magnifica H. St. John
Gunnera magnifica H. St. John
Gunnera magnifica H. St. John
Gunnera magnifica H. St. John
Gunnera magnifica
Gunnera brephogea
Gunnera brephogea Linden & Andr√©
Gunnera
Gunnera brephogea Linden & Andr√©

Gunnera magnifica
Gunnera magellanica
Gunnera
Gunnera magnifica
Gunnera brephogea Linden & Andr√©
Gunnera magnifica
Gunnera brephogea Linden & Andr√©
Gunnera brephogea
Gunnera magnifica
Gunnera magnifica
Gunnera brephogea Linden & Andr√©
Gunnera brephogea
Gunnera magnifica
Gunnera magnifica
Gunnera magnifica
Gunnera magnifica
Gunnera brephogea
Gunnera magnifica
Gunnera magnifica
Gunnera magnifica
Gunnera
Gunnera brephogea Linden & Andr√©
Gunnera magnifica
Gunnera
Gunnera magnifica
Gunnera brephogea
Gunnera magnifica
Gunnera pilosa
Gunnera brephogea
Gunnera magnifica
Gunnera pilosa Kunth
Gunnera
Gunnera pilosa
Gunnera pilosa
Gunnera bogotana

Gunnera bogotana L.E. Mora
Gunnera bogotana L.E. Mora
Gunnera bogotana L.E. Mora
Gunnera bogotana L.E. Mora
Gunnera bogotana
Gunnera bogotana
Gunnera bogotana
Gunnera bogotana
Gunnera bogotana
Gunnera bogotana
Gunnera bogotana
Gunnera bogotana
Gunnera bogotana
Gunnera bogotana L.E. Mora
Gunnera bogotana
Gunnera bogotana
Gunnera bogotana L.E. Mora
Gunnera pilosa
Gunnera bogotana
Gunnera bogotana
Gunnera bogotana
Gunnera bogotana
Gunnera bogotana L.E. Mora
Gunnera bogotana
Gunnera schultesii
Gunnera pilosa
Gunnera bogotana L.E. Mora
Gunnera bogotana L.E. Mora
Gunnera bogotana
Gunnera bogotana L.E. Mora
Gunnera bogotana
Gunnera bogotana
Gunnera bogotana
Gunnera bogotana L.E. Mora
Gunnera pilosa

Gunnera bogotana
Gunnera bogotana
Gunnera bogotana L.E. Mora
Gunnera bogotana
Gunnera schultesii
Gunnera bogotana L.E. Mora
Gunnera bogotana
Gunnera bogotana
Gunnera bogotana L.E. Mora
Gunnera bogotana
Gunnera bogotana
Gunnera bogotana L.E. Mora
Gunnera bogotana L.E. Mora
Gunnera bogotana L.E. Mora
Gunnera bogotana
Gunnera bogotana
Gunnera bogotana L.E. Mora
Gunnera bogotana L.E. Mora
Gunnera schultesii
Gunnera bogotana
Gunnera bogotana
Gunnera bogotana
Gunnera bogotana L.E. Mora
Gunnera bogotana
Gunnera bogotana L.E. Mora
Gunnera bogotana
Gunnera bogotana
Gunnera bogotana
Gunnera bogotana
Gunnera bogotana L.E. Mora
Gunnera bogotana L.E. Mora
Gunnera bogotana
Gunnera bogotana L.E. Mora
Gunnera bogotana
Gunnera bogotana L.E. Mora

Gunnera bogotana L.E. Mora
Gunnera bogotana
Gunnera bogotana L.E. Mora
Gunnera bogotana L.E. Mora
Gunnera bogotana
Gunnera bogotana
Gunnera bogotana
Gunnera bogotana L.E. Mora
Gunnera bogotana L.E. Mora
Gunnera bogotana
Gunnera bogotana
Gunnera bogotana
Gunnera bogotana
Gunnera bogotana L.E. Mora
Gunnera bogotana L.E. Mora
Gunnera bogotana
Gunnera bogotana L.E. Mora
Gunnera bogotana
Gunnera bogotana
Gunnera bogotana
Gunnera bogotana
Gunnera bogotana
Gunnera schultesii
Gunnera bogotana
Gunnera bogotana
Gunnera bogotana L.E. Mora
Gunnera bogotana
Gunnera bogotana
Gunnera bogotana L.E. Mora
Gunnera bogotana L.E. Mora
Gunnera bogotana
Gunnera bogotana L.E. Mora
Gunnera bogotana L.E. Mora
Gunnera bogotana
Gunnera bogotana L.E. Mora

Gunnera bogotana L.E. Mora
Gunnera pilosa
Gunnera bogotana
Gunnera bogotana L.E. Mora
Gunnera magellanica
Gunnera antioquensis
Gunnera antioquensis
Gunnera antioquensis L.E. Mora
Gunnera L.
Gunnera brephogea Linden & Andr√©
Gunnera brephogea
Gunnera
Gunnera
Gunnera pilosa
Gunnera
Gunnera schultesii
Gunnera
Gunnera brephogea Linden & Andr√©
Gunnera pilosa Kunth
Gunnera pilosa
Gunnera L.
Gunnera L.
Gunnera brephogea Linden & Andr√©
Gunnera pilosa Kunth
Gunnera L.
Gunnera pilosa Kunth
Gunnera L.
Gunnera brephogea
Gunnera pilosa
Gunnera L.
Gunnera L.
Gunnera L.
Gunnera lozanii
Gunnera lozanii
Gunnera lozanii

Gunnera lozanii
Gunnera lozanii
Gunnera lozanii
Gunnera pilosa Kunth
Gunnera pilosa
Gunnera insignis (Oerst.) A. DC.
Gunnera L.
Gunnera pittierana V. M. Badillo & Steyerm.
Gunnera talamancana H. Weber & L.E. Mora
Gunnera insignis (Oerst.) A. DC.
Gunnera talamancana H. Weber & L.E. Mora
Gunnera insignis (Oerst.) A. DC.
Gunnera insignis (Oerst.) A. DC.
Gunnera insignis (Oerst.) A. DC.
Gunnera insignis (Oerst.) A. DC.
Gunnera insignis Oerst.
Gunnera insignis (Oerst.) A. DC.
Gunnera insignis (Oerst.) A. DC.
Gunnera insignis (Oerst.) A. DC.
Gunnera talamancana H. Weber & L.E. Mora
Gunnera insignis (Oerst.) A. DC.
Gunnera insignis (Oerst.) A. DC.
Gunnera insignis (Oerst.) A. DC.
Gunnera L.
Gunnera insignis Oerst.
Gunnera insignis (Oerst.) A. DC.
Gunnera insignis (Oerst.) A. DC.
Gunnera insignis (Oerst.) A. DC.
Gunnera insignis (Oerst.) A. DC.
Gunnera L.
Gunnera brephogea Linden & Andr√©
Gunnera L.
Gunnera venezolana L. E. Mora
Gunnera insignis (Oerst.) A. DC.
Gunnera talamancana

Gunnera insignis
Gunnera insignis
Gunnera insignis
Gunnera insignis
Gunnera L.
Gunnera talamancana
Gunnera insignis
Gunnera talamancana H. Weber & L.E. Mora
Gunnera insignis
Gunnera insignis
Gunnera talamancana H.Weber & L.E.Mora
Gunnera insignis
Gunnera insignis (Oerst.) A. DC.
Gunnera L.
Gunnera talamancana
Gunnera insignis
Gunnera insignis (Oerst.) A. DC.
Gunnera insignis
Gunnera insignis
Gunnera talamancana
Gunnera talamancana
Gunnera talamancana
Gunnera talamancana H. Weber & L.E. Mora
Gunnera talamancana
Gunnera talamancana
Gunnera talamancana
Gunnera insignis
Gunnera talamancana H. Weber & L.E. Mora
Gunnera insignis
Gunnera talamancana
Gunnera talamancana
Gunnera talamancana
Gunnera talamancana H. Weber & L. E. Mora
Gunnera talamancana
Gunnera talamancana H. Weber & L.E. Mora

Gunnera insignis (Oerst.) A. DC.
Gunnera talamancana H. Weber & L.E. Mora
Gunnera insignis
Gunnera insignis
Gunnera talamancana
Gunnera insignis (Oerst.) A. DC.
Gunnera talamancana H. Weber & L.E. Mora
Gunnera talamancana
Gunnera insignis (Oerst.) A. DC.
Gunnera talamancana H. Weber & L.E. Mora
Gunnera insignis (Oerst.) A. DC.
Gunnera talamancana
Gunnera talamancana
Gunnera talamancana
Gunnera insignis
Gunnera talamancana H.Weber & L.E.Mora
Gunnera talamancana
Gunnera talamancana H. Weber & L. E. Mora
Gunnera insignis (Oerst.) A. DC.
Gunnera talamancana H. Weber & L.E. Mora
Gunnera insignis (Oerst.) A. DC.
Gunnera insignis (Oerst.) A. DC.
Gunnera talamancana
Gunnera talamancana
Gunnera insignis (Oerst.) A. DC.
Gunnera talamancana H. Weber & L.E. Mora
Gunnera insignis
Gunnera talamancana
Gunnera insignis
Gunnera insignis
Gunnera insignis
Gunnera talamancana
Gunnera insignis
Gunnera insignis
Gunnera talamancana

Gunnera
Gunnera insignis
Gunnera talamancana
Gunnera insignis
Gunnera talamancana
Gunnera talamancana H. Weber & L.E. Mora
Gunnera talamancana H. Weber & L.E. Mora
Gunnera insignis
Gunnera insignis
Gunnera √ókatherine-wilsoniae L.D. G√≥mez
Gunnera insignis (Oerst.) A. DC.
Gunnera talamancana H. Weber & L.E. Mora
Gunnera insignis (Oerst.) A. DC.
Gunnera insignis (Oerst.) A. DC.
Gunnera talamancana H. Weber & L.E. Mora
Gunnera insignis
Gunnera insignis
Gunnera insignis (Oerst.) A. DC.
Gunnera insignis
Gunnera insignis
Gunnera insignis (Oerst.) A. DC.
Gunnera talamancana H. Weber & L.E. Mora
Gunnera talamancana
Gunnera L.
Gunnera talamancana H. Weber & L.E. Mora
Gunnera talamancana
Gunnera talamancana
Gunnera talamancana
Gunnera insignis (Oerst.) A. DC.
Gunnera insignis
Gunnera talamancana H. Weber & L.E. Mora
Gunnera talamancana
Gunnera talamancana
Gunnera talamancana H. Weber & L.E. Mora
Gunnera talamancana H. Weber & L.E. Mora

Gunnera talamancana H. Weber & L.E. Mora
Gunnera insignis
Gunnera venezolana subsp. venezolana
Gunnera venezolana subsp. venezolana
Gunnera L.
Gunnera venezolana L. E. Mora
Gunnera venezolana L. E. Mora
Gunnera perpensa
Gunnera perpensa L.
Gunnera perpensa L.
Gunnera perpensa L.
Gunnera perpensa L.
Gunnera perpensa L.
Gunnera perpensa L.
Gunnera perpensa L.
Gunnera perpensa L.
Gunnera perpensa L.
Gunnera perpensa L.
Gunnera insignis
Gunnera insignis (Oerst.) A. DC.
Gunnera insignis
Gunnera insignis
Gunnera insignis
Gunnera insignis (Oerst.) A. DC.
Gunnera insignis
Gunnera insignis
Gunnera insignis (Oerst.) A. DC.
Gunnera insignis (Oerst.) A. DC.
Gunnera insignis
Gunnera insignis
Gunnera insignis
Gunnera insignis
Gunnera insignis
Gunnera insignis
Gunnera insignis

Gunnera L.
Gunnera
Gunnera insignis
Gunnera insignis (Oerst.) A. DC.
Gunnera insignis (Oerst.) A. DC.
Gunnera insignis (Oerst.) A. DC.
Gunnera insignis (Oerst.) A. DC.
Gunnera insignis
Gunnera talamancana
Gunnera
Gunnera insignis (Oerst.) A. DC.
Gunnera insignis
Gunnera insignis (Oerst.) A. DC.
Gunnera insignis (Oerst.) A. DC.
Gunnera insignis
Gunnera insignis
Gunnera insignis
Gunnera insignis (Oerst.) A. DC.
Gunnera insignis f. albovariegata L. D. G√≥mez
Gunnera insignis
Gunnera L.
Gunnera
Gunnera tayrona L.E. Mora
Gunnera insignis (Oerst.) A. DC.
Gunnera insignis (Oerst.) A. DC.
Gunnera insignis (Oerst.) A. DC.
Gunnera insignis (Oerst.) A. DC.
Gunnera tayrona
Gunnera tayrona
Gunnera tayrona
Gunnera tayrona
Gunnera tayrona
Gunnera tayrona
Gunnera tayrona
Gunnera tayrona

Gunnera tayrona
Gunnera tayrona
Gunnera tayrona
Gunnera tayrona
Gunnera tayrona
Gunnera tayrona
Gunnera tayrona
Gunnera tayrona
Gunnera tayrona
Gunnera tayrona
Gunnera tayrona
Gunnera tayrona
Gunnera tayrona
Gunnera tayrona
Gunnera tayrona
Gunnera tayrona
Gunnera tayrona
Gunnera tayrona
Gunnera tayrona
Gunnera tayrona
Gunnera tayrona
Gunnera tayrona
Gunnera tayrona
Gunnera tayrona
Gunnera killipiana Lundell
Gunnera killipiana Lundell
Gunnera killipiana Lundell
Gunnera killipiana Lundell
Gunnera killipiana Lundell
Gunnera mexicana Brandegee
Gunnera mexicana Brandegee
Gunnera mexicana Brandegee
Gunnera L.
Gunnera mexicana Brandegee
Gunnera mexicana Brandegee

Gunnera killipiana Lundell
Gunnera mexicana Brandegee
Gunnera macrophylla
Gunnera mexicana Brandegee
Gunnera killipiana Lundell
Gunnera mexicana Brandegee
Gunnera insignis (Oerst.) A. DC.
Gunnera mexicana Brandegee
Gunnera killipiana Lundell
Gunnera mexicana Brandegee
Gunnera petaloidea
Gunnera mexicana Brandegee
Gunnera mexicana Brandegee
Gunnera mexicana Brandegee
Gunnera mexicana Brandegee
Gunnera mexicana Brandegee
Gunnera mexicana Brandegee
Gunnera mexicana Brandegee
Gunnera mexicana Brandegee
Gunnera
Gunnera mexicana Brandegee
Gunnera mexicana Brandegee
Gunnera mexicana Brandegee
Gunnera mexicana Brandegee
Gunnera mexicana Brandegee
Gunnera mexicana Brandegee
Gunnera L.
Gunnera mexicana Brandegee
Gunnera mexicana Brandegee
Gunnera petaloidea
Gunnera petaloidea
Gunnera petaloidea
Gunnera petaloidea
Gunnera petaloidea
Gunnera petaloidea

Gunnera petaloidea
Gunnera petaloidea
Gunnera petaloidea
Gunnera petaloidea
Gunnera petaloidea
Gunnera petaloidea
Gunnera petaloidea
Gunnera petaloidea
Gunnera petaloidea
Gunnera petaloidea
Gunnera petaloides Gaud.
Gunnera petaloidea Gaud.
Gunnera kauaiensis
Gunnera perpensa L.
Gunnera petaloidea
Gunnera killipiana Lundell
Gunnera manicata Linden
Gunnera
Gunnera manicata
Gunnera manicata
Gunnera manicata
Gunnera
Gunnera
Gunnera manicata
Gunnera
Gunnera
Gunnera manicata
Gunnera
Gunnera
Gunnera
Gunnera manicata
Gunnera manicata
Gunnera
Gunnera manicata
Gunnera manicata

Gunnera manicata
Gunnera manicata
Gunnera manicata
Gunnera manicata
Gunnera manicata
Gunnera manicata
Gunnera manicata
Gunnera manicata
Gunnera manicata
Gunnera manicata
Gunnera manicata
Gunnera manicata
Gunnera
Gunnera manicata
Gunnera manicata
Gunnera manicata
Gunnera manicata
Gunnera
Gunnera
Gunnera manicata
Gunnera manicata
Gunnera
Gunnera manicata
Gunnera manicata
Gunnera manicata
Gunnera manicata
Gunnera manicata
Gunnera
Gunnera manicata
Gunnera manicata
Gunnera
Gunnera
Gunnera manicata
Gunnera
Gunnera manicata

Gunnera manicata
Gunnera manicata
Gunnera
Gunnera manicata
Gunnera
Gunnera manicata
Gunnera manicata
Gunnera manicata
Gunnera manicata
Gunnera manicata
Gunnera manicata
Gunnera manicata
Gunnera manicata
Gunnera manicata
Gunnera manicata
Gunnera manicata
Gunnera manicata
Gunnera manicata
Gunnera manicata
Gunnera
Gunnera manicata
Gunnera manicata
Gunnera
Gunnera manicata
Gunnera manicata
Gunnera manicata
Gunnera manicata
Gunnera
Gunnera manicata
Gunnera manicata
Gunnera manicata
Gunnera manicata
Gunnera manicata
Gunnera
Gunnera

Gunnera manicata
Gunnera manicata
Gunnera manicata
Gunnera manicata
Gunnera manicata
Gunnera manicata
Gunnera magellanica
Gunnera magellanica
Gunnera magellanica
Gunnera magellanica
Gunnera magellanica
Gunnera magellanica
Gunnera manicata
Gunnera magellanica Lam.
Gunnera tinctora
Gunnera magellanica
Gunnera magellanica
Gunnera magellanica
Gunnera magellanica
Gunnera magellanica
Gunnera magellanica
Gunnera magellanica
Gunnera magellanica
Gunnera magellanica
Gunnera magellanica
Gunnera magellanica
Gunnera magellanica
Gunnera magellanica
Gunnera magellanica
