# Supplementary material for: Evolutionary persistence in Gunnera and the contribution of southern plant groups to the tropical Andes biodiversity hotspot
Source: PeerJ. 2018 Mar 16;6:e4388. doi: 10.7717/peerj.4388 (PMC5858603; doi:10.7717/peerj.4388)
Supplement: Table S2 [file peerj-06-4388-s004.pdf]

Table S2. Matrices of dispersal and areas allowed used in BioGeoBEARS for each slide of time (0-30, 30-40 and 40-90 Mya). In the areas allowed matrix a value of zero represents an area that is removed, and a value of 1 an area that is present. In the dispersal matrix, values represent percentages of connectivity between areas. (0= 0% and 1 =100%). Note that probabilities for each geographic range are calculated only for the allowed areas given in the areas allowed matrix (with a value of 1). Ranges are coded as follows: **A** - Northern Andes, **B** - Central Andes, **C** - Southern Andes, **D** - Southern south America, **E** - Central America, **F** - Hawaii, **G** - Africa, **H** - Malay Archipelago and **I** - Tasmania and New Zealand.

0 – 30 Mya

Areas allowed matrix

	**A**	**B**	**C**	**D**	**E**	**F**	**G**	**H**	**I**
**A**	1	1	1	1	1	1	1	1	1
**B**	1	1	1	1	1	1	1	1	1
**C**	1	1	1	1	1	1	1	1	1
**D**	1	1	1	1	1	1	1	1	1
**E**	1	1	1	1	1	1	1	1	1
**F**	1	1	1	1	1	1	1	1	1
**G**	1	1	1	1	1	1	1	1	1
**H**	1	1	1	1	1	1	1	1	1
**I**	1	1	1	1	1	1	1	1	1

Dispersal matrix

	**A**	**B**	**C**	**D**	**E**	**F**	**G**	**H**	**I**
**A**	1	1	1	1	1	1	0.001	1	0.001
**B**	1	1	1	1	1	1	0.001	1	0.001
**C**	1	1	1	1	1	1	0.001	1	0.001
**D**	1	1	1	1	1	1	0.001	1	0.001
**E**	1	1	1	1	1	1	1	1	1
**F**	1	1	1	1	1	1	1	1	1
**G**	0.001	0.001	0.001	0.001	0.001	1	1	1	1
**H**	1	1	1	1	1	1	1	1	1
**I**	0.001	0.001	0.001	0.001	0.001	1	1	1	1

30 – 40 Mya

Areas allowed matrix

	A	B	C	D	E	F	G	H	I
**A**	1	1	1	1	1	0	1	1	1
**B**	1	1	1	1	1	0	1	1	1
**C**	1	1	1	1	1	0	1	1	1
**D**	1	1	1	1	1	0	1	1	1
**E**	1	1	1	1	1	0	1	1	1
**F**	0	0	0	0	0	0	0	0	0
**G**	1	1	1	1	1	0	1	1	1
**H**	1	1	1	1	1	0	1	1	1
**I**	1	1	1	1	1	0	1	1	1

Dispersal matrix

	A	B	C	D	E	F	G	H	I
**A**	1	1	1	1	1	1	0.001	1	1
**B**	1	1	1	1	1	1	0.001	1	1
**C**	1	1	1	1	1	1	0.001	1	1
**D**	1	1	1	1	1	1	0.001	1	1
**E**	1	1	1	1	1	1	1	1	1
**F**	1	1	1	1	1	1	1	1	1
**G**	0.001	0.001	0.001	0.001	0.001	1	1	1	1
**H**	1	1	1	1	1	1	1	1	1
**I**	1	1	1	1	1	1	1	1	1

40 – 90 Mya

Areas allowed matrix

	A	B	C	D	E	F	G	H	I
**A**	0	0	0	0	0	0	0	0	0
**B**	0	0	0	0	0	0	0	0	0
**C**	0	0	1	1	1	0	1	1	1
**D**	0	0	1	1	1	0	1	1	1
**E**	0	0	1	1	1	0	1	1	1
**F**	0	0	0	0	0	0	0	0	0
**G**	0	0	1	1	1	0	1	1	1
**H**	0	0	1	1	1	0	1	1	1
**I**	0	0	1	1	1	0	1	1	1

Dispersal matrix

	A	B	C	D	E	F	G	H	I
**A**	1	1	1	1	1	1	0.001	1	1
**B**	1	1	1	1	1	1	0.001	1	1
**C**	1	1	1	1	1	1	0.001	1	1
**D**	1	1	1	1	1	1	0.001	1	1
**E**	1	1	1	1	1	1	1	1	1
**F**	1	1	1	1	1	1	1	1	1
**G**	0.001	0.001	0.001	0.001	1	1	1	1	1
**H**	1	1	1	1	1	1	1	1	1
**I**	1	1	1	1	1	1	1	1	1
