# Supplementary material for: Evolutionary persistence in Gunnera and the contribution of southern plant groups to the tropical Andes biodiversity hotspot
Source: PeerJ. 2018 Mar 16;6:e4388. doi: 10.7717/peerj.4388 (PMC5858603; doi:10.7717/peerj.4388)
Supplement: Table S3 [file peerj-06-4388-s005.pdf]

Table S3. Results from model comparison of climatic niche evolution. Lambda (λ) follows Pagel (1999) where the p value is derived from a likelihood ratio test with lamda = 0. The Aikaike value shows the probability that each model is the best among those compared (wAIC).

		**λ**			***w* AICc**		
	**BIOCLIMATIC VARIABLES**	**λ**	***p* λ>0**	***p* λ<1**	**BM**	**OU**	**WM**
**Bio1**	Mean Annual Temperature	**0.93**	0.04	0.00	0.01	**0.92**	0.07
**Bio6**	Min Temperature of Coldest Month	0.80	0.27	0.00	0.00	0.30	**0.70**
**Bio8**	Mean Temperature of Wettest Quarter	0.78	0.11	0.00	0.00	**0.77**	0.23
**Bio11**	Mean Temperature of Coldest Quarter	**0.92**	0.01	0.00	0.00	**0.93**	0.07
**Bio12**	Annual Precipitation	0.00	1.00	0.00	0.00	0.49	**0.51**
**Bio16**	Precipitation of Wettest Quarter	**1.00**	0.00	0.51	0.15	**0.85**	0.00
**Bio17**	Precipitation of Driest Quarter	**0.93**	0.02	0.01	0.04	**0.90**	0.07
**Bio18**	Precipitation of Warmest Quarter	0.00	1.00	0.02	0.00	**0.96**	0.04
