# Supplementary material for: Evolutionary persistence in Gunnera and the contribution of southern plant groups to the tropical Andes biodiversity hotspot
Source: PeerJ. 2018 Mar 16;6:e4388. doi: 10.7717/peerj.4388 (PMC5858603; doi:10.7717/peerj.4388)
Supplement: File S1 [file peerj-06-4388-s010.pdf]

Gene	Taxon	GenBank Number
ITS_March_26_2018.sqn	G_arenaria	MH017065
ITS_March_26_2018.sqn	G_atropurpurea	MH017066
ITS_March_26_2018.sqn	G_boliviana	MH017067
ITS_March_26_2018.sqn	G_bracteata	MH017068
ITS_March_26_2018.sqn	G_brephogea	MH017069
ITS_March_26_2018.sqn	G_chilensis	MH017070
ITS_March_26_2018.sqn	G_cordifolia	MH017071
ITS_March_26_2018.sqn	G_densiflora	MH017072
ITS_March_26_2018.sqn	G_dentata	MH017073
ITS_March_26_2018.sqn	G_hamiltonii	MH017074
ITS_March_26_2018.sqn	G_herteri	MH017075
ITS_March_26_2018.sqn	G_insignis	MH017076
ITS_March_26_2018.sqn	G_kauaiensis	MH017077
ITS_March_26_2018.sqn	G_lobata	MH017078
ITS_March_26_2018.sqn	G_macrophylla	MH017079
ITS_March_26_2018.sqn	G_magellanica	MH017080
ITS_March_26_2018.sqn	G_manicata	MH017081
ITS_March_26_2018.sqn	G_masafuerae	MH017082
ITS_March_26_2018.sqn	G_mexicana	MH017083
ITS_March_26_2018.sqn	G_monoica	MH017084
ITS_March_26_2018.sqn	G_morae	MH017085
ITS_March_26_2018.sqn	G_peltata	MH017086
ITS_March_26_2018.sqn	G_perpensa	MH017087
ITS_March_26_2018.sqn	G_petaloidea	MH017088
ITS_March_26_2018.sqn	G_pilosa	MH017089
ITS_March_26_2018.sqn	G_prorepens	MH017090
ITS_March_26_2018.sqn	G_talamancana	MH017091
RPOC01_Feb_21_2017.sqn	G_arenaria	MH017092
RPOC01_Feb_21_2017.sqn	G_atropurpurea	MH017093
RPOC01_Feb_21_2017.sqn	G_boliviana	MH017094
RPOC01_Feb_21_2017.sqn	G_bracteata	MH017095
RPOC01_Feb_21_2017.sqn	G_brephogea	MH017096
RPOC01_Feb_21_2017.sqn	G_chilensis	MH017097
RPOC01_Feb_21_2017.sqn	G_cordifolia	MH017098
RPOC01_Feb_21_2017.sqn	G_densiflora	MH017099
RPOC01_Feb_21_2017.sqn	G_dentata	MH017100
RPOC01_Feb_21_2017.sqn	G_herteri	MH017101
RPOC01_Feb_21_2017.sqn	G_insignis	MH017102
RPOC01_Feb_21_2017.sqn	G_kauaiensis	MH017103
RPOC01_Feb_21_2017.sqn	G_lobata	MH017104
RPOC01_Feb_21_2017.sqn	G_macrophylla	MH017105
RPOC01_Feb_21_2017.sqn	G_magellanica	MH017106
RPOC01_Feb_21_2017.sqn	G_manicata	MH017107
RPOC01_Feb_21_2017.sqn	G_masafuerae	MH017108
RPOC01_Feb_21_2017.sqn	G_mexicana	MH017109
RPOC01_Feb_21_2017.sqn	G_monoica	MH017110
RPOC01_Feb_21_2017.sqn	G_peltata	MH017111
RPOC01_Feb_21_2017.sqn	G_perpensa	MH017112
RPOC01_Feb_21_2017.sqn	G_petaloidea	MH017113

RPOCO1_Feb_21_2017.sqn	G_pilosa	MH017114
RPOCO1_Feb_21_2017.sqn	G_prorepens	MH017115
RPOCO1_Feb_21_2017.sqn	G_talamancana	MH017116
RPS_16_Feb_15.sqn	G_arenaria	MH017117
RPS_16_Feb_15.sqn	G_atropurpurea	MH017118
RPS_16_Feb_15.sqn	G_boliviana	MH017119
RPS_16_Feb_15.sqn	G_brephogea	MH017120
RPS_16_Feb_15.sqn	G_chilensis	MH017121
RPS_16_Feb_15.sqn	G_cordifolia	MH017122
RPS_16_Feb_15.sqn	G_densiflora	MH017123
RPS_16_Feb_15.sqn	G_dentata	MH017124
RPS_16_Feb_15.sqn	G_hamiltonii	MH017125
RPS_16_Feb_15.sqn	G_herteri	MH017126
RPS_16_Feb_15.sqn	G_kauaiensis	MH017127
RPS_16_Feb_15.sqn	G_lobata	MH017128
RPS_16_Feb_15.sqn	G_macrophylla	MH017129
RPS_16_Feb_15.sqn	G_magellanica	MH017130
RPS_16_Feb_15.sqn	G_manicata	MH017131
RPS_16_Feb_15.sqn	G_monoica	MH017132
RPS_16_Feb_15.sqn	G_morae	MH017133
RPS_16_Feb_15.sqn	G_peltata	MH017134
RPS_16_Feb_15.sqn	G_perpensa	MH017135
RPS_16_Feb_15.sqn	G_petaloidea	MH017136
RPS_16_Feb_15.sqn	G_pilosa	MH017137
RPS_16_Feb_15.sqn	G_prorepens	MH017138
RPS_16_Feb_15.sqn	G_talamancana	MH017139
SE4_Feb_22_2018.sqn	G_arenaria	MH017140
SE4_Feb_22_2018.sqn	G_atropurpurea	MH017141
SE4_Feb_22_2018.sqn	G_boliviana	MH017142
SE4_Feb_22_2018.sqn	G_brephogea	MH017143
SE4_Feb_22_2018.sqn	G_chilensis	MH017144
SE4_Feb_22_2018.sqn	G_cordifolia	MH017145
SE4_Feb_22_2018.sqn	G_densiflora	MH017146
SE4_Feb_22_2018.sqn	G_dentata	MH017147
SE4_Feb_22_2018.sqn	G_hamiltonii	MH017148
SE4_Feb_22_2018.sqn	G_herteri	MH017149
SE4_Feb_22_2018.sqn	G_insignis	MH017150
SE4_Feb_22_2018.sqn	G_kauaiensis	MH017151
SE4_Feb_22_2018.sqn	G_macrophylla	MH017152
SE4_Feb_22_2018.sqn	G_magellanica	MH017153
SE4_Feb_22_2018.sqn	G_manicata	MH017154
SE4_Feb_22_2018.sqn	G_mexicana	MH017155
SE4_Feb_22_2018.sqn	G_monoica	MH017156
SE4_Feb_22_2018.sqn	G_morae	MH017157
SE4_Feb_22_2018.sqn	G_peltata	MH017158
SE4_Feb_22_2018.sqn	G_perpensa	MH017159
SE4_Feb_22_2018.sqn	G_petaloidea	MH017160
SE4_Feb_22_2018.sqn	G_pilosa	MH017161
SE4_Feb_22_2018.sqn	G_prorepens	MH017162
SE4_Feb_22_2018.sqn	G_talamancana	MH017163

trnHpsbA_feb_22_2018.sqn	G_arenaria	MH017164
trnHpsbA_feb_22_2018.sqn	G_atropurpurea	MH017165
trnHpsbA_feb_22_2018.sqn	G_boliviana	MH017166
trnHpsbA_feb_22_2018.sqn	G_brephogea	MH017167
trnHpsbA_feb_22_2018.sqn	G_cordifolia	MH017168
trnHpsbA_feb_22_2018.sqn	G_dentata	MH017169
trnHpsbA_feb_22_2018.sqn	G_hamiltonii	MH017170
trnHpsbA_feb_22_2018.sqn	G_kauaiensis	MH017171
trnHpsbA_feb_22_2018.sqn	G_lobata	MH017172
trnHpsbA_feb_22_2018.sqn	G_macrophylla	MH017173
trnHpsbA_feb_22_2018.sqn	G_magellanica	MH017174
trnHpsbA_feb_22_2018.sqn	G_manicata	MH017175
trnHpsbA_feb_22_2018.sqn	G_mexicana	MH017176
trnHpsbA_feb_22_2018.sqn	G_peltata	MH017177
trnHpsbA_feb_22_2018.sqn	G_perpensa	MH017178
trnHpsbA_feb_22_2018.sqn	G_petaloidea	MH017179
ycf5_Feb22_2018.sqn	G_arenaria	MH017180
ycf5_Feb22_2018.sqn	G_atropurpurea	MH017181
ycf5_Feb22_2018.sqn	G_boliviana	MH017182
ycf5_Feb22_2018.sqn	G_brephogea	MH017183
ycf5_Feb22_2018.sqn	G_chilensis	MH017184
ycf5_Feb22_2018.sqn	G_cordifolia	MH017185
ycf5_Feb22_2018.sqn	G_densiflora	MH017186
ycf5_Feb22_2018.sqn	G_dentata	MH017187
ycf5_Feb22_2018.sqn	G_hamiltonii	MH017188
ycf5_Feb22_2018.sqn	G_herteri	MH017189
ycf5_Feb22_2018.sqn	G_insignis	MH017190
ycf5_Feb22_2018.sqn	G_kauaiensis	MH017191
ycf5_Feb22_2018.sqn	G_lobata	MH017192
ycf5_Feb22_2018.sqn	G_macrophylla	MH017193
ycf5_Feb22_2018.sqn	G_magellanica	MH017194
ycf5_Feb22_2018.sqn	G_manicata	MH017195
ycf5_Feb22_2018.sqn	G_mexicana	MH017196
ycf5_Feb22_2018.sqn	G_monoica	MH017197
ycf5_Feb22_2018.sqn	G_peltata	MH017198
ycf5_Feb22_2018.sqn	G_perpensa	MH017199
ycf5_Feb22_2018.sqn	G_pilosa	MH017200
ycf5_Feb22_2018.sqn	G_prorepens	MH017201
ycf5_Feb22_2018.sqn	G_talamancana	MH017202
